# Design and Synthesis of New Chacones Substituted with Azide/Triazole Groups and Analysis of Their Cytotoxicity Towards HeLa Cells

**DOI:** 10.3390/molecules170910331

**Published:** 2012-08-29

**Authors:** Graziele D. da Silva, Marina G. da Silva, Estrela M. P. V. E. Souza, Andersson Barison, Sarah C. Simões, Fernando P. Varotti, Leandro A. Barbosa, Gustavo H. R. Viana, José A. F. P. Villar

**Affiliations:** 1 Laboratório de Síntese Orgânica, Universidade Federal de São João Del Rei, Campus Centro Oeste Dona Lindu, 35.501-296, Divinópolis/MG, Brazil; 2 Laboratório de RMN, Universidade Federal do Paraná, 81.531-990, Curitiba/PR, Brazil; 3 Laboratório de Bioquímica de Parasitos, Universidade Federal de São João Del Rei, Campus Centro Oeste Dona Lindu, 35.501-296, Divinópolis/MG, Brazil; 4 Laboratório de Bioquímica Celular, Universidade Federal de São João Del Rei, Campus Centro Oeste Dona Lindu, 35.501-296, Divinópolis/MG, Brazil

**Keywords:** chalcone, cycloaddition, *O*-alkylation, HeLa cells

## Abstract

A series of new chalcones substituted with azide/triazole groups were designed and synthesized, and their cytotoxic activity was evaluated *in vitro* against the HeLa cell line. *O*-Alkylation, Claisen-Schmidt condensation and Cu(I)-catalyzed cycloaddition of azides with terminal alkynes were applied in key steps. Fifteen compounds were tested against HeLa cells. Compound **8c** was the most active molecule, with an IC_50_ value of 13.03 µM, similar to the value of cisplatin (7.37 µM).

## 1. Introduction

Cervical cancer is an important cause of death in women in developing and underdeveloped countries. In Brazil, it has been estimated that there will be 17,540 new cases of cervical cancer in 2012, and this disease causes some 5.000 deaths annually [1]. The treatment of cervical cancer depends on the location, size and histological type of the tumor, as well as the age and general health of the patient [2]. Chemotherapy is indicated concomitant with radiotherapy for the treatment of cervical cancer because of chemotherapy’s radiosensitising effects. Chemotherapy is also used in case of recurrence, when surgery and radiotherapy are not possible [3].

There is now a consensus, based on the results of randomized clinical trials, that concomitant chemotherapy and radiotherapy should be the treatment of choice for stages IIB, III and IVA [4], and the current chemotherapy applied is platinum based. Thus, there is an urgent need to identify new drugs capable of inhibiting the growth of these tumor cells.

Chalcones (1,3-diphenyl-2-propen-1-ones) are an important class of natural products. They are found in plants and are considered to be precursors of flavonoids and isoflavonoids [5]. Previous publications indicate that many natural and synthetic chalcones exhibit a wide range of biological activities, such as antimalarial [6,7], antileishmanial [8], antichagasic [9] and antitumoral effects [10]. Licochalcone A is an example of a naturally occurring chalcone with antitumoral activity that is able to inhibit the growth of human gastric [11], colon [12], and prostate [13] cancer cells.

Recent works have explored the introduction of heterocycles into the structure of chalcones to obtain lead compounds for the development of new therapeutic agents. Chalcones substituted with imidazoles [14], triazoles [15], amines [16] and, more recently, stilbene [17] have been reported to be potential antimalarial compounds. A similar approach was performed with chalcones substituted with a pyrrolobenzodiazepine group through a triazole ring. These compounds were evaluated as multi-target anticancer agents [18].

The interest in molecules containing the 1,2,3-triazole group as chemotherapeutic agents for various diseases is growing because this class of heterocycles is known to exhibit wide range of biological activity, such as antibiotic [19], antifungal [20], and anticancer properties [21].

Chalcones can be easily synthesized by cross aldol condensation (Claisen–Schmidt) between an acetophenone derivative and an aromatic aldehyde. There are several methods to synthesize chalcones that employ different solvents and bases and either conventional heating or microwave irradiation [22].

Herein we present an initial study of the synthesis of some new chalcones substituted with an azide or triazole group and of the cytotoxicity of these new compounds against the HeLa cell line.

## 2. Results and Discussion

**Figure 1:**
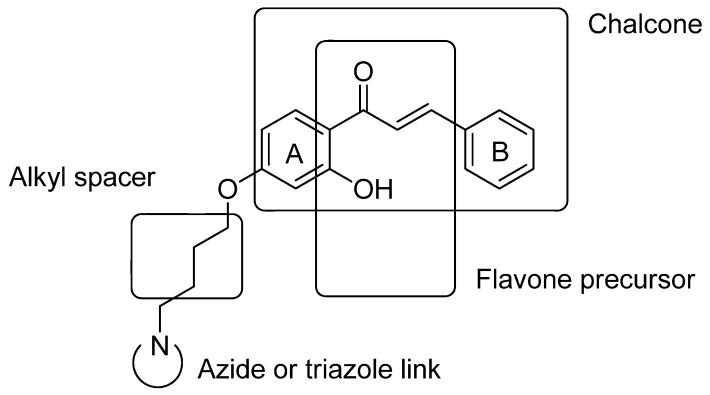
Chalcones proposed in this work.

The goal of our synthesis was to introduce an azide/triazole group connected to ring A of the chalcone by an alkyl spacer (Figure 1). Our chalcone derivatives also possess a hydroxyl group attached to ring A in the 2 position, which makes these compounds flavone precursors. Flavones are an important class which possesses well known biological activities.

**Scheme 1:**
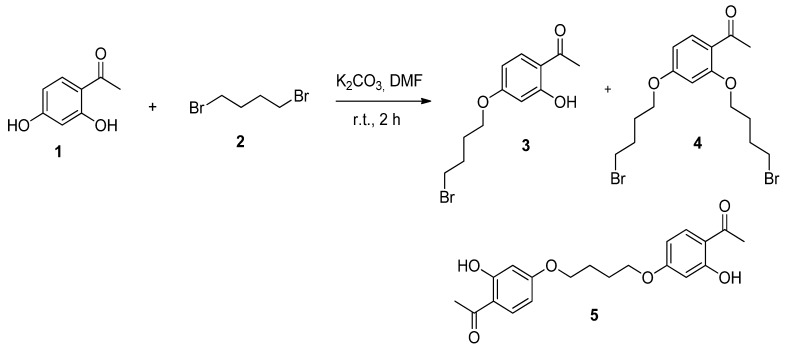
*O*-Alkylation of **1** with 1,4-dibromobutane (**2**).

Monoalkylated resacetophenone **3** was synthesized by *O*-alkylation of resacetophenone (**1**) with 1,4-dibromobutane (**2**). For the first reaction, we used *N*,*N*-dimethylformamide (DMF) and K_2_CO_3_ at room temperature. After preparative separation, three compounds were obtained and identified as 4-mono-*O*-alkylated **3**, 2,4-di-*O*-alkylated **4** and dimer **5** (Scheme 1).

**Figure 2:**
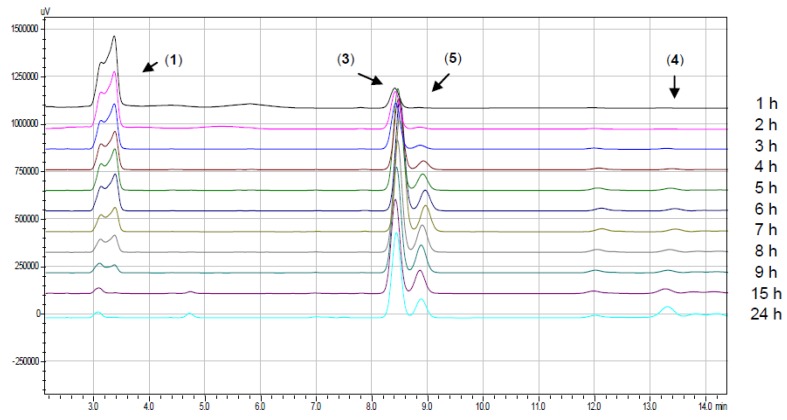
Chromatograms showing the formation of *O*-alkylated products **3**, **4** and **5** (ACN/40 °C, entry 4, Table 1).

To optimize the production of the desired 4-mono-*O*-alkylated **3**, several reaction conditions were tested, and each reaction was monitored by HPLC analysis (Table 1). When dimethylsulfoxide (DMSO) or DMF were used as the solvent, 2,4-di-*O*-alkylated product **4** was produced selectively, although when we used acetonitrile at room temperature, we obtained product **3** in good yield after 48 h (Table 1, entry 3). However, we observed that when the reaction was performed at 40 °C, product **3** was produced selectively in 15 h (Table 1, Figure 2). We also observed that when the 2,4-di-*O*-alkylated compound **4** was produced in large amounts, it was difficult to separate from product **3** by preparative column chromatography. Therefore, we stopped the reaction after 5 h to selectivity produce **3** in good yield and to make the purification process easier.

**Table 1:** Conversion rate of *O*-alkylation of **1**.

Entry	Condition	3	4	5
1	DMF/r.t./2 h	21.8	74.4	3.8
2	DMSO/r.t./2 h	15.8	82.0	2.2
3	ACN/r.t./48 h	71.1	3.2	10.0
4	ACN/40 °C/15 h	71.6	2.1	13.1

**Scheme 2:**
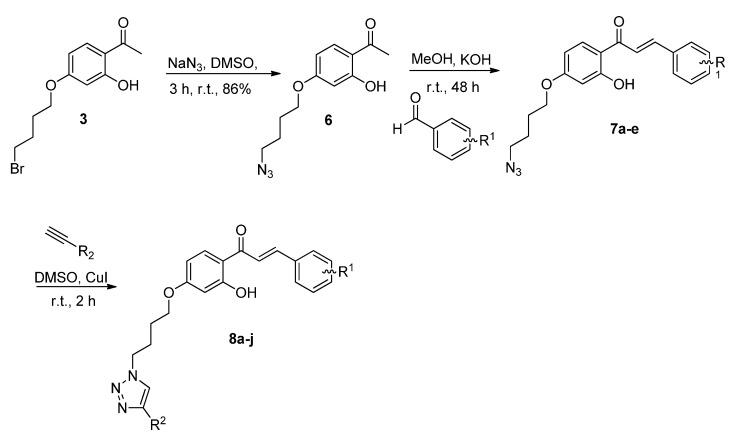
Route for the synthesis of chalcone azides **7a–e** and triazole chalcones **8a–j**.

The selectivity of the *O*-alkylation at position 4 of resacetophenone (**1**) can be explained because there is an intramolecular hydrogen bond between the carbonyl group and the hydrogen of the hydroxyl group in position 2 of the aromatic ring. The hydrogen bond prevents the alkylation of phenol group in position 2 from occuring. Furthermore, the different polarity of the solvents influences directly the reaction. For example, the use of more polar solvents, such as DMF or DMSO, decreased the intensity of the hydrogen bond, allowing the formation of the dialkylated product. However, when we used acetonitrile (a less polar solvent) the major product was the monoalkylated **3**. Azide **6** was obtained from bromide **3** in 86% yield by reaction with sodium azide [23]. In the next step, chalcone derivatives were synthesized by Claisen–Schmidt condensation with different aromatic aldehydes [24] (Scheme 2). This reaction yielded chalcone azide compounds **7a–e** in moderate to good yields after purification by recrystallization from MeOH or by silica gel column chromatography (Table 2). Triazole chalcones **8a–j** were synthesized by 1,3-dipolar cycloaddition catalyzed by CuI [25] in DMSO using azidochalcones **7a–e** and either propargyl alcohol or phenyl acetylene. The desired products were obtained in excellent yields after silica gel filtration or recrystallization from MeOH (Table 3). All compounds were characterized on basis of extensive analyses of ^1^H, ^13^C{^1^H} and DEPT-135 NMR, HR-MS and IR data.

**Table 2:** *In vitro* cytotoxic activity of chalcone azides **7a–e**.

Compound	R_1_	Yield (%)	IC_50_ (µmol L^−1^)
**7a**	H	50	>100
**7b**	4-OMe	52	>100
**7c**	4-OEt	45	>100
**7d**	4-Cl	81	>100
**7e**	2,3-Cl	68	>100

**Table 3:** *In vitro* cytotoxic activity of triazole chalcones **8a–j**.

Compound	R_1_	R_2_	Yield (%)	IC_50_ (µmol L^−1^)
**8a**	H	CH_2_OH	88	>100
**8b**	4-OMe	CH_2_OH	81	>100
**8c**	4-OEt	CH_2_OH	89	13.03 ± 2.51
**8d**	4-Cl	CH_2_OH	87	>100
**8e**	2,3-Cl	CH_2_OH	91	>100
**8f**	H	Ph	96	>100
**8g**	4-OMe	Ph	92	>100
**8h**	4-OEt	Ph	83	>100
**8i**	4-Cl	Ph	81	>100
**8j**	2,3-Cl	Ph	89	>100
**Cisplatin**	-	-	-	7.37 ± 2.42

The cytotoxic assay demonstrated that the azidochalcones had no effects, with IC_50_ values higher than 100 µM (Table 2). Only one compound (**8c**) exhibited a cytotoxic effect, with an IC_50_ value of 13.03 µM, similar to that of cisplatin (Table 3).

The structural differences among the triazolechalcones are interesting because the modifications in compounds **8b** and **8h** relative to **8c** completely abolish the cytotoxic effect. These data indicate that the size of lateral alkyl group of R_1_ and the chemical nature of the R_2_ substituent may be the keys to developing more cytotoxic compounds.

## 3. Experimental

### 3.1. Chemistry

Reagents and solvents were purchased as reagent grade and used without further purification. All NMR data were acquired in CDCl_3_ or DMSO-*d*_6_ on a Bruker AVANCE 400 NMR spectrometer, operating at 9.4 Tesla, observing ^1^H and ^13^C at 400.13 and 100.61 MHz, respectively. The spectrometer was equipped with a 5-mm multinuclear direct detection probe with *z*-gradient. All ^1^H- and ^13^C-NMR chemical shifts are given in ppm (δ) relative to the TMS signal at 0.00 ppm as internal reference and the coupling constants (*J*) are in Hz. Preparative chromatography was performed using silica gel (230–400 mesh) following the methods described by Still [26]. Thin layer chromatography (TLC) was performed using silica gel GF_254_, 0.25 mm thickness. For visualization, TLC plates were either placed under ultraviolet light, stained with iodine vapor or acidic vanillin. GCMS analysis was performed in a Shimadzu QP-5050A GC-MS gas chromatography-mass spectrometry instrument operated in the electron impact ionization mode (70 eV), and equipped with a DB-5 capillary column (30 m × 0.25 mm i.d. × 0.25 μm; J & W Scientific, Folsom, CA, USA). HPLC analysis was performed in a Shimadzu Prominence liquid chromatography, equipped with a LC-20AT pump, SPD-M20A Photodiode Array detector, SIL-20A automatic injector and a Shim-pack C-18 VP-ODS 4.6 × 250 mm 5 μm reverse phase column. HRMS analysis was recorded on a micrOTOF-QII Bruker. IR spectra were recorded on a Shimadzu IRAffinity-1 spectrophotometer.

### 3.2. General Procedure of O-Alkylation of 1-(2,4-Dihydroxyphenyl)ethan-1-one (resacetophenone, 1) [24,27]

In a round bottom flask were added resacetophenone (**1**, 2.0 g, 13.1 mmol), anhydrous K_2_CO_3_ (5.4 g, 39.1 mmol), 1,4-dibromobutane (5.18 g, 24.2 mmol) and anhydrous acetonitrile (15 mL). After stirring for 5h at 40 °C, 50 mL of water was added and extracted with ethyl acetate (3 × 50 mL). The organic layer was washed with brine, dried over anhydrous Na_2_SO_4_ and concentrated under vacuum. The crude product was purified by silica column chromatography (EtOAc/hex 3:1).

*1-[4-(4-Bromobutoxy)-2-hydroxyphenyl]ethan-1-one* (**3**). White solid 2.26 g (7.9 mmol, 60%); ^1^H-NMR (CDCl_3_) *δ* (ppm): 1.93–2.00 (m, 2H); 2.02–2.09 (m, 2H); 2.56 (s, 3H); 3.48 (t, *J* = 6.4 Hz, 2H); 4.04 (t, *J* = 6.0 Hz, 2H); 6.40 (d, *J* = 2.5 Hz, 1H); 6.43 (dd, *J* = 8.9 Hz, *J* = 2.5 Hz, 1H); 7.63 (d, *J* = 8.9 Hz, 1H); 12.74 (s, 1H); ^13^C-NMR (CDCl_3_) *δ* (ppm): 26.2; 27.6; 29.3; 33.2; 67.2; 101.3; 107.8; 113.9; 132.3; 165.2; 165.3; 202.5. IR (KBr) ν_max_/cm^−1^: 1631.78. HRMS calcd for C_12_H_15_BrO_3_ [M+H^+^]287.0277 found 287.0270; HPLC R_T_: 8.50 min, solvent: MeOH 30%, THF 30%, H_2_O 40%.

*1-[2,4-bis(4-Bromobutoxy)phenyl]ethan-1-one* (**4**). ^1^H-NMR (CDCl_3_) *δ* (ppm): 1.92–2.13 (m, 8H); 2.58 (s, 3H); 3.49 (t, *J* = 6.3 Hz, 2H); 3.50 (t, *J* = 6.3 Hz, 2H); 4.04 (t, *J* = 5.9 Hz, 2H); 4.05 (t, *J* = 5.9 Hz, 2H); 6.42 (d, *J* = 2.2 Hz, 1H); 6.50 (dd, *J* = 8.7 Hz, *J* = 2.2 Hz, 1H); 7.82 (d, *J* = 8.7 Hz, 1H); ^13^C-NMR (CDCl_3_) *δ* (ppm): 27.7; 27.8; 29.3; 29.4; 32.0; 33.1; 33.2; 67.1; 67.4; 99.3; 105.5; 121.2; 132.7; 160.2; 163.7; 197.5. IR (KBr) ν_max_/cm^−1^: 1658.78; 1593.20. MS *m/z* (relative intensity) 422 (M^+^, 6.2), 420 (M^+^, 3.1), 137 (100), 135 (71.1). HPLC R_T_: 12.13 min, solvent: MeOH 30%, THF 30%, H_2_O 40%.

*1-{4-[4-(4-Acetyl-3-hydroxyphenoxy)butoxy]-2-hydroxyphenyl}ethan-1-one* (**5**). ^1^H-NMR (CDCl_3_) *δ* (ppm): 1.96–2.02 (m, 4H); 2.56 (s, 6H); 4.02–4.12 (m, 4H); 6.40 (d, *J* = 2.4 Hz, 2H); 6.43 (dd, *J* = 8.8 Hz, 2.44 Hz, 2H); 7.60 (d, *J* = 8.8 Hz, 2H); 12.74 (s, 2H); ^13^C-NMR (CDCl_3_) *δ* (ppm): 202.55; 165.44; 165.25; 132.34; 113.93; 107.93; 101.35; 67.76; 26.25; 25.72; IR (KBr) ν_max_/cm^−1^: 1633.71; 1614.42. MS *m/z* (relative intensity) 220(24), 205(100), 177(22). ESI-MS (*m/z*): [M+H]^+^ 359.0. HPLC R_T_: 8.96 min, solvent: MeOH 30%, THF 30%, H_2_O 40%.

### 3.3. Preparation of 1-[4-(4-Azidobutoxy)-2-hydroxyphenyl]ethan-1-one (**6**)

In a round bottom flask were added NaN_3_ (0.13 g, 2.0 mmol) and DMSO (4 mL). After total dissolution of the NaN_3_, bromide **3** (0.29 g, 1.0 mmol) was added. After stirring for 3 h at room temperature, water (20 mL) was added and the mixture extracted with ethyl acetate (3 × 30 mL). The organic layer was washed with brine, dried over anhydrous Na_2_SO_4_ and concentrated under vacuum. The crude product was filtered in a small silica gel column to afford a low melting yellow oil. 0.22 g (0.88 mmol, 87%); ^1^H-NMR (CDCl_3_) *δ* (ppm): 1.75–1.83 (m, 2H); 1.85–1.92 (m, 2H); 2.56 (s, 3H); 3.36 (t, *J* = 6.7 Hz, 2H); 4.03 (t, *J* = 6.0 Hz, 2H); 6.40 (d, *J* = 2.5 Hz); 6.44 (dd, *J* = 8.9 Hz, *J* = 2.5 Hz, 1H); 7.63 (d, *J* = 8.9 Hz, 1H); 12.74 (s, 1H). ^13^C-NMR (CDCl_3_) *δ* (ppm): 25.6; 26.2; 26.24; 51.1; 67.5; 101.3; 107.9; 113.9; 132.3; 165.2; 165.3; 202.5. MS *m/z* (relative intensity) 249 (M^+^, 3.5), 204 (40), 137 (100). IR (KBr) ν_max_/cm^−1^: 1651.07; 2096.62. HPLC R_T_: 8.98 min, solvent MeOH 70% : H_2_O 30%.

### 3.4. General Procedure for the Synthesis and Purification of Chalcones

In a round bottom flask were added compound **4** (0.25 g, 1.0 mmol), the appropriate benzaldehyde (1.2 mmol) and methanol (2 mL). To this solution was added dropwise a solution of KOH in methanol (2 mL, 5 mol·L^−1^). After stirring for 48 h at room temperature, water (20 mL) was added and the mixture extracted with ethyl acetate (3 × 30 mL). The organic layer was washed with brine, dried over anhydrous Na_2_SO_4_ and concentrated under vacuum. 

*(2E)-1-[4-(4-Azidobutoxy)-2-hydroxyphenyl]-3-phenylprop-2-en-1-one* (**7a**). Recrystallized from MeOH, yellow solid, yield 0.17 g (0.50 mmol, 50%); ^1^H-NMR (CDCl_3_) *δ* (ppm): 1.81 (*m*, 2H); 1.90 (*m*, 2H); 3.38 (*t*, *J* = 6.5 Hz, 2H); 4.06 (*t*, *J* = 6.0 Hz, 2H); 6.46 (d, *J* = 2.5 Hz, 1H); 6.49 (dd, *J* = 8.9 and 2.5 Hz, 1H); 7.43 (*m*, 1H); 7.44 (*m*, 2H); 7.59 (d, *J* = 15.5 Hz, 1H); 7.66 (*m*, 2H); 7.84 (d, *J* = 8.9 Hz, 1H); 7.90 (d, *J* = 15.5 Hz, 1H); 13.44 (*brs*, 1H); ^13^C-NMR (CDCl_3_) *δ* (ppm): 25.7; 26.3; 51.1; 67.6; 101.5; 108.0; 114.1; 120.3; 128.5; 129.0; 130.7; 131.3; 134.8; 144.4; 165.5; 166.7; 191.8; IR (KBr) ν_max_/cm^−1^: 1635.64; 1570.06; 2088.91; HRMS (*m/z*): [M+Na]^+^ 360.1312 (C_19_H_19_N_3_NaO_3_^+^ = 360.1319)

*(2E)-1-[4-(4-Azidobutoxy)-2-hydroxyphenyl]-3-(4-methoxyphenyl)prop-2-en-1-one* (**7b**). Recrystallized from MeOH, yellow solid, yield 0.19 g (0.52 mmol, 52%); ^1^H-NMR (CDCl_3_) *δ* (ppm): 1.80 (*m*, 2H); 1.90 (*m*, 2H); 3.38 (*t*, *J* = 6.6 Hz, 2H); 3.86 (*s*, 3H); 4.04 (*t*, 6.1 Hz, 2H); 6.44 (*d*, *J* = 2.5 Hz, 1H); 6.47 (*dd*, *J* = 8.9 and 2.5 Hz, 1H); 6.94 (*d*, *J* = 8.8 Hz, 2H); 7.46 (*d*, *J* = 15.4 Hz, 1H); 7.62 (*d*, *J* = 8.8 Hz, 2H); 7.83 (*d*, *J* = 8.9 Hz, 1H); 7.86 (*d*, *J* = 15.4 Hz, 1H); 13.56 (*brs*, 1H); ^13^C-NMR (CDCl_3_) *δ* (ppm): 25.7; 26.3; 51.1; 55.4; 67.5; 101.6; 107.9; 114.2; 114.5; 117.8; 127.6; 130.4; 131.2; 144.4; 161.8; 165.3; 166.5; 191.8; IR (KBr) ν_max_/cm^−1^: 1637.56; 1568.13; 2088.91; HRMS (*m/z*): [M+Na]^+^ 390,1420 (C_20_H_21_N_3_NaO_4_^+^ = 390,1424)

*(2E)-1-[4-(4-Azidobutoxy)-2-hydroxyphenyl]-3-(4-ethoxyphenyl)prop-2-en-1-one* (**7c**). Recrystallized from MeOH, yellow solid, yield 0.17 g (0.45 mmol, 45%); ^1^H-NMR (CDCl_3_) *δ* (ppm): 1.44 (*t*, *J* = 7.0 Hz, 1H); 1.80 (*m*, 2H); 1.89 (*m*, 2H); 3.38 (*t*, *J* = 6.7 Hz, 2H); 4.04 (*t*, *J* = 6.0 Hz, 2H); 4.08 (*q*, *J* = 7.0 Hz, 2H); 6.44 (*d*, *J* = 2.5 Hz, 1H); 6.47 (*dd*, *J* = 8.9 and 2.5 Hz, 1H); 6.93 (*d*, *J* = 8.8 Hz, 2H); 7.45 (*d*, *J* = 15.4 Hz, 1H); 7.60 (*d*, *J* = 8.8 Hz, 2H); 7.82 (*d*, *J* = 8.9 Hz, 1H); 7.86 (*d*, *J* = 15.4 Hz, 1H); 13.57 (*brs*, 1H); ^13^C-NMR (CDCl_3_) *δ* (ppm): 14.7; 25.7; 26.3; 51.1; 63.7; 67.5; 101.5; 107.8; 114.2; 114.9; 117.6; 127.3; 130.4; 131.2; 144.4; 161.2; 165.3; 166.6; 191.9; IR (KBr) ν_max_/cm^−1^: 1633.71; 1567.27; 2090.84; HRMS (*m/z*): [M+Na]^+^ 404.1586 (C_21_H_23_N_3_NaO_4_^+^ = 404,1581).

*(2E)-1-[4-(4-Azidobutoxy)-2-hydroxyphenyl]-3-(4-chlorophenyl)prop-2-en-1-one* (**7d**). Purification by column chromatography, hexane/ethyl acetate (7:1), yellow solid, 0.30 g (0.81 mmol, 81%); ^1^H-NMR (CDCl_3_) *δ* (ppm): 1.80 (*m*, 2H); 1.89 (*m*, 2H); 3.38 (*t*, *J* = 6.7 Hz, 2H); 4.04 (*t*, *J* = 5.9 Hz, 2H); 6.45 (*d*, *J* = 2.5 Hz, 1H); 6.48 (*dd*, *J* = 8.9 and 2.5 Hz, 1H); 7.40 (*d*, *J* = 8.5 Hz, 2H); 7.54 (*d*, *J* = 15.5 Hz, 1H); 7.58 (*d*, *J* = 8.5 Hz, 2H); 7.80 (*d*, *J* = 8.9 Hz, 1H); 7.82 (*d*, *J* = 15.5 Hz, 1H), 13.38 (*brs*, 1H); ^13^C-NMR (CDCl_3_) *δ* (ppm): 25.7; 26.3; 51.1; 67.6; 101.6; 108.1; 114.1; 120.8; 129.3; 129.7; 131.2; 133.3; 136.6; 142.9; 165.6; 166.7; 191.5; IR (KBr) ν_max_/cm^−1^: 1641.42; 1573.91; 2086.98; HRMS (*m/z*): [M+H]^+^ 372.135 (C_19_H_19_ClN_3_O_3_^+^ = 372.111)

*(2E)-1-[4-(4-Azidobutoxy)-2-hydroxyphenyl]-3-(2,3-dichlorophenyl)prop-2-en-1-one* (**7e**). Purification by column chromatography, hexane/ethyl acetate (3:1), yellow solid, 0.28 g (0.69 mmol, 68%); ^1^H-NMR (CDCl_3_) *δ* (ppm): 1.80 (*m*, 2H); 1.90 (*m*, 2H); 3.38 (*t*, *J* = 6.7 Hz, 2H); 4.05 (*t*, *J* = 6.0 Hz, 2H); 6.45 (*d*, *J* = 2.5 Hz, 1H); 6.48 (*dd*, *J* = 8.9 and 2.5 Hz, 1H); 7.27 (*dd*, *J* = 8.0 and 7.7 Hz, 1H); 7.51 (*dd*, *J* = 8.0 and 1.4 Hz, 1H); 7.52 (*d*, *J* = 15.5 Hz, 1H); 7.64 (*dd*, *J* = 7.7 and 1.4 Hz, 1H), 7.79 (*d*, *J* = 8.9 Hz, 1H); 8.24 (*d*, *J* = 15.5 Hz, 1H); 13.26 (*brs*, 1H). ^13^C-NMR (CDCl_3_) *δ* (ppm): 25.7; 26.3; 51.1; 67.7; 101.6; 108.3; 114.0; 124.3; 126.0; 127.4; 131.4; 131.8; 132.4; 134.2; 135.6; 140.1; 165.8; 166.9; 191.3; IR (KBr) ν_max_/cm^−1^: 1637.56; 1575.84; 2090.84; HRMS (*m/z*): [M+Na]^+^ 428.0538 (C_19_H_17_Cl_2_N_3_NaO_3_^+^ = 428.0539).

### 3.5. General Cycloaddition Procedure

In a round bottom flask were added the appropriate azidechalcone (1.0 mmol), propargyl alcohol (0.23 mL, 4.0 mmol) or phenylacetylene (0.44 mL, 4.0 mmol), dimethylsulfoxide (4 mL) and a catalytic amount of CuI. After stirring for 2 h at room temperature, water (10 mL) was added and the mixture acidified with HCl (1.2 mol·L^−1^). The crude residue was extracted with ethyl acetate (3 × 30 mL) and organic layer was washed with brine, dried over anhydrous Na_2_SO_4_ and concentrated under vacuum. The reside was filtered in a little silica column and recrystallized from MeOH.

*(2E)-1-(2-Hydroxy-4-{4-[4-(hydroxymethyl)-1H-1,2,3-triazol-1-yl]butoxy}phenyl)-3-phenylprop-2-en-1-one* (**8a**). Yellow solid, 0.35 g (0.89 mmol, 88%); ^1^H-NMR (DMSO-*d_6_*) *δ* (ppm): 1.70 (*m*, 2H); 1.98 (*m*, 2H); 4.10 (*t*, *J* = 6.4 Hz, 2H); 4.43 (*t*, *J* = 7.0 Hz, 2H); 4.52 (*d*, *J* = 5.4 Hz, 2H); 5.19 (*t*, *J* = 5.4 Hz, 1H); 6.52 (*d*, *J* = 2.5 Hz, 1H); 6.57 (*dd*, *J* = 9.0 and 2.5 Hz, 1H); 7.48 (*m*, 3H); 7.84 (*d*, *J* = 15.5 Hz, 1H); 7.92 (*m*, 2H); 8.00 (*s*, 1H); 8.03 (*d*, *J* = 15.5 Hz, 1H); 8.29 (*d*, *J* = 9.0 Hz, 1H); 13.45 (*brs*, 1H); ^13^C-NMR (DMSO-*d_6_*) *δ* (ppm): 25.5; 26.5; 48.8; 55.1; 67.5; 101.4; 107.8; 113.8; 121.2; 122.7; 128.9; 129.1; 130.8; 132.8; 134.5; 144.2; 148.0; 165.3; 165.7; 191.9; IR (KBr) ν_max_/cm^−1^: 3383.14; 1631.78; 1568.13; HRMS (*m/z*): [M+H]^+^ 394.1758 (C_22_H_24_N_3_O_4_^+^ = 394.1761)

*(2E)-1-(2-Hydroxy-4-{4-[4-(hydroxymethyl)-1H-1,2,3-triazol-1-yl]butoxy}phenyl)-3-(4-methoxy-phenyl)prop-2-en-1-one* (**8b**). Yellow solid, 0.35 g (0.83 mmol, 81%); ^1^H-NMR (DMSO-*d_6_* and CDCl_3_) *δ* (ppm): 1.81 (*m*, 2H); 2.09 (*m*, 2H); 4.07 (*t*, *J* = 6.2 Hz, 2H); 3.86 (*s*, 3H); 4.45 (*t*, *J* = 7.0 Hz, 2H); 4.63 (*d*, *J* = 4.7 Hz, 2H); 5.10 (*t*, *J* = 4.7 Hz, 1H); 6.42 (*d*, *J* = 2.5 Hz, 1H); 6.49 (*dd*, *J* = 9.0 and 2.5 Hz, 1H); 6.97 (*d*, *J* = 8.8 Hz, 2H); 7.65 (*d*, *J* = 15.4 Hz, 1H); 7.73 (*d*, *J* = 8.8 Hz, 2H); 7.812 (*d*, *J* = 15.4 Hz, 1H); 7.808 (*s*, 1H); 8.04 (*d*, *J* = 9.0 Hz, 1H); 13.54 (*brs*, 1H); ^13^C-NMR (DMSO-*d_6_* andCDCl_3_) *δ* (ppm): 25.3; 26.4; 48.9; 54.9; 55.3; 66.9; 101.0; 107.1; 113.6; 114.0; 117.6; 121.8; 126.9; 130.3; 131.5; 143.8; 148.0; 161.3; 164.7; 165.7; 191.3; IR (KBr) ν_max_/cm^−1^: 3292.49; 1635.64; 1577.77; HRMS (*m/z*): [M+H]^+^ 424.1869 (C_23_H_26_N_3_O_5_^+^ = 424.1867).

*(2E)-3-(4-Ethoxyphenyl)-1-(2-hydroxy-4-{4-[4-(hydroxymethyl)-1H-1,2,3-triazol-1-yl]butoxy}phenyl)-prop-2-en-1-one* (**8c**). Yellow solid, 0.39 g (0.89 mmol, 89%); ^1^H-NMR (CDCl_3_) *δ* (ppm): 1.44 (*t*, *J* = 7.0 Hz, 3H); 1.83 (*m*, 2H); 2.12 (*m*, 2H); 4.02 (*t*, *J* = 6.0 Hz, 2H); 4.08 (*q*, *J* = 7.0 Hz, 2H); 4.44 (*t*, *J* = 7.1 Hz, 2H); 4.80 (*s*, 2H); 6.41 (*d*, *J* = 2.4 Hz, 1H); 6.44 (*dd*, *J* = 8.9 and 2.4 Hz, 1H); 6.92 (*d*, *J* = 8.6 Hz, 2H); 7.44 (*d*, *J* = 15.4 Hz, 1H); 7.58 (*s*, 1H); 7.60 (*d*, *J* = 8.6 Hz, 2H); 7.82 (*d*, *J* = 8.9 Hz, 1H); 7.85 (*d*, *J* = 15.4 Hz, 1H); 13.56 (*brs*, 1H); ^13^C-NMR (CDCl_3_) *δ* (ppm): 14.7; 26.0; 27.1; 50.0; 56.5; 63.7; 67.3; 101.6; 107.7; 114.3; 114.9; 117.6; 121.7; 127.3; 130.4; 131.2; 144.5; 147.9; 161.3; 165.1; 166.5; 191.9; IR (KBr) ν_max_/cm^−1^: 3278.99; 1639.49; 1568.13; HRMS (*m/z*): [M+Na]^+^ 460.1838 (C_24_H_27_N_3_NaO_5_^+^ = 460.1843).

*(2E)-3-(4-Chlorophenyl)-1-(2-hydroxy-4-{4-[4-(hydroxymethyl)-1H-1,2,3-triazol-1-yl]butoxy}phenyl)-prop-2-en-1-one* (**8d**). Yellow solid, 0.37 g (0.87 mmol, 87%); ^1^H-NMR (DMSO-*d_6_*) *δ* (ppm): 1.71 (*m*, 2H); 1.98 (*m*, 2H); 4.11 (*t*, *J* = 6.4 Hz, 2H); 4.42 (*t*, *J* = 7.1 Hz, 2H); 4.52 (*d*, *J* = 4.7 Hz, 2H); 5.16 (*t*, *J* = 4.7 Hz, 1H); 6.52 (*d*, *J* = 2.5 Hz, 1H); 6.57 (*dd*, *J* = 9.1 and 2.5 Hz, 1H); 7.54 (*d*, *J* = 8.6 Hz, 1H); 7.81 (*d*, *J* = 15.5 Hz, 1H); 7.96 (*d*, *J* = 8.6 Hz, 2H); 8.00 (*s*, 1H); 8.04 (*d*, *J* = 15.5 Hz, 1H); 8.28 (*d*, *J* = 9.1 Hz, 1H); 13.38 (*brs*, 1H); ^13^C-NMR (DMSO-*d_6_*) *δ* (ppm): 25.4; 26.4; 48.7; 55.0; 67.4; 101.3; 107.7; 113.8; 122.0; 122.6; 128.9; 130.7; 132.8; 133.5; 135.2; 142.5; 148.0; 165.3; 165.7; 191.6; IR (KBr) ν_max_/cm^−1^: 3367.71; 1643.35; 1577.77; HRMS (*m/z*): [M+Na]^+^ 450.1195 (C_22_H_22_ClN_3_NaO_4_^+^ = 450.1191).

*(2E)-3-(2,3-Dichlorophenyl)-1-(2-hydroxy-4-{4-[4-(hydroxymethyl)-1H-1,2,3-triazol-1-yl]butoxy}-phenyl)prop-2-en-1-one* (**8e**). Yellow solid, 0.43 g (0.93 mmol, 91%); ^1^H-NMR (DMSO-*d_6_*) *δ* (ppm): 1.71 (*m*, 2H); 1.97 (*m*, 2H); 4.11 (*t*, *J* = 6.4 Hz, 2H); 4.43 (*t*, *J* = 7.0 Hz, 2H); 4.52 (*s*, 2H); 5.17 (*s*, 1H); 6.53 (*d*, *J* = 2.5 Hz, 1H); 6.57 (*dd*, *J* = 9.0 and 2.5 Hz, 1H); 7.48 (*dd*, *J* = 8.0 and 8.1 Hz, 1H); 7.74 (*dd*, *J* = 8.0 and 1.3 Hz, 1H); 8.00 (*s*, 1H); 8.07 (*d*, *J* = 15.5 Hz, 1H); 8.11 (*d*, *J* = 15.5 Hz, 1H); 8.21 (*dd*, *J* = 8.1 and 1.3 Hz, 1H); 8.26 (*d*, *J* = 9.0 Hz, 1H); 13.17 (*brs*, 1H); ^13^C-NMR (DMSO-*d_6_*) *δ* (ppm): 25.4; 26.4; 48.8; 55.0; 67.5; 101.4; 107.9; 113.8; 122.6; 125.5; 127.3; 128.3; 132.00; 132.04; 132.5; 132.9; 134.7; 138.2; 148.0; 165.5; 165.6; 191.1; IR (KBr) ν_max_/cm^−1^: 3414.00; 1633.71; 1571.99; HRMS (*m/z*): [M+Na]^+^ 484.0801 (C_22_H_21_Cl_2_N_3_NaO_4_^+^ = 484.0801).

*(2E)-1-{2-Hydroxy-4-[4-(4-phenyl-1H-1,2,3-triazol-1-yl)butoxy]phenyl}-3-phenylprop-2-en-1-one* (**8f**). Yellow solid, 0.42 g (0.96 mmol, 96%); ^1^H-NMR (CDCl_3_) *δ* (ppm): 1.89 (*m*, 2H); 2.19 (*m*, 2H); 4.06 (*t*, *J* = 6.0 Hz, 2H); 4.51 (*t*, *J* = 7.0 Hz, 2H); 6.44 (*d*, *J* = 2.5 Hz, 1H); 6.46 (*dd*, *J* = 8.6 and 2.5 Hz, 1H) 7.33 (*tt*, *J* = 7.4 and 1.3 Hz, 1H); 7.43 (*m*, 5H); 7.57 (*d*, *J* = 15.5 Hz, 1H); 7.66 (*m*, 2H); 7.78 (*s*, 1H); 7.82 (*d*, *J* = 8.6 Hz, 1H); 7.83 (*m*, 2H); 7.89 (*d*, *J* = 15.5 Hz, 1H); 13.4 (*brs*, 1H); ^13^C-NMR (CDCl_3_) *δ* (ppm): 26.0; 27.2; 50.0; 67.3; 101.8; 107.8; 114.3; 119.5; 120.4; 125.7; 128.2; 128.6; 128.9; 129.0; 130.6; 130.8; 131.4; 134.8; 144.5; 148.0; 165.3; 166.6; 191.9; IR (KBr) ν_max_/cm^−1^: 3419.79; 1639.49; 1573.91; HRMS (*m/z*): [M+H]^+^ 440.248 (C_27_H_26_N_3_O_3_^+^ = 440.197).

*(2E)-1-{2-Hydroxy-4-[4-(4-phenyl-1H-1,2,3-triazol-1-yl)butoxy]phenyl}-3-(4-methoxyphenyl)prop-2-en-1-one* (**8g**). Yellow solid, 0.43 g (0.92 mmol, 92%); ^1^H-NMR (CDCl_3_) *δ* (ppm): 1.89 (*m*, 2H); 2.19 (*m*, 2H); 3.87 (*s*, 3H); 4.06 (*t*, *J* = 6.0 Hz, 2H); 4.51 (*t*, *J* = 7.0 Hz, 2H); 6.44 (*d*, *J* = 2.5 Hz, 1H); 6.45 (*dd*, *J* = 8.5 and 2.5 Hz, 1H); 6.95 (*d*, *J* = 8.8 Hz, 2H); 7.33 (*tt*, *J* = 7.5 and 1.3 Hz, 1H); 7.42 (*m*, 2H); 7.44 (*d*, *J* = 15.4 Hz, 1H); 7.62 (*d*, *J* = 8.8 Hz, 2H); 7.78 (*s*, 1H); 7.82 (*m*, 2H); 7.83 (*d*, *J* = 8.5 Hz, 1H); 7.86 (*d*, *J* = 15.4 Hz, 1H); 13.52 (*brs*, 1H); ^13^C-NMR (CDCl_3_) *δ* (ppm): 26.0; 27.2; 50.0; 55.5; 67.3; 101.7; 107.7; 114.3; 114.5; 117.9; 119.5; 125.7; 127.6; 128.2; 128.9; 130.4; 130.6; 131.2; 144.4; 148.0; 161.9; 165.1; 166.6; 191.9; IR (KBr) ν_max_/cm^−1^: 3446.79; 1633.71; 1570.06; HRMS (*m/z*): [M+Na]^+ ^492.1893 (C_28_H_27_N_3_NaO_4_^+^ = 492.1894).

*(2E)-3-(4-Ethoxyphenyl)-1-{2-hydroxy-4-[4-(4-phenyl-1H-1,2,3-triazol-1-yl)butoxy]phenyl}prop-2-en-1-one* (**8h**). Yellow solid, 0.40 g (0.83 mmol, 83%); ^1^H-NMR (DMSO-*d*_6_) *δ* (ppm): 1.34 (*t*, *J* = 7.0 Hz, 3H); 1.75 (*m*, 2H); 2.04 (*m*, 2H); 4.10 (*q*, *J* = 7.0 Hz, 2H); 4.11 (*t*, *J* = 6.2 Hz, 2H); 4.49 (*t*, *J* = 7.0 Hz, 2H); 6.50 (*d*, *J* = 2.5 Hz, 1H); 6.55 (*dd*, *J* = 9.0 and 2.5 Hz, 1H); 7.00 (*d*, *J* = 8.8 Hz, 2H); 7.32 (*tt*, *J* = 7.4 and 1.3 Hz, 1H); 7.44 (*m*, 2H); 7.84 (*m*, 2H); 7.87 (*d*, *J* = 8.8 Hz, 2H); 7.80 (*d*, *J* = 15.5 Hz, 1H); 7.87 (*d*, *J* = 15.5 Hz, 1H); 8.26 (*d*, *J* = 9.0 Hz, 1H); 8.61 (*s*, 1H); 13.62 (*brs*, 1H); ^13^C-NMR (DMSO-*d*_6_) *δ* (ppm): 14.5; 25.5; 26.3; 49.2; 63.4; 67.4; 101.4; 107.6; 113.8; 114.8; 118.3; 121.3; 125.1; 127.1; 127.8; 128.9; 130.8; 131.1; 132.5; 144.3; 146.3; 160.9; 165.1; 165.7; 191.8; IR (KBr) ν_max_/cm^−1^: 3481.51; 1635.64; 1571.99; HRMS (*m/z*): [M+H]^+^ 484.237 (C_29_H_29_N_3_NaO_4_^+^ = 484.223).

*(2E)-3-(4-Chlorophenyl)-1-{2-hydroxy-4-[4-(4-phenyl-1H-1,2,3-triazol-1-yl)butoxy]phenyl}prop-2-en-1-one* (**8i**). Yellow solid, 0.38 g (0.80 mmol, 81%); ^1^H-NMR (DMSO-*d*_6_) *δ* (ppm): 1.76 (*m*, 2H); 2.05 (*m*, 2H); 4.13 (*t*, *J* = 6.4 Hz, 2H); 4.49 (*t*, *J* = 7.0 Hz, 2H); 6.52 (*d*, *J* = 2.5 Hz, 1H); 6.56 (*dd*, *J* = 9.0 and 2.5 Hz, 1H); 7.33 (*tt*, *J* = 7.4 and 1.3 Hz, 1H); 7.44 (*m*, 2H); 7.53 (*d*, *J* = 8.5 Hz, 2H); 7.78 (*s*, 1H); 7.80 (*d*, *J* = 15.6 Hz, 1H); 7.84 (*m*, 2H); 7.95 (*d*, *J* = 8.5 Hz, 2H); 8.02 (*d*, *J* = 15.5 Hz, 1H); 8.26 (*d*, *J* = 9.0 Hz, 1H); 13.39 (*brs*, 1H); ^13^C-NMR (DMSO-*d*_6_) *δ* (ppm): 25.4; 26.2; 49.1; 67.4; 101.3; 107.7; 113.8; 121.3; 122.0; 125.0; 127.7; 128.8; 128.9; 130.69; 130.73; 132.7; 133.5; 135.2; 142.5; 146.2; 165.3; 165.6; 191.6; IR (KBr) ν_max_/cm^−1^: 3446.79; 1635.64; 1577.77; HRMS (*m/z*): [M+H]^+^ 474.1579 (C_27_H_24_ClN_3_NaO_3_^+^ = 474.1579).

*(2E)-3-(2,3-Dichlorophenyl)-1-{2-hydroxy-4-[4-(4-phenyl-1H-1,2,3-triazol-1-yl)butoxy]phenyl}prop-2-en-1-one* (**8j**). Yellow solid, 0.45 g (0.89 mmol, 89%); ^1^H-NMR (CDCl_3_) *δ* (ppm): 1.89 (*m*, 2H); 2.19 (*m*, 2H); 4.06 (*t*, *J* = 6.0 Hz, 2H); 4.51 (*t*, *J* = 7.0 Hz, 2H); 6.45 (*d*, *J* = 2.5 Hz, 1H); 6.46 (*dd*, *J* = 8.6 and 2.5 Hz, 1H); 7.27 (*dd*, *J* = 8.0 and 7.9 Hz, 1H); 7.33 (*tt*, *J* = 7.4 and 1.3 Hz, 1H); 7.43 (*m*, 2H); 7.52 (*dd*, *J* = 8.0 and 1.3 Hz, 1H); 7.64 (*dd*, *J* = 7.9 and 1.3 Hz, 1H); 7.51 (*d*, *J* = 15.4 Hz, 1H); 7.78 (*s*, 1H); 7.82 (*m*, 2H); 7.83 (*d*, *J* = 8.4 Hz, 1H); 8.24 (*d*, *J* = 15.4 Hz, 1H); 13.24 (*brs*, 1H); ^13^C-NMR (CDCl_3_) *δ* (ppm): 26.0; 27.1; 50.0; 67.3; 101.7; 108.0; 114.1; 119.4; 124.3; 125.8; 126.0; 127.4; 128.2; 128.9; 130.6; 131.5; 131.8; 133.6; 134.6; 135.6; 140.2; 148.0; 165.6; 166.8; 191.3. IR (KBr) ν_max_/cm^−^^1^: 3446.79; 1635.64; 1577.77; HRMS (*m/z*): [M+Na]^+^ 530.1006 (C_27_H_23_Cl_2_N_3_NaO_3_^+^ = 530.1009).

### 3.6. Biological Assays

#### Cytotoxic Assay

The cytotoxicity of the chalcones was assessed with the human cell line HeLa, using the 3-(4,5-dimethylthiazol-2-yl)-2,5-diphenyltetrazolium bromide (MTT, Sigma, St. Louis, MO, USA) colorimetric method. Briefly, the cells were plated in 96-well plates (1 × 10^5^ cells/well) and incubated for 24 h at 37 °C in a humid atmosphere with 5% CO_2_, to adhesion. After this period, the wells were washed with culture medium (EMEM + 10% inactivated fetal calf serum + 2 mM L-glutamine) and incubated with the chalcones at different concentrations (0.01 µM to 500 µM). Control with cisplatin (Sigma-Aldrich, St. Louis, MO, USA), used as reference anticancer drug, was performed in parallel. After the incubation, the plates were treated with MTT. The reading was performed using a SpectraMax M5e microplate reader (Molecular Devices, Sunnyvale, CA, USA) at 550 nm. Cytotoxicity was scored as the percentage reduction in absorbance versus untreated control cultures. All experiments were performed in triplicate. The results were expressed as the mean of the IC_50_ (the lethal drug concentration that reduced cell viability by 50%).

## 4. Conclusions

In summary, we have developed an efficient synthesis of biologically interesting chalcones substituted with azide or triazole groups. These compounds were evaluated *in vitro* against the HeLa cell line and exhibited no cytotoxic activity, with IC_50_ values higher than 100 µM. Only compound **8c** had an IC_50_ value similar to that of cisplatin, an antitumor agent. This initial study of the high yield synthesis of new chalcones substituted with azide or triazole groups, and tested against the HeLa cell line, is a promising work to the discovery of a series of compounds for further optimization aiming for more potent cytotoxic action with lower IC_50_ values.

## Supplementary Materials

Supplementary materials can be accessed at: http://www.mdpi.com/1420-3049/17/9/10331/s1.
