# Supplementary material for: Design and Synthesis of New Chacones Substituted with Azide/Triazole Groups and Analysis of Their Cytotoxicity Towards HeLa Cells
Source: Molecules. 2012 Aug 29;17(9):10331–43. doi: 10.3390/molecules170910331 (PMC6268421; doi:10.3390/molecules170910331)

Figure S1. 1H-NMR spectrum of compound **3** ($CDCl_3$, 400 MHz).

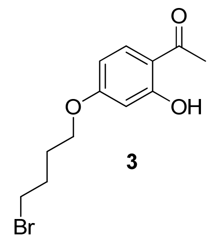

3

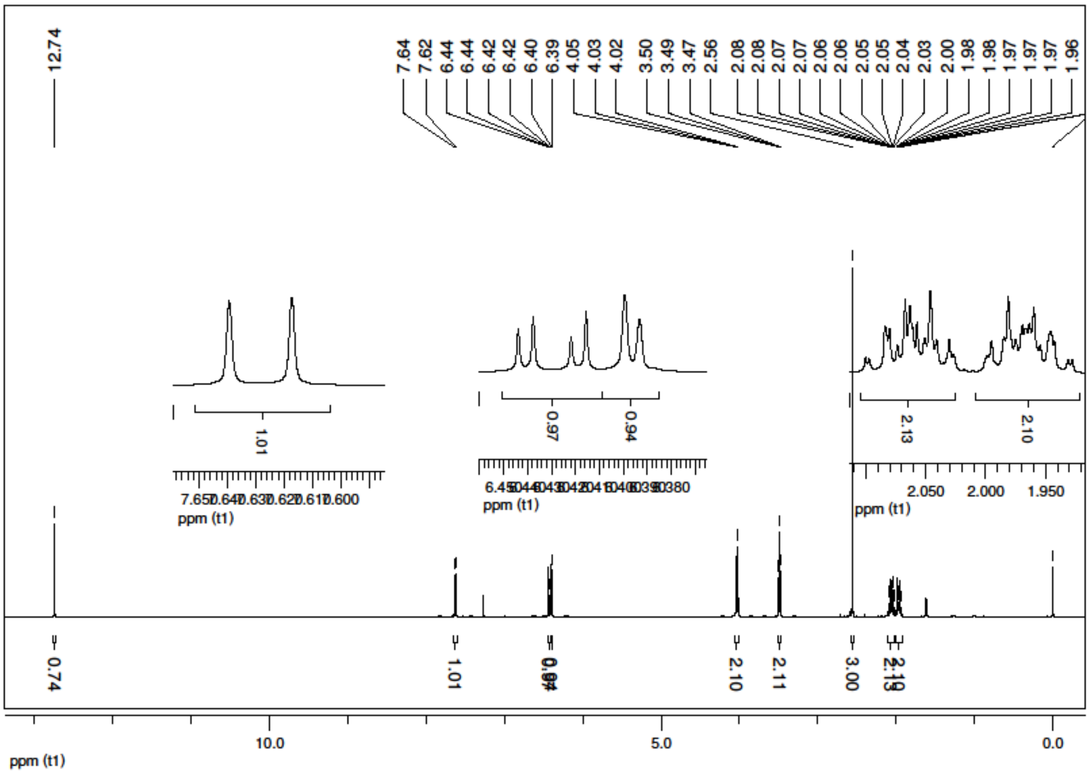

Figure S2. ^{13}C-NMR spectrum of compound **3** ($CDCl_3$, 100 MHz).

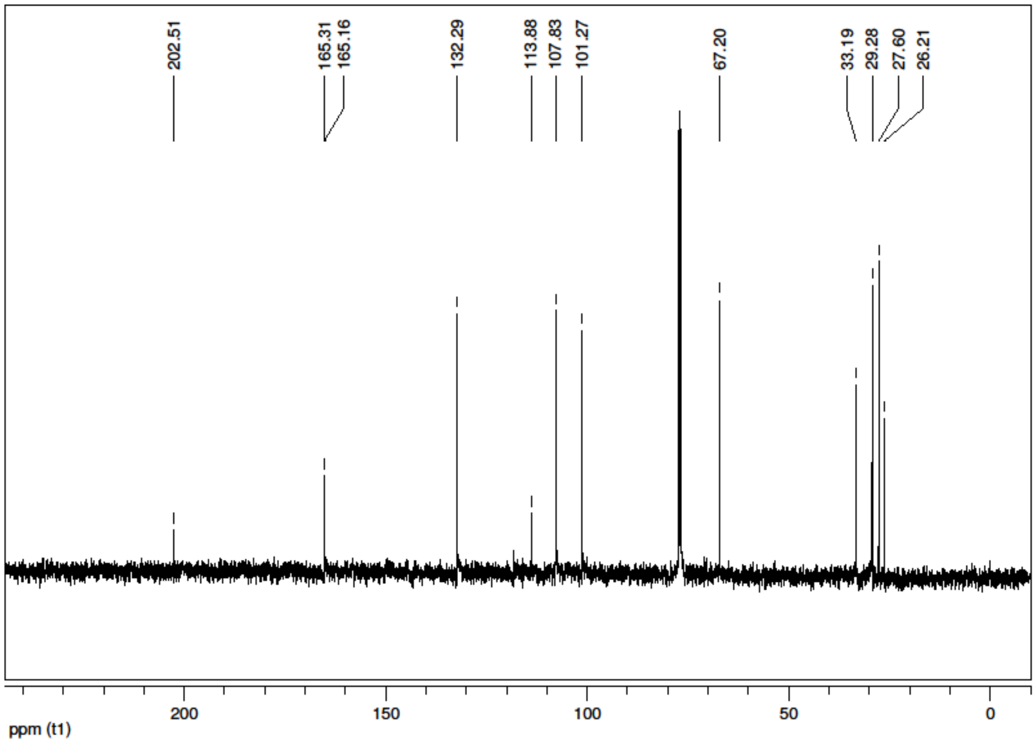

Figure S3. IR spectrum of compound 3.

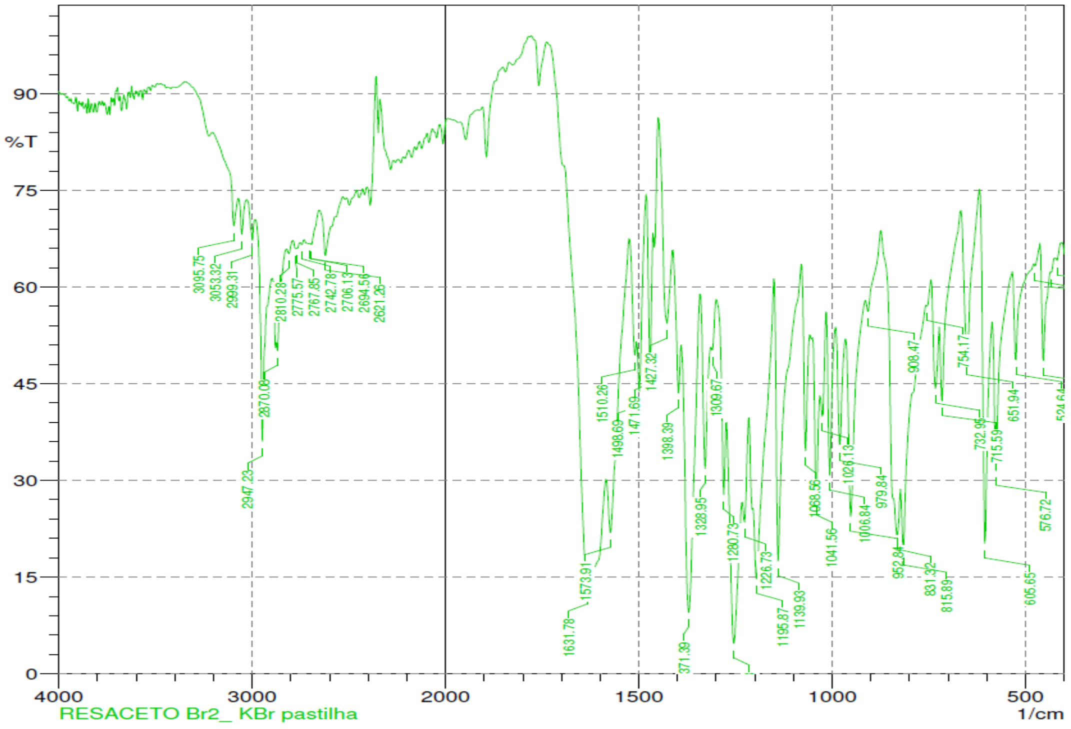

Figure S4. HRMS spectrum of compound 3.

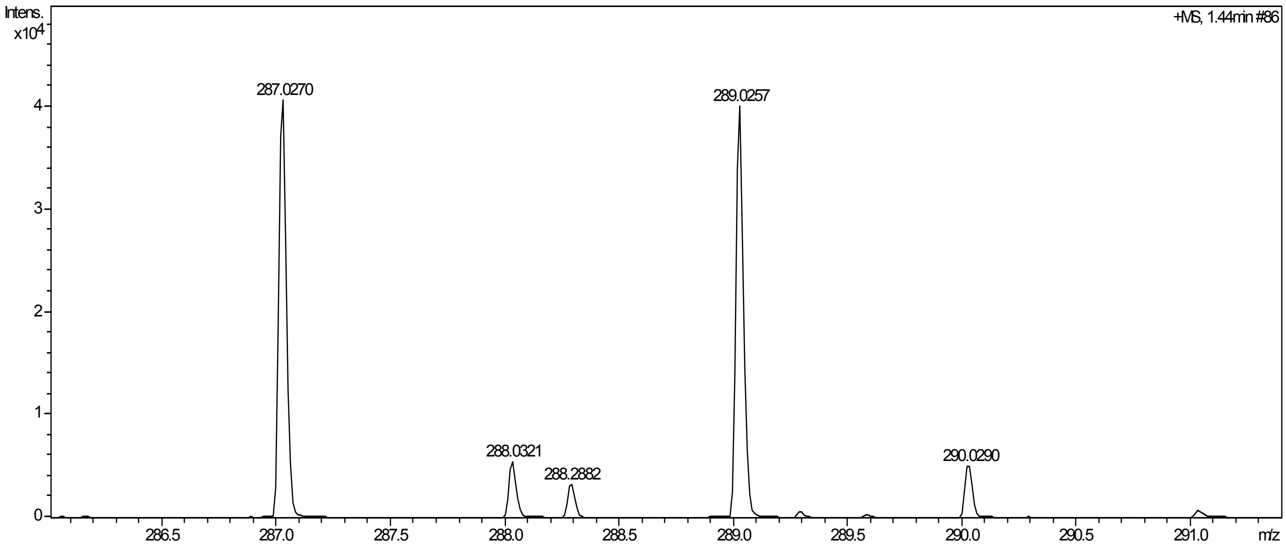

Figure S5. 1H-NMR spectrum of compound **4** ($CDCl_3$, 400 MHz).

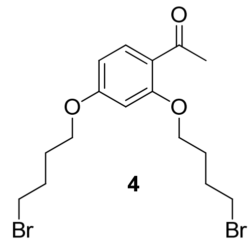

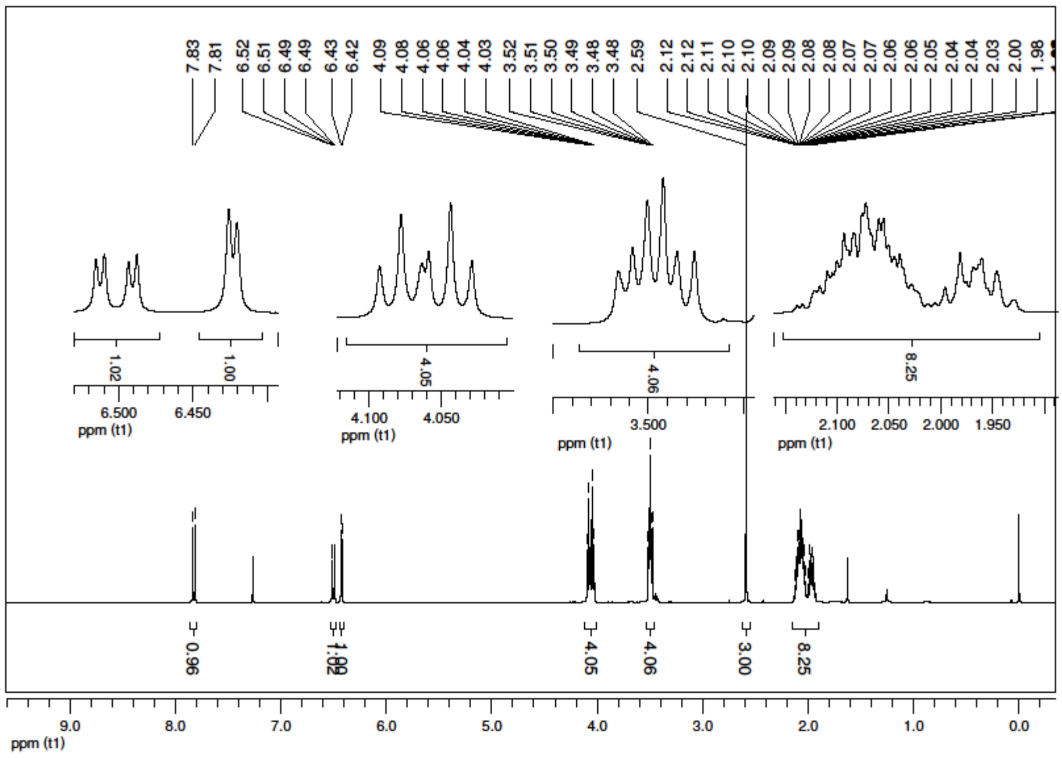

Figure S6. ^{13}C-NMR spectrum of compound **4** ($CDCl_3$, 100 MHz).

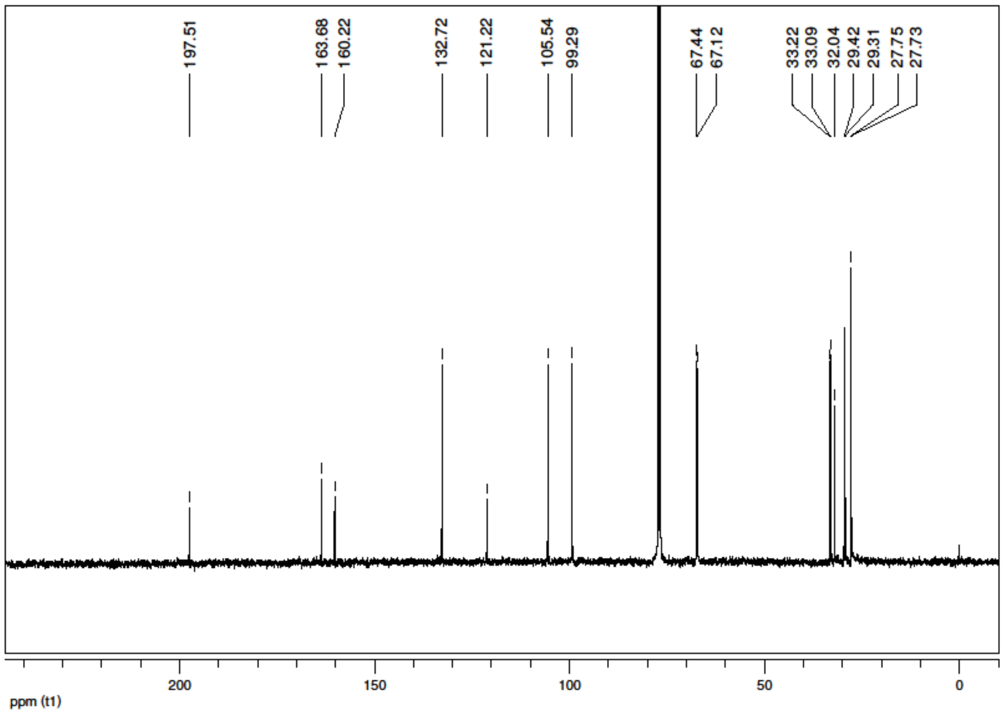

Figure S7. IR spectrum of compound **4**.

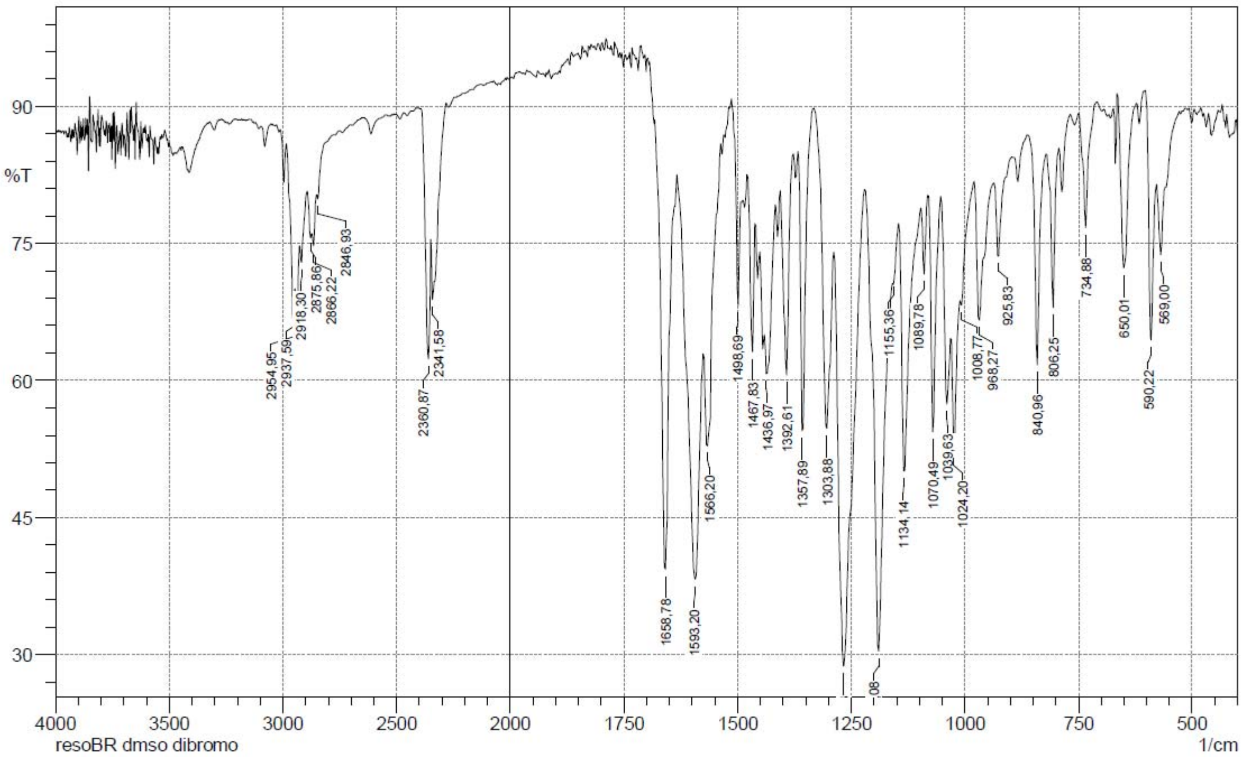

Figure S8. Mass spectrum of compound **4**.

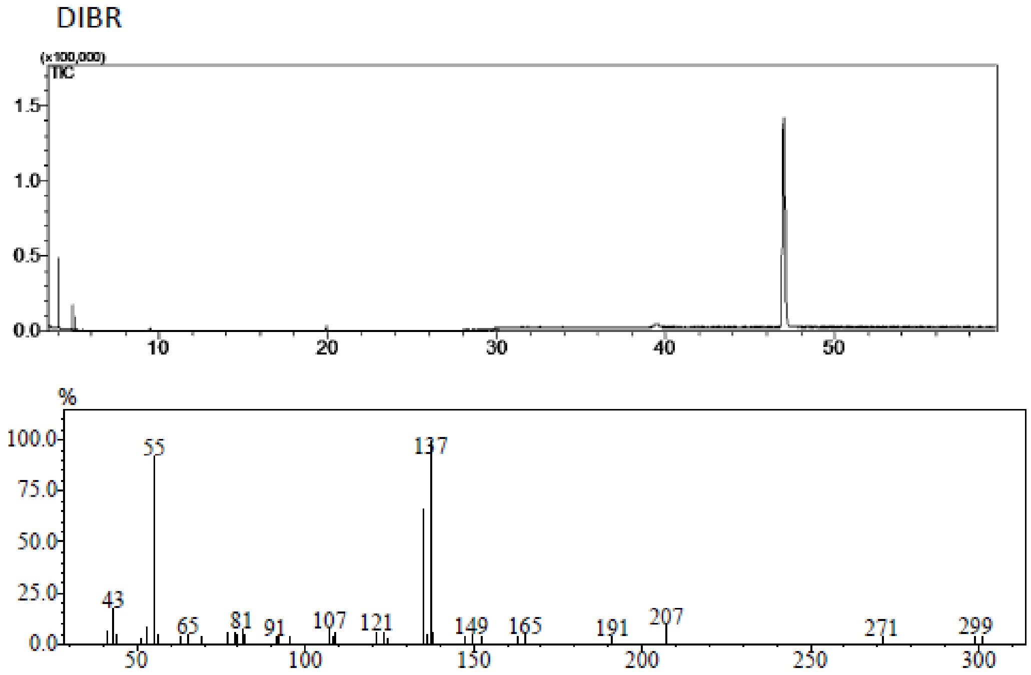

Figure S9. 1H-NMR spectrum of compound **5** ($CDCl_3$, 400 MHz).

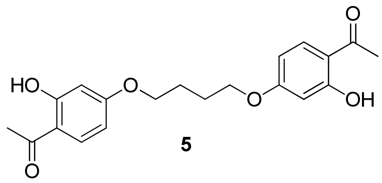

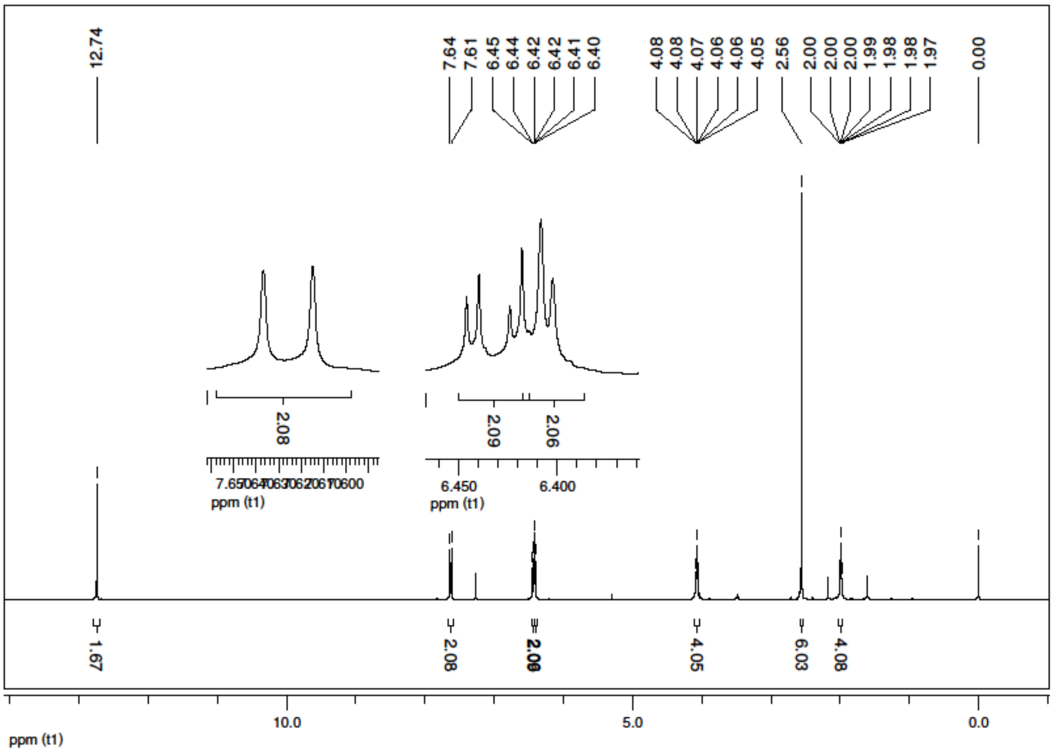

Figure S10. ^{13}C-NMR spectrum of compound **5** ($CDCl_3$, 100 MHz).

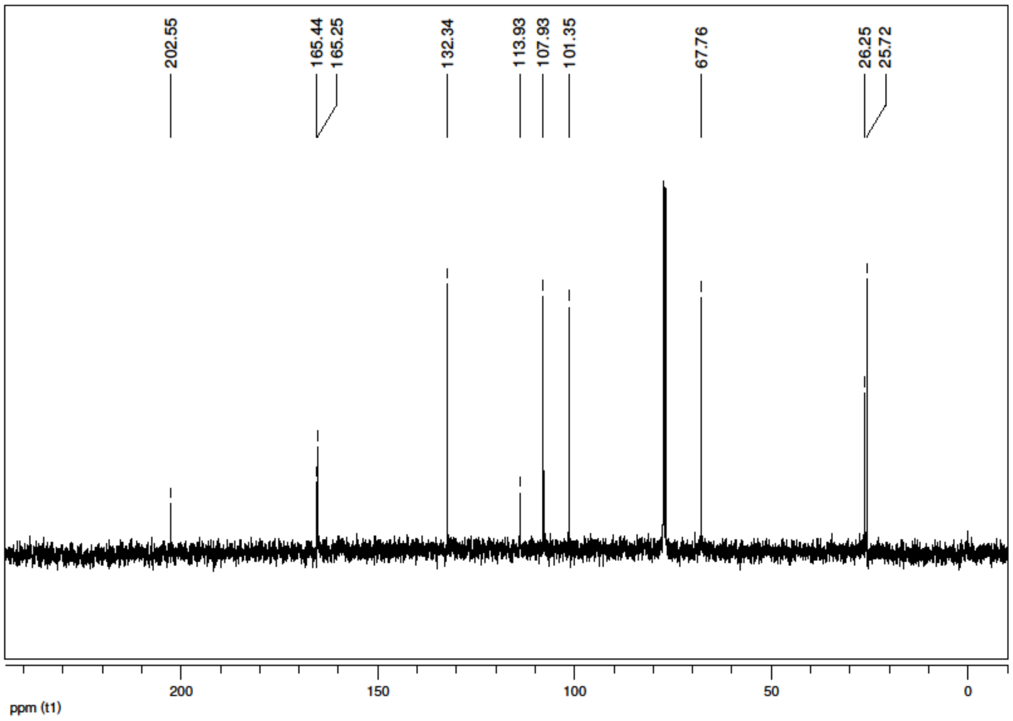

Figure S11. IR spectrum of compound **5**.

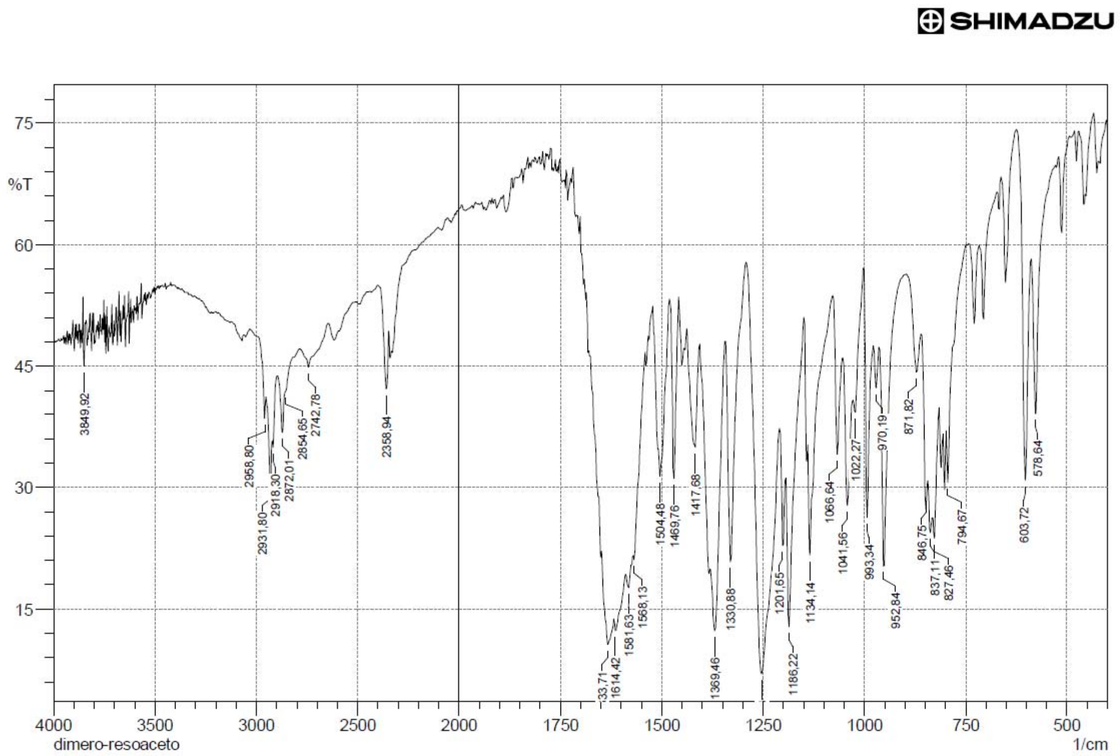

Figure S12. Mass spectrum of compound **5**.

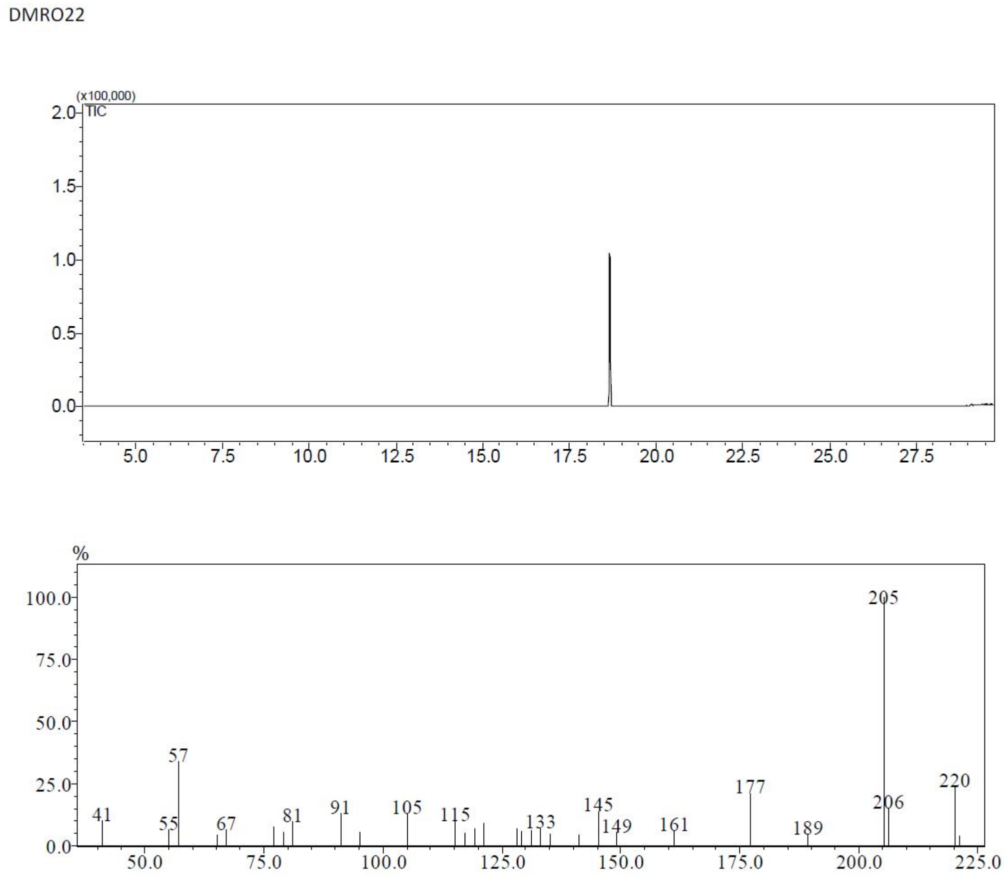

Figure S13. 1H-NMR spectrum of compound 6 ($CDCl_3$, 400 MHz).

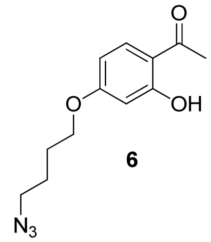

6

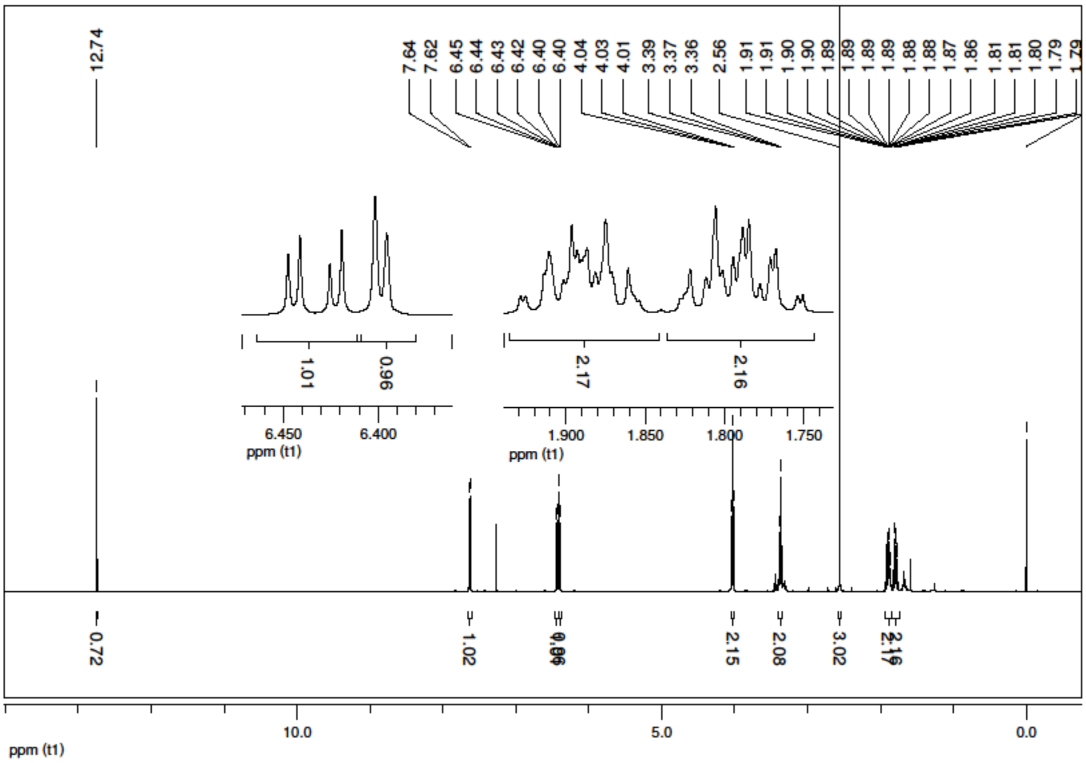

Figure S14. ¹³C-NMR spectrum of compound **6** ($CDCl_3$, 100 MHz).

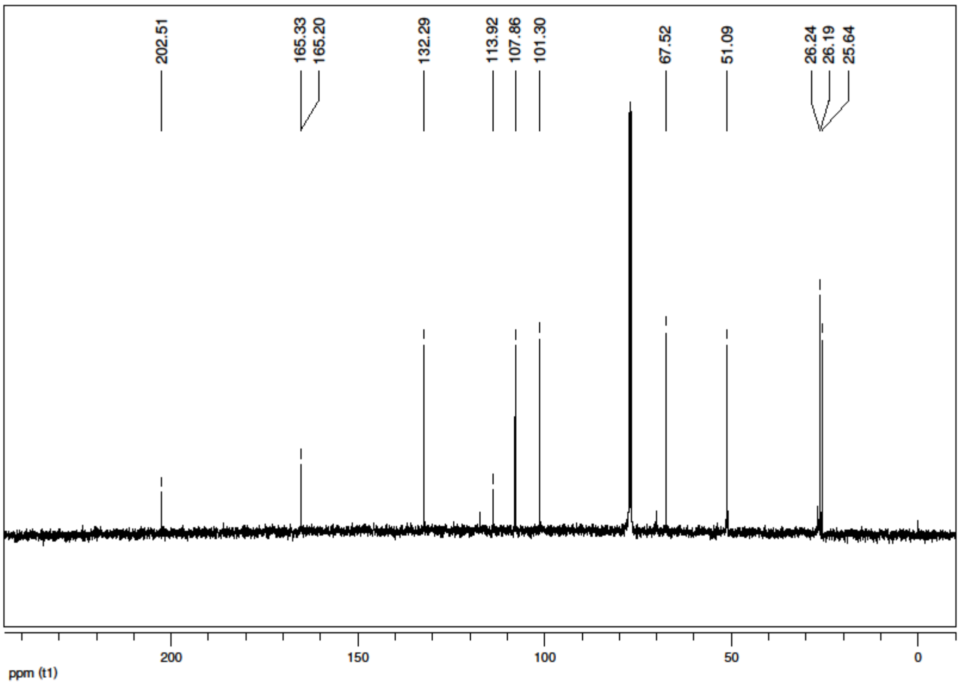

Figure S15. IR spectrum of compound **6**.

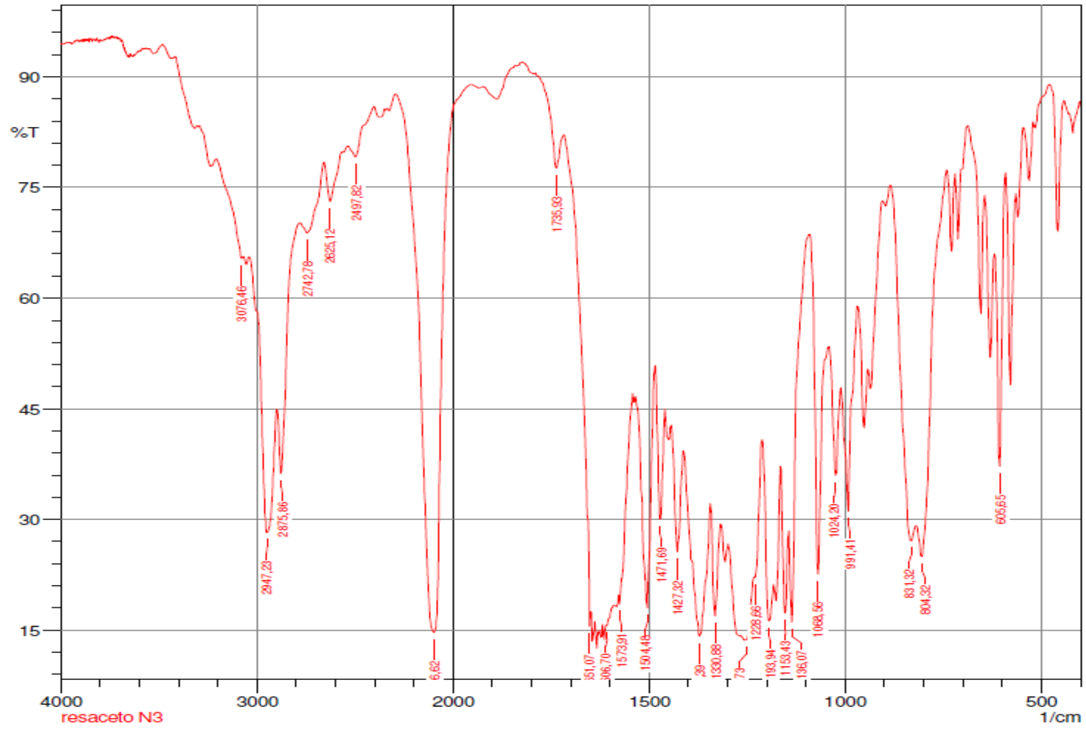

Figure S16. Mass spectra of compound **6**

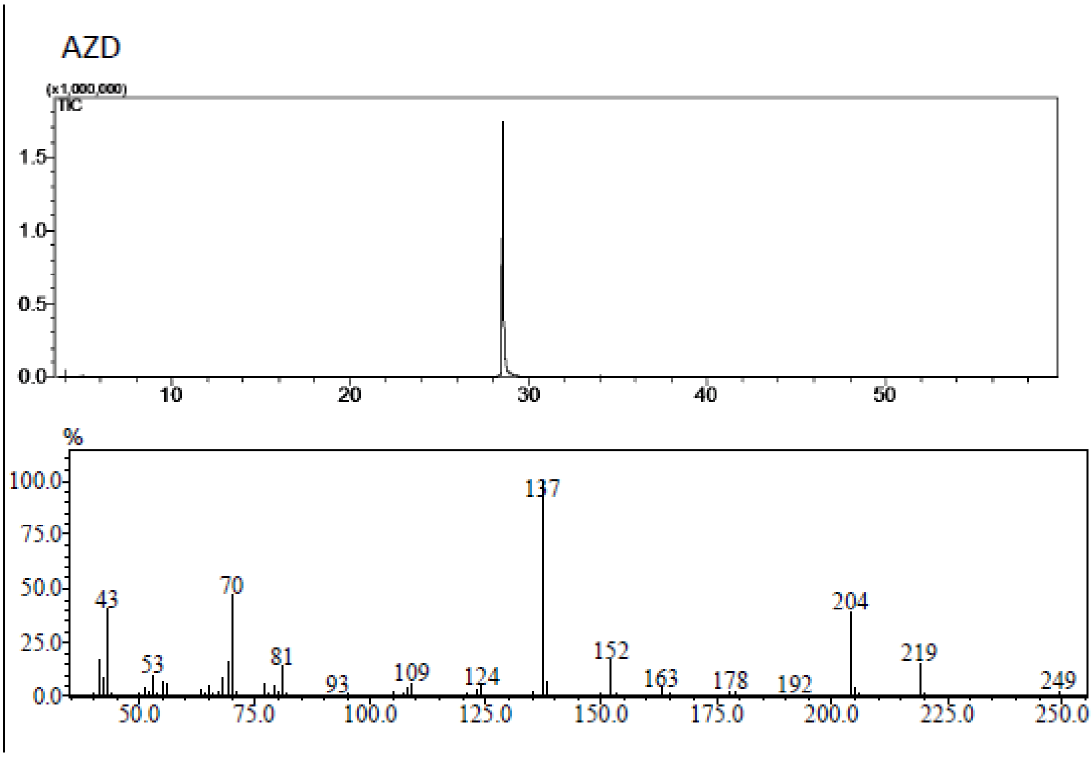

Figure S17. 1H-NMR spectrum of compound **7a** ($CDCl_3$, 400 MHz).

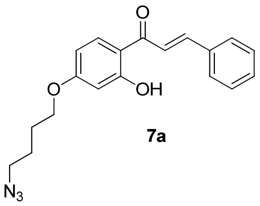

7a

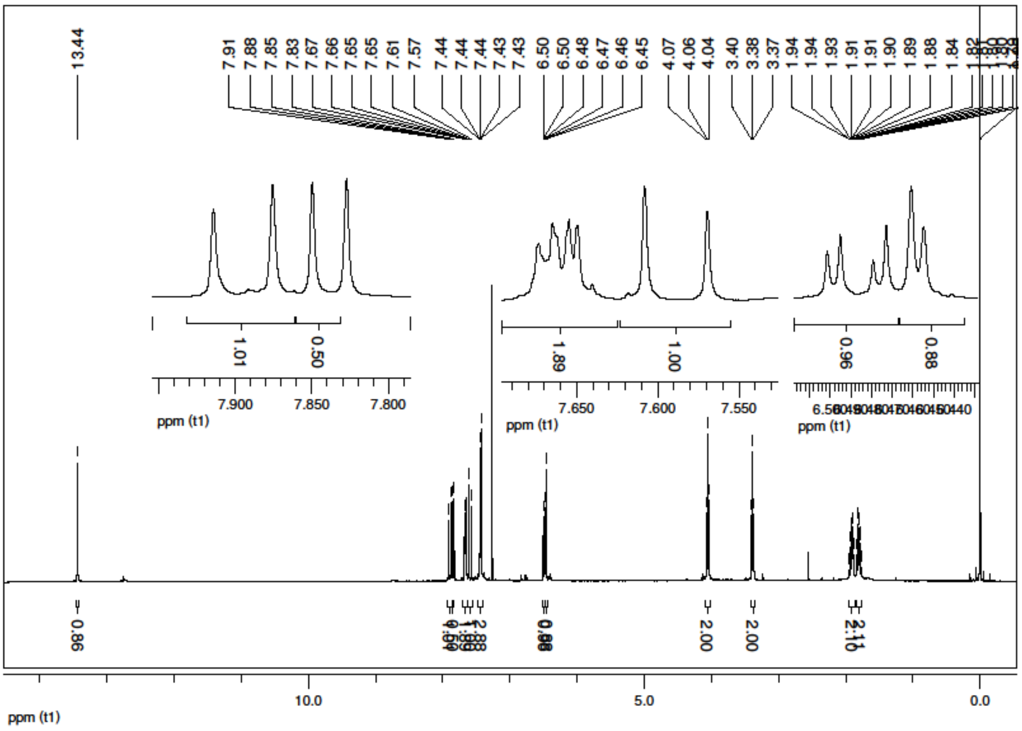

Figure S18. ^{13}C-NMR spectrum of compound **7a** ($CDCl_3$, 100 MHz).

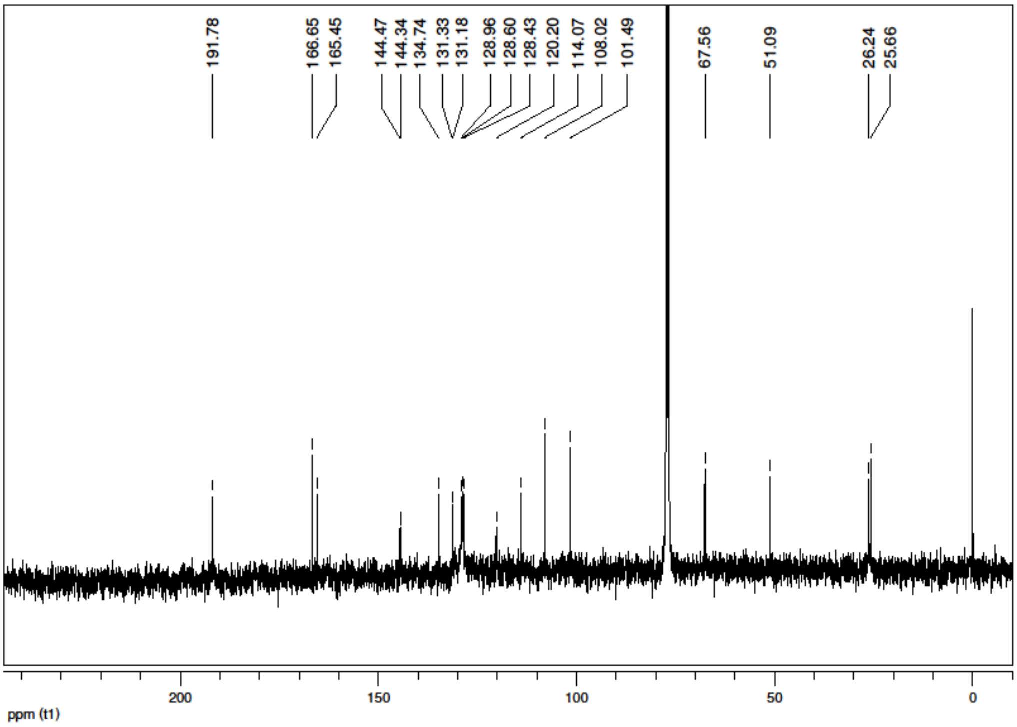

Figure S19. IR spectrum of compound **7a**.

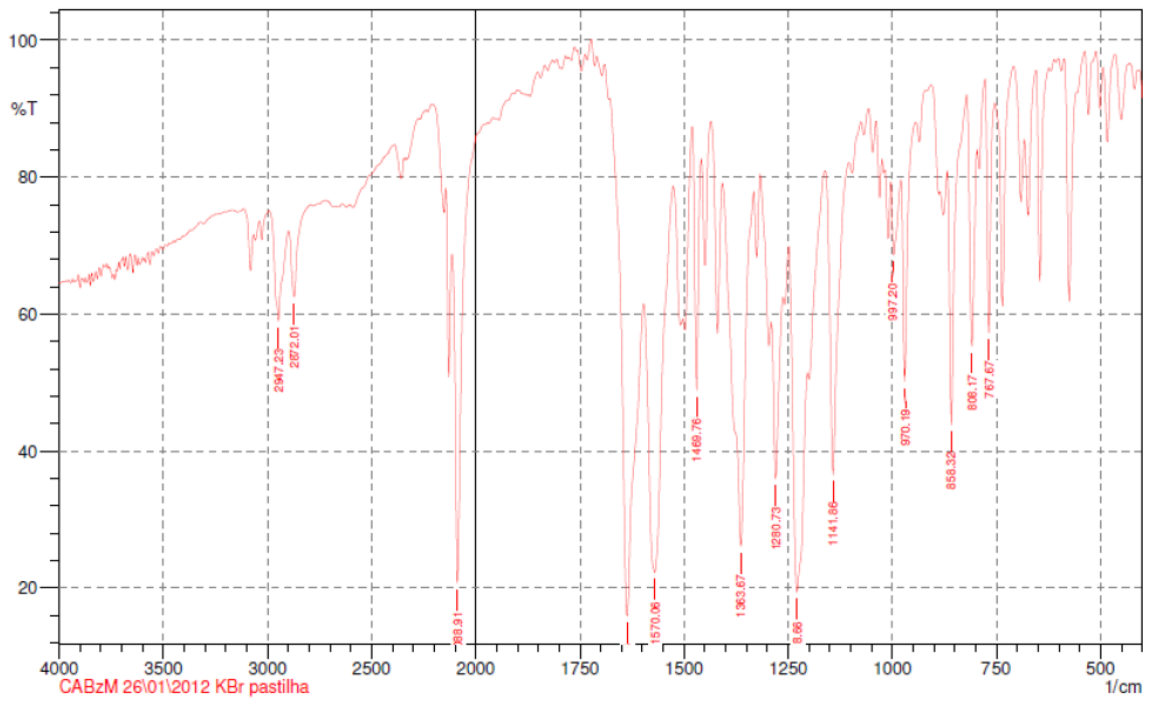

Figure S20. HRMS spectrum of compound **7a**.

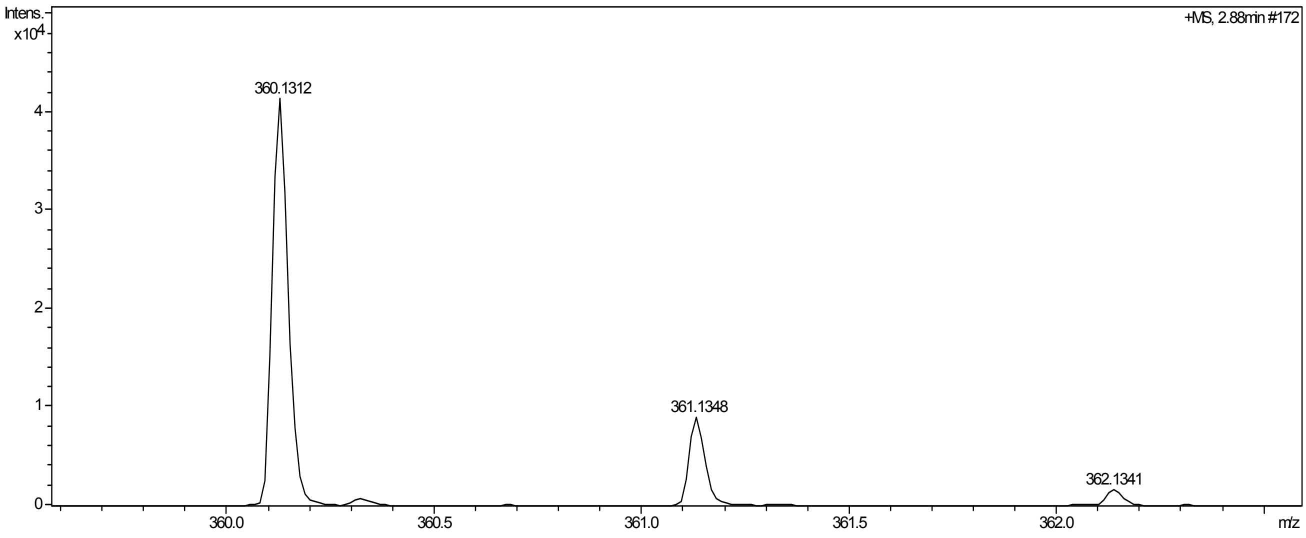

Figure S21. 1H-NMR spectrum of compound **7b** ($CDCl_3$, 400 MHz).

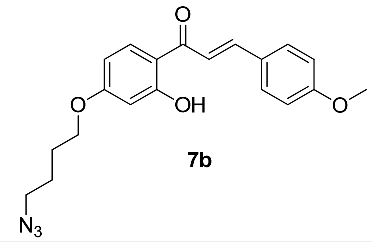

7b

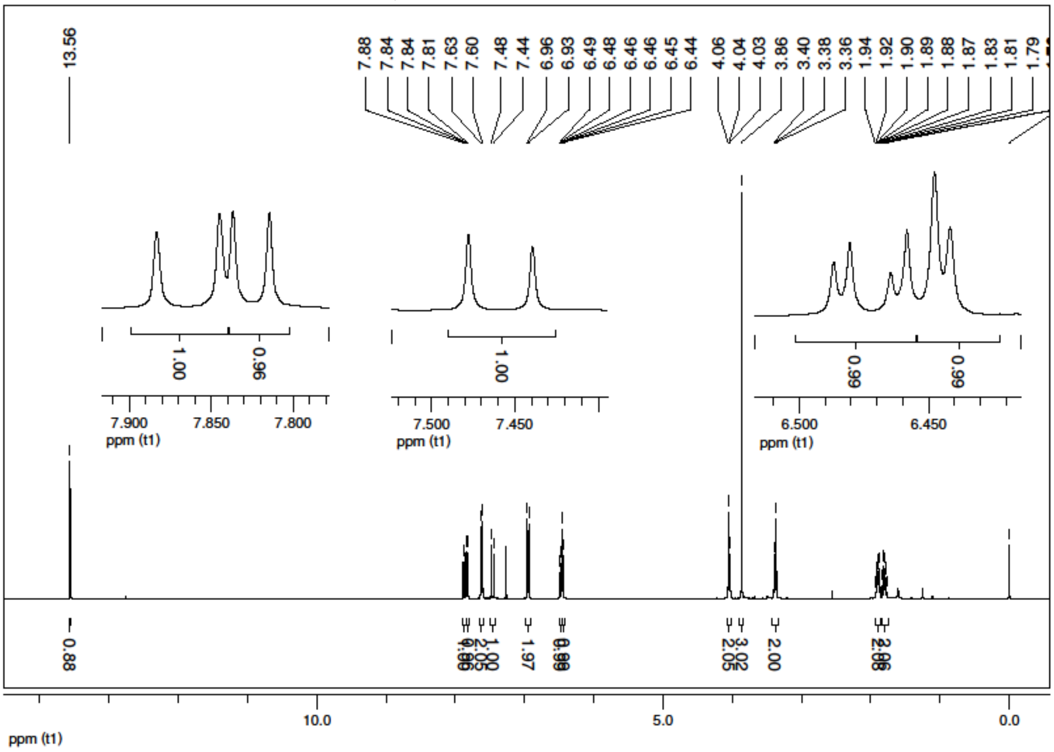

Figure S22. ^{13}C-NMR spectrum of compound **7b** ($CDCl_3$, 100 MHz).

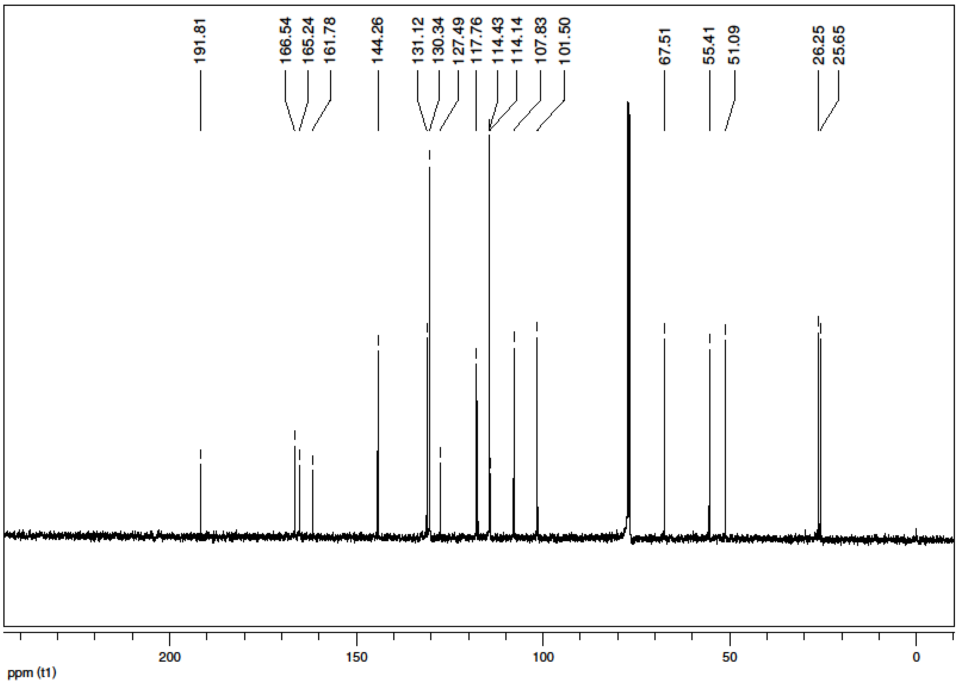

Figure S23. IR spectrum of compound **7b**.

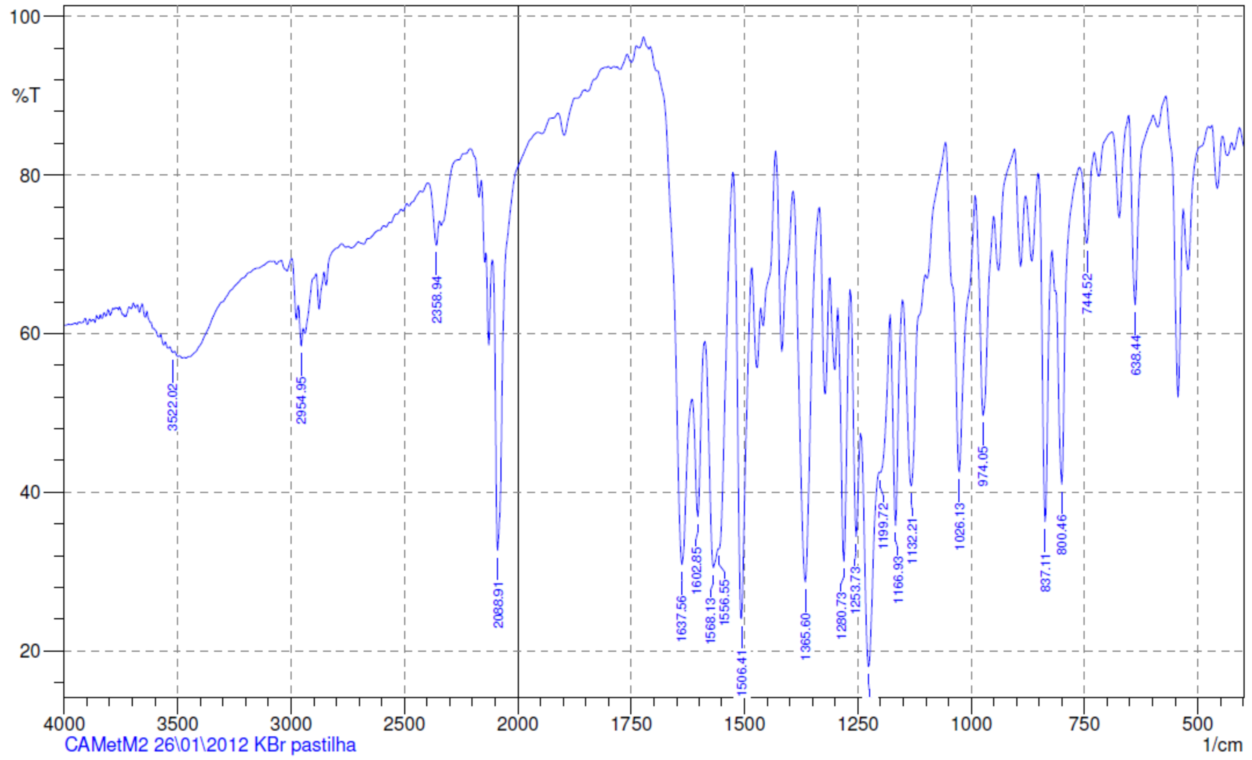

Figure S24. HRMS spectrum of **7b**.

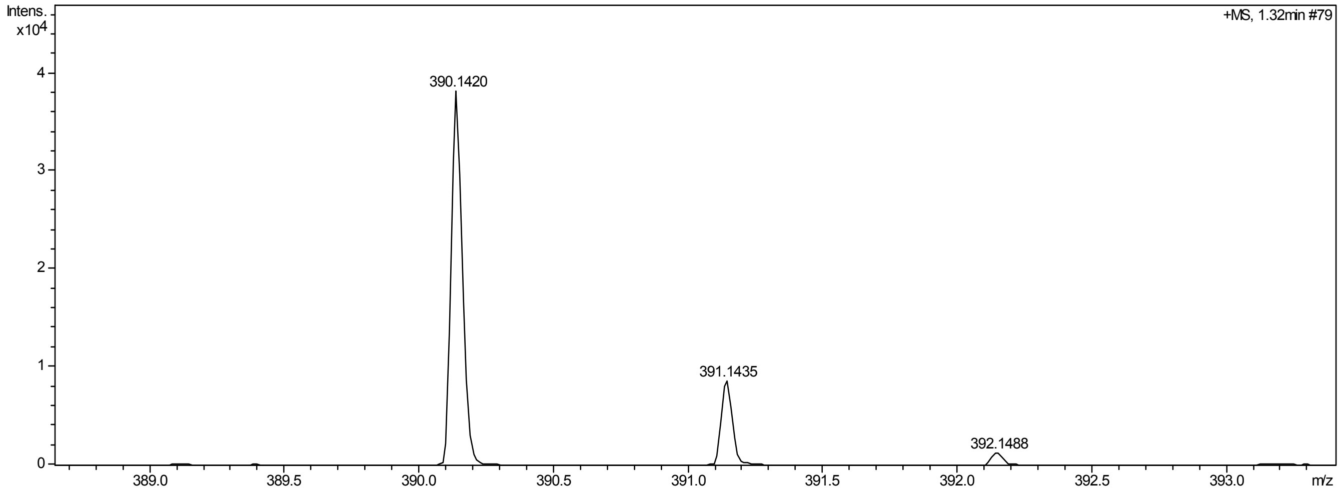

Figure S25. 1H-NMR spectrum **7c** ($CDCl_3$, 400 MHz).

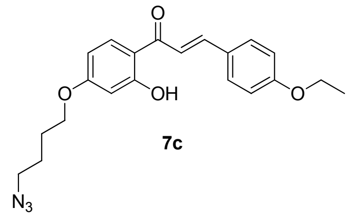

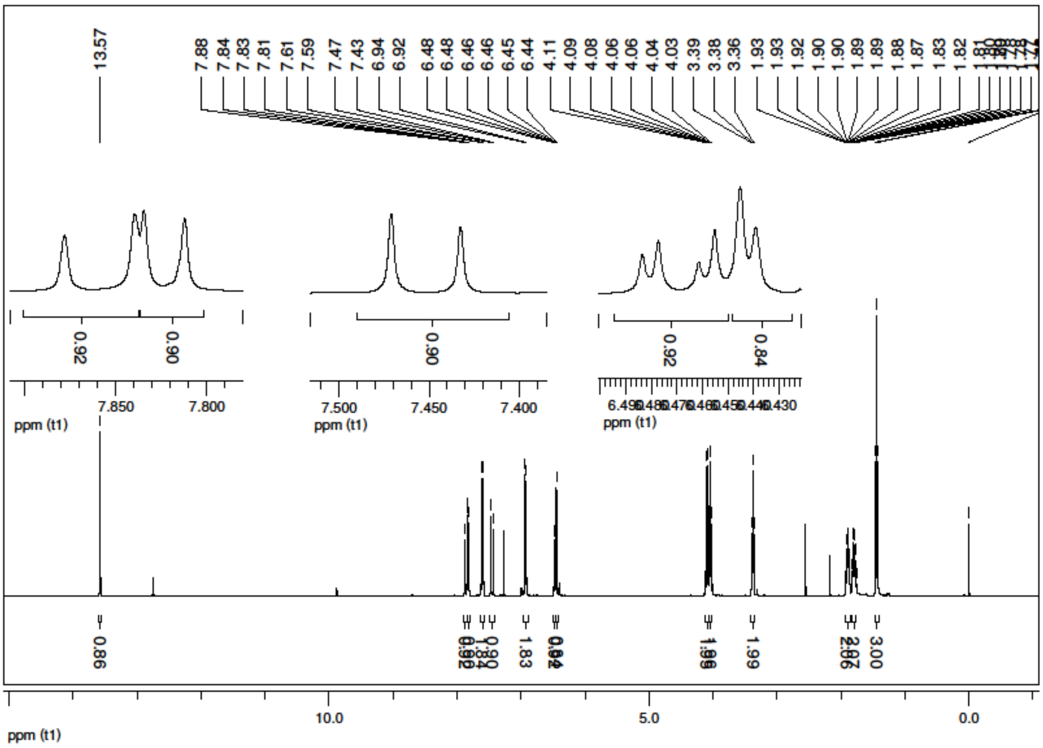

Figure S26. ^{13}C-NMR spectrum of compound **7c** ($CDCl_3$, 100 MHz).

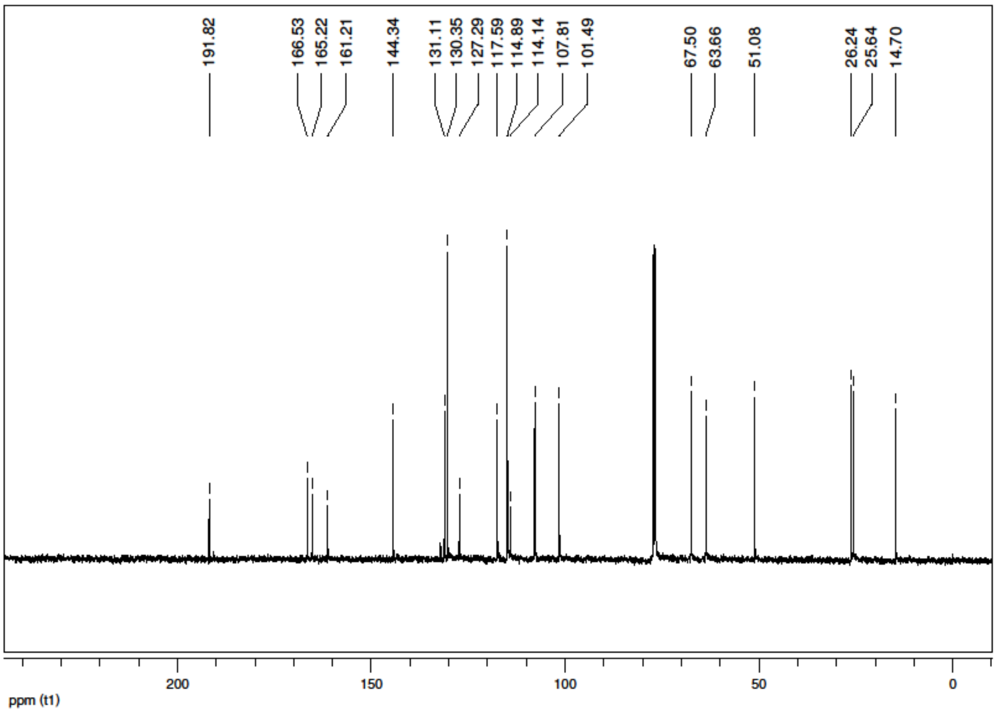

Figure S27. IR spectrum of compound **7c**.

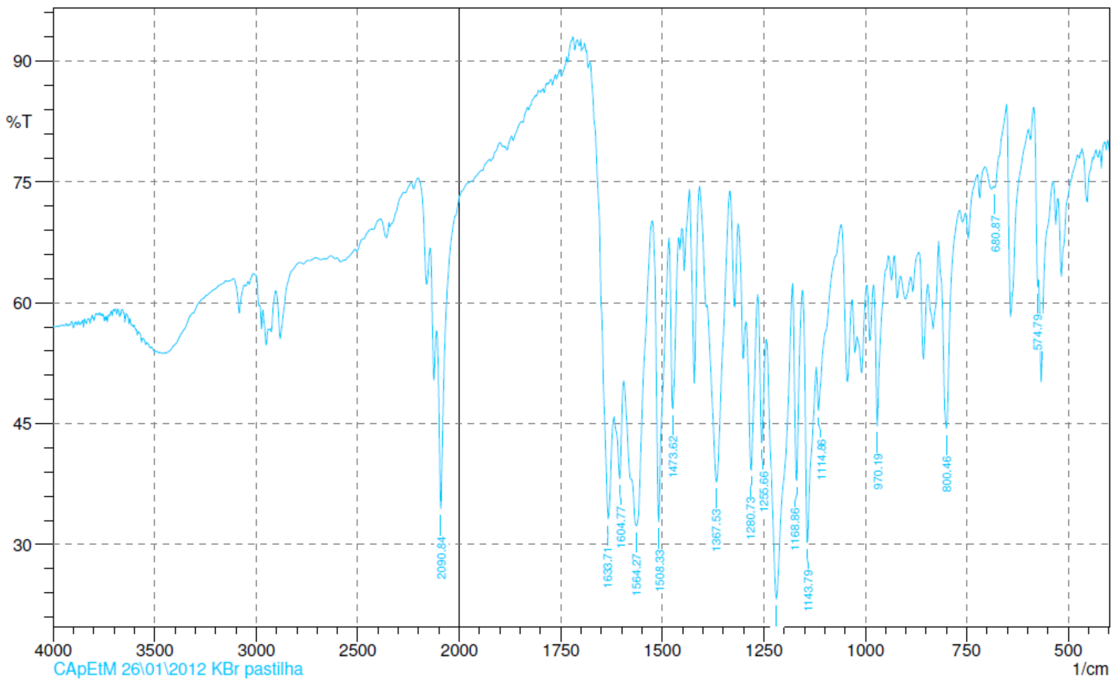

Figure S28. HRMS spectrum of compound **7c**.

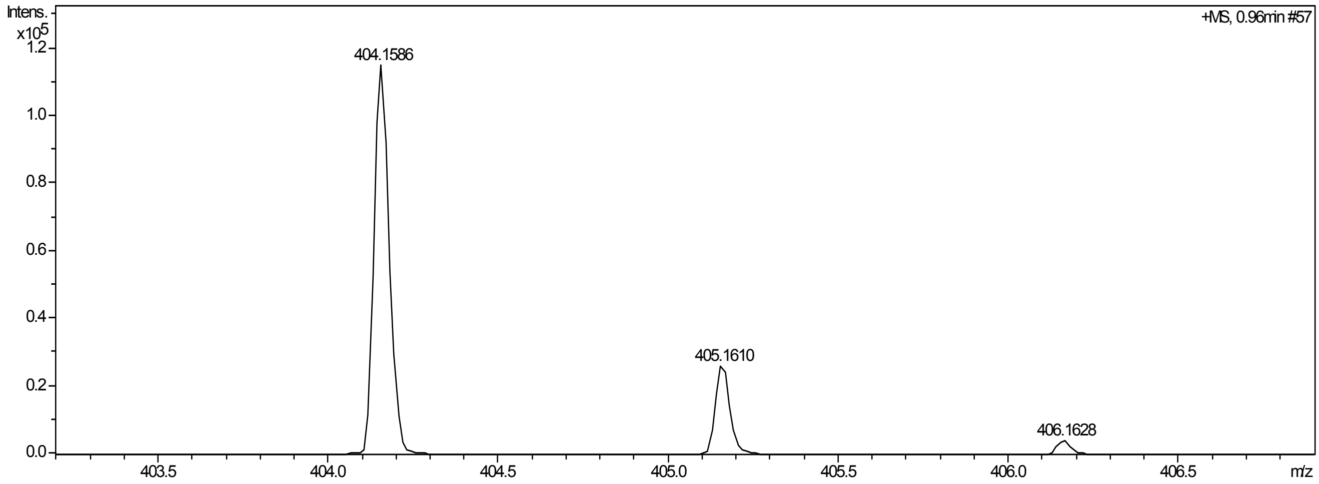

Figure S29. 1H-NMR spectrum of compound **7d** (DMSO-*d*6, 400 MHz).

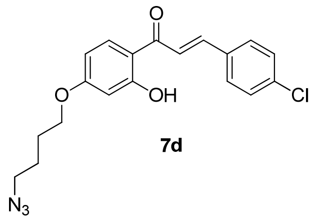

7d

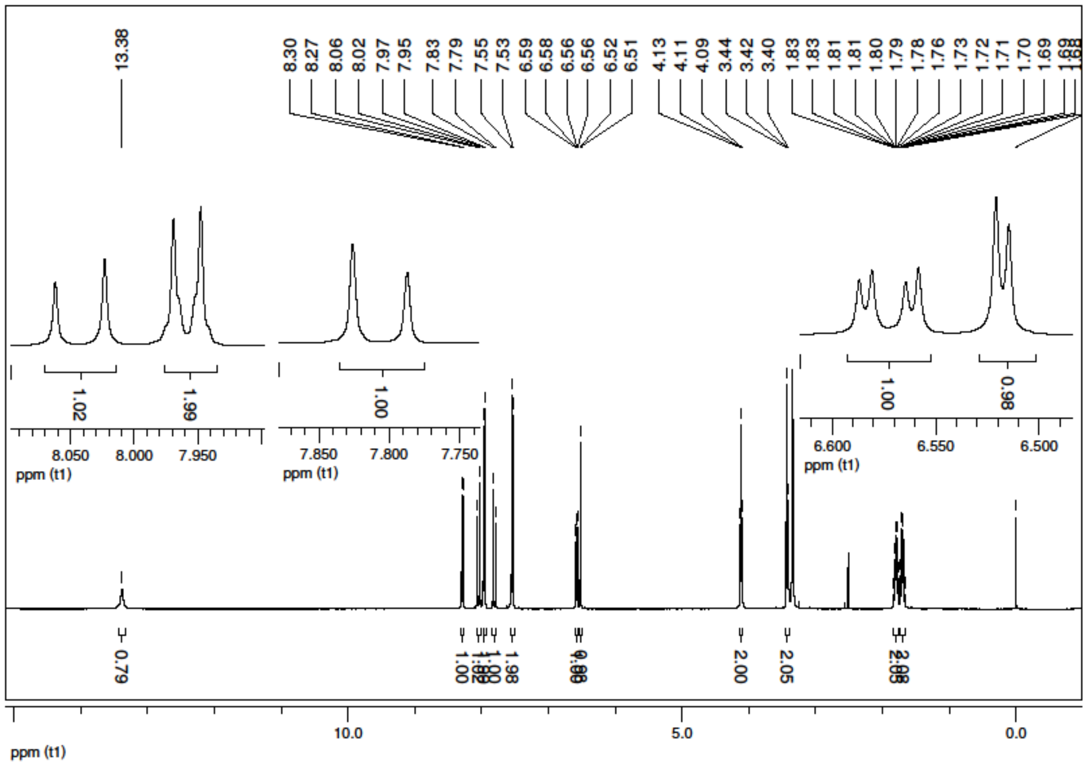

Figure S30. ^{13}C-NMR spectrum of compound **7d** (DMSO-d_6, 100 MHz).

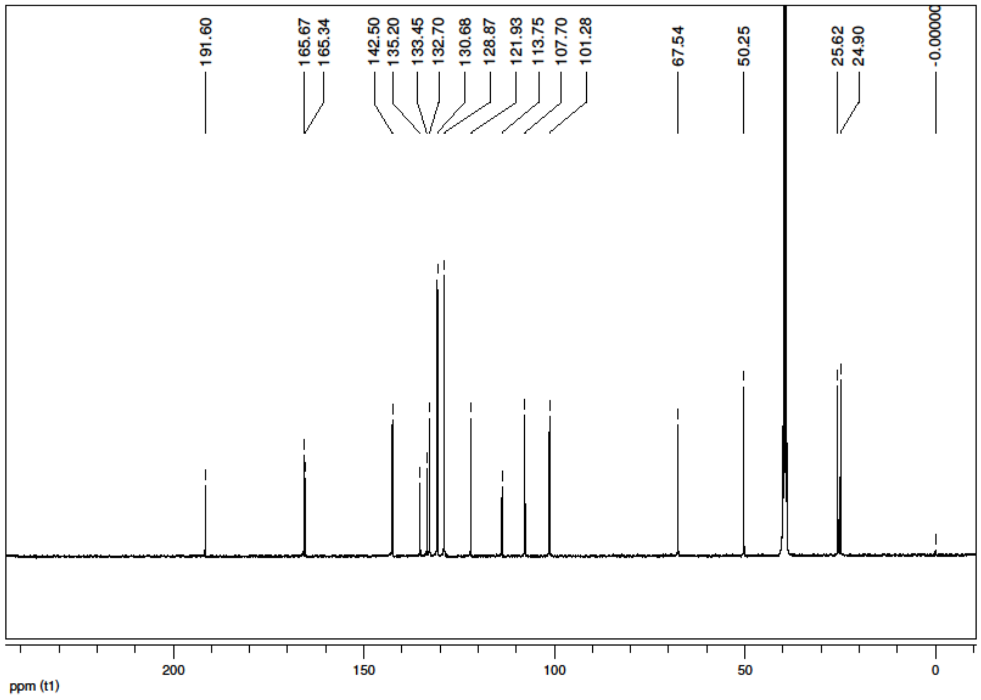

Figure S31. IR spectrum of compound **7d**.

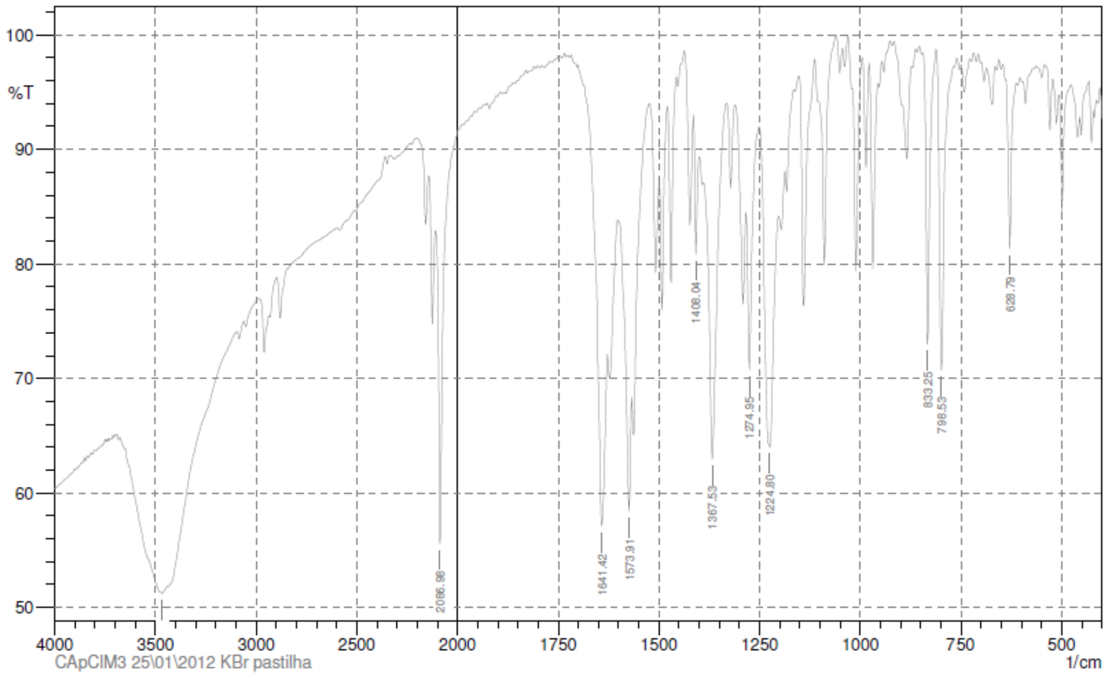

Figure S32. HRMS spectrum of compound **7d**.

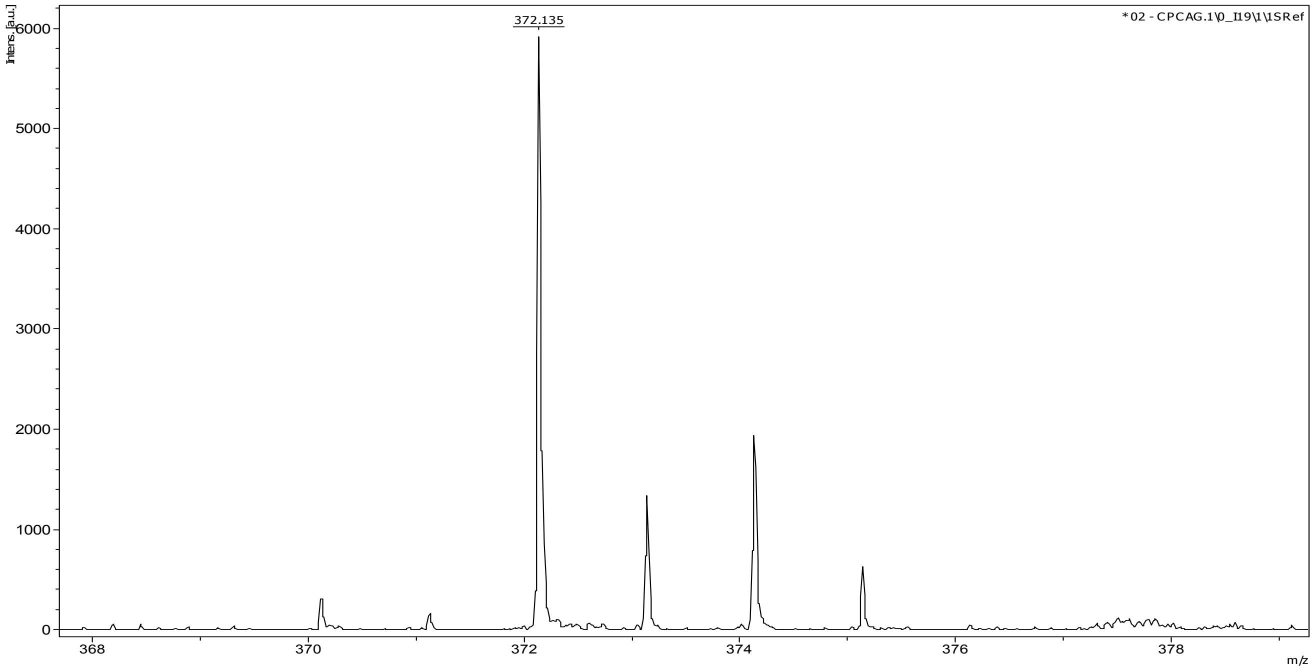

Figure S33. 1H-NMR spectrum of compound **7e** ($CDCl_3$, 400 MHz).

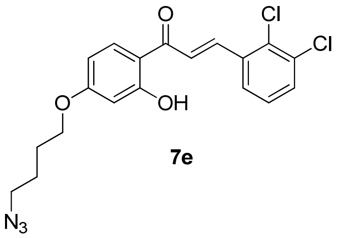

7e

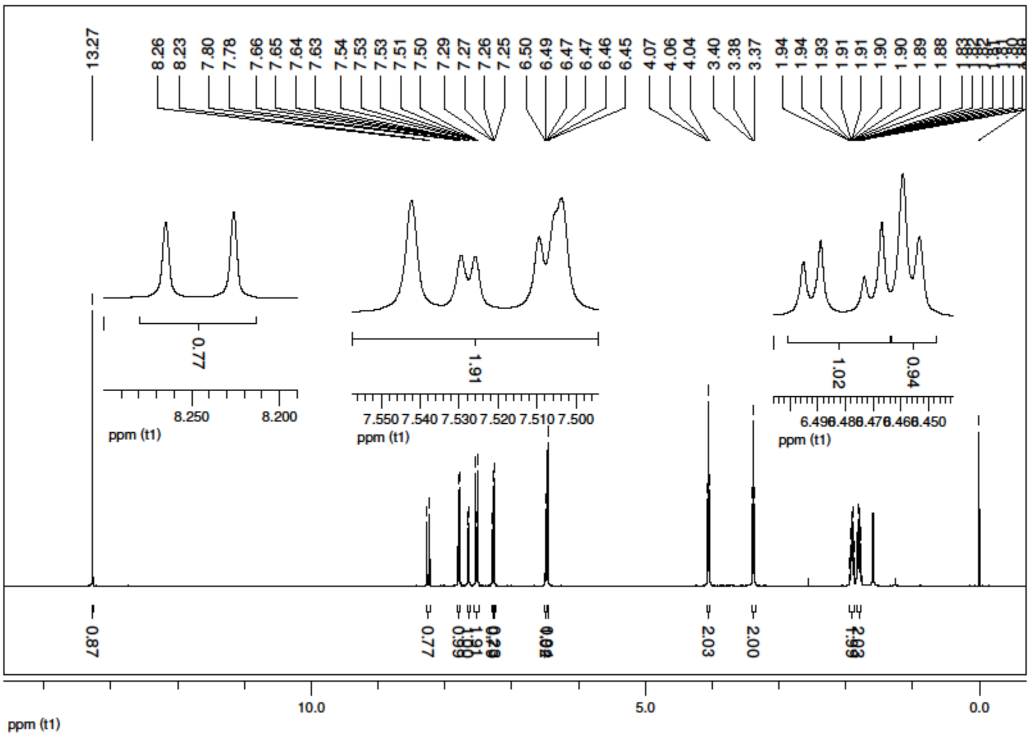

Figure S34. ^{13}C-NMR spectrum of compound **7e** ($CDCl_3$, 100 MHz).

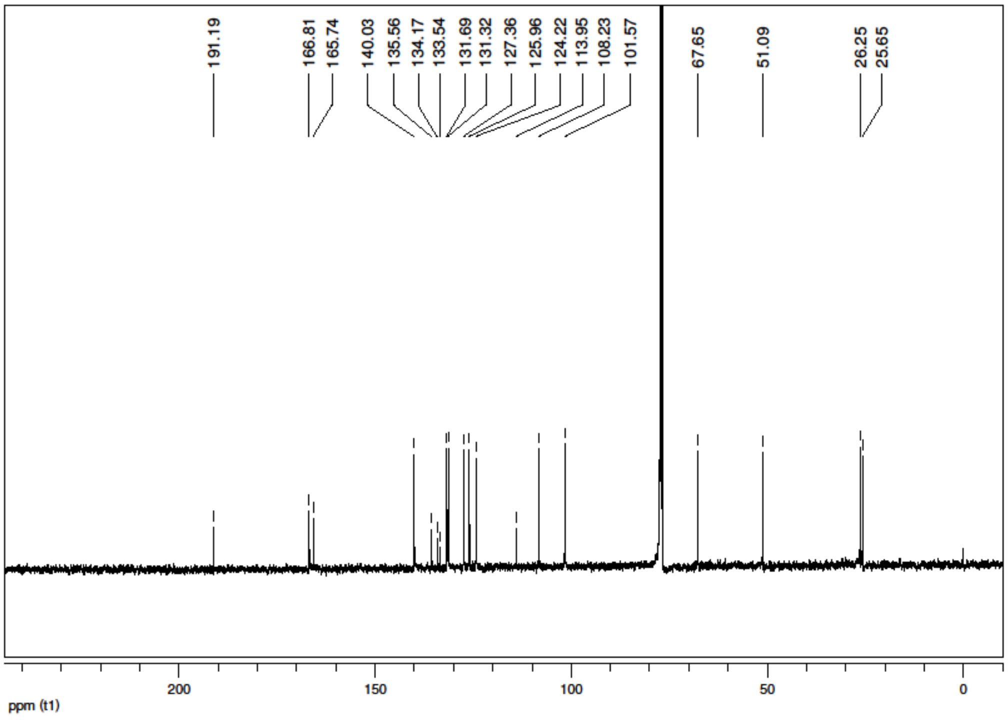

Figure S35. IR spectrum of compound **7e**.

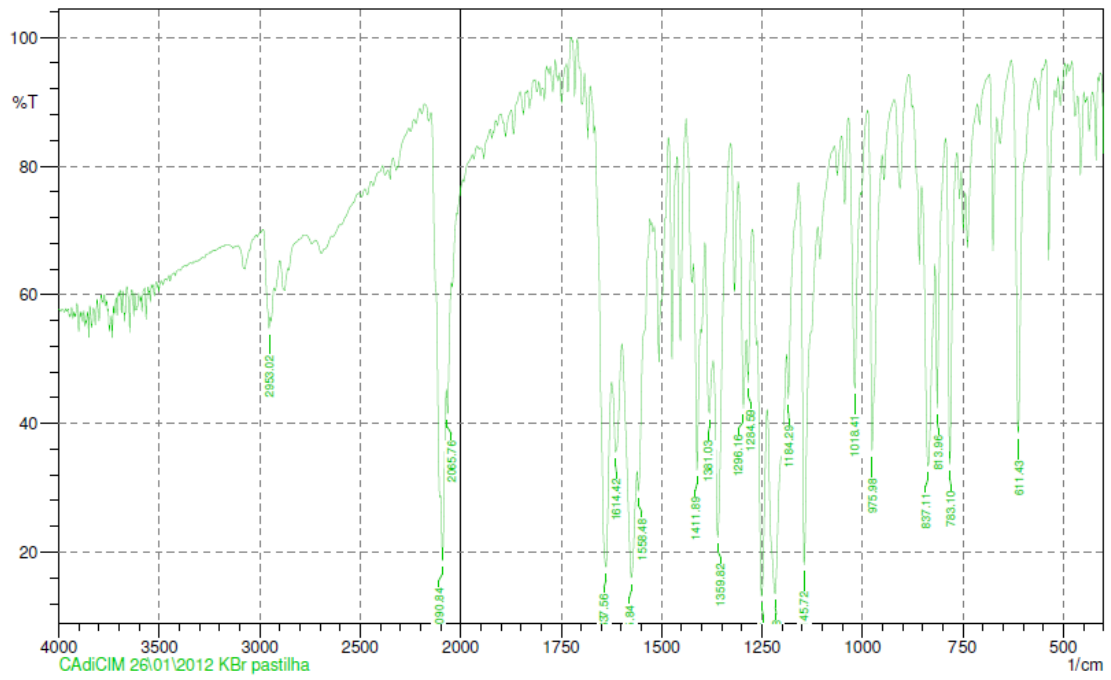

Figure S36. HRMS spectrum of compound **7e**.

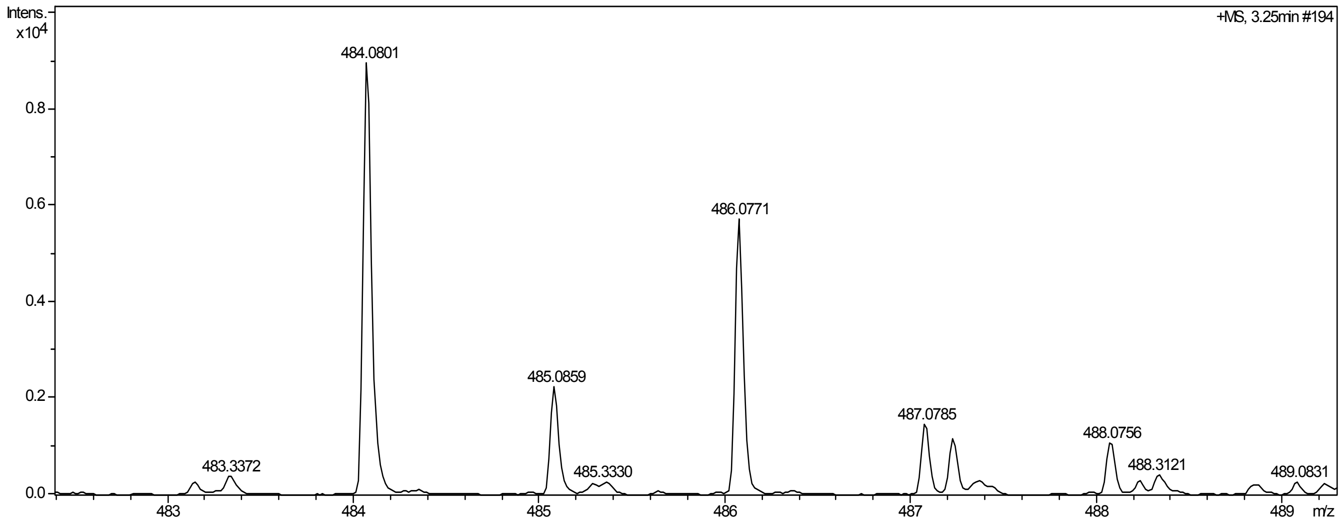

Figure S37. 1H-NMR spectrum of compound **8a** (DMSO-d_6, 400 MHz).

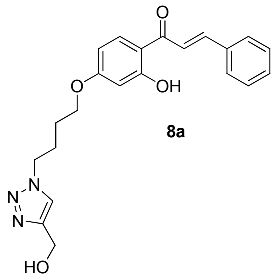

8a

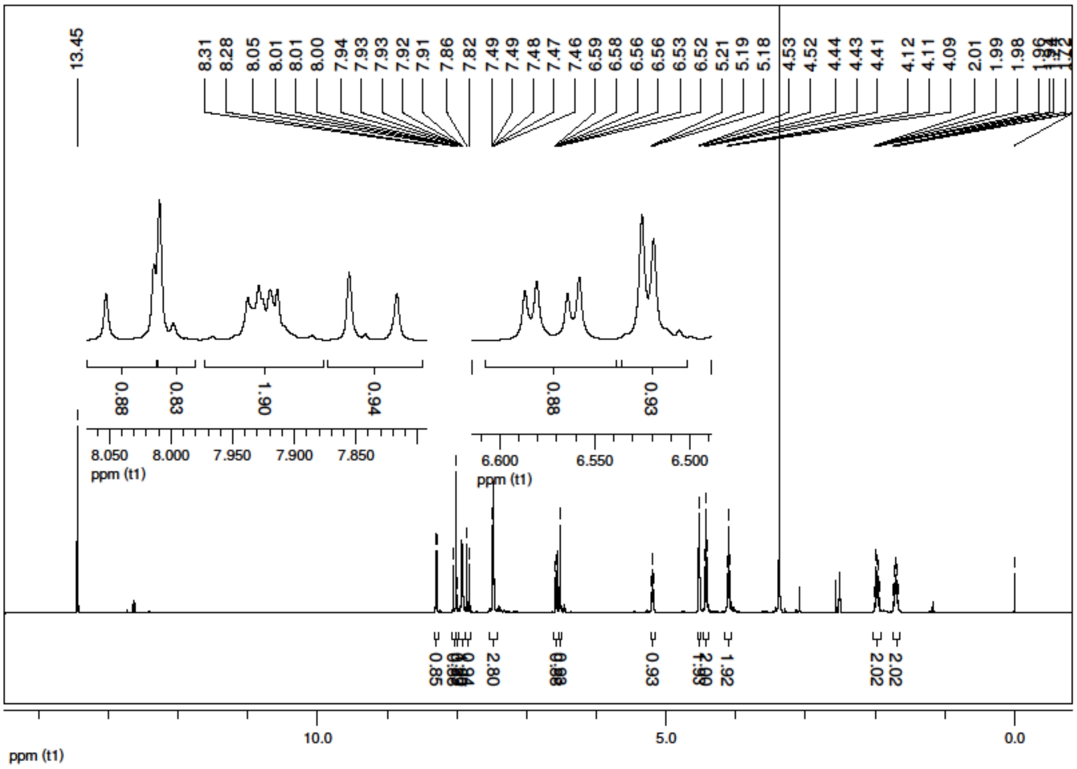

Figure S38. ^{13}C-NMR spectrum of compound **8a** (DMSO-d_6, 100 MHz).

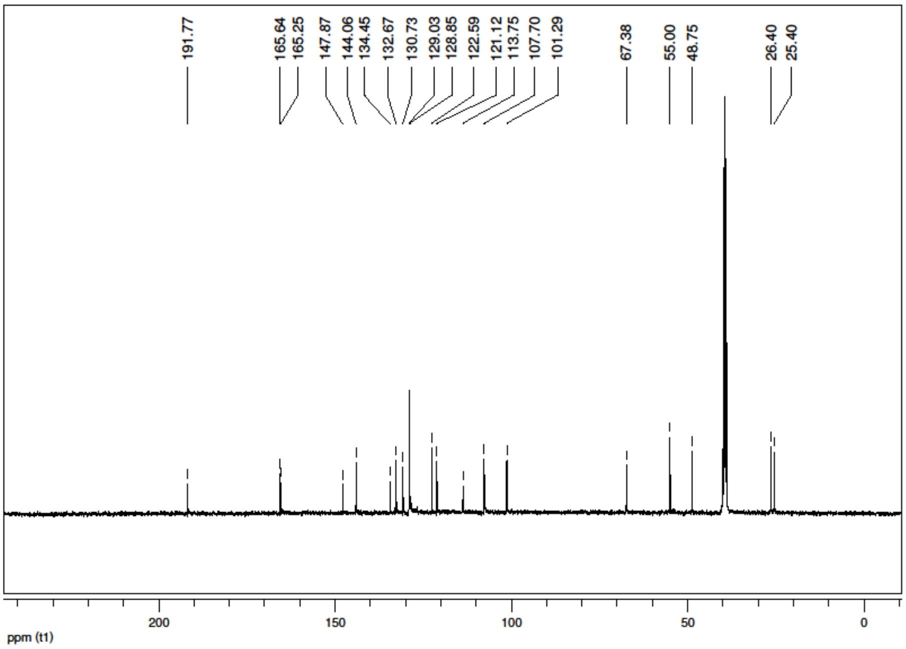

Figure S39. IR spectrum of compound **8a**.

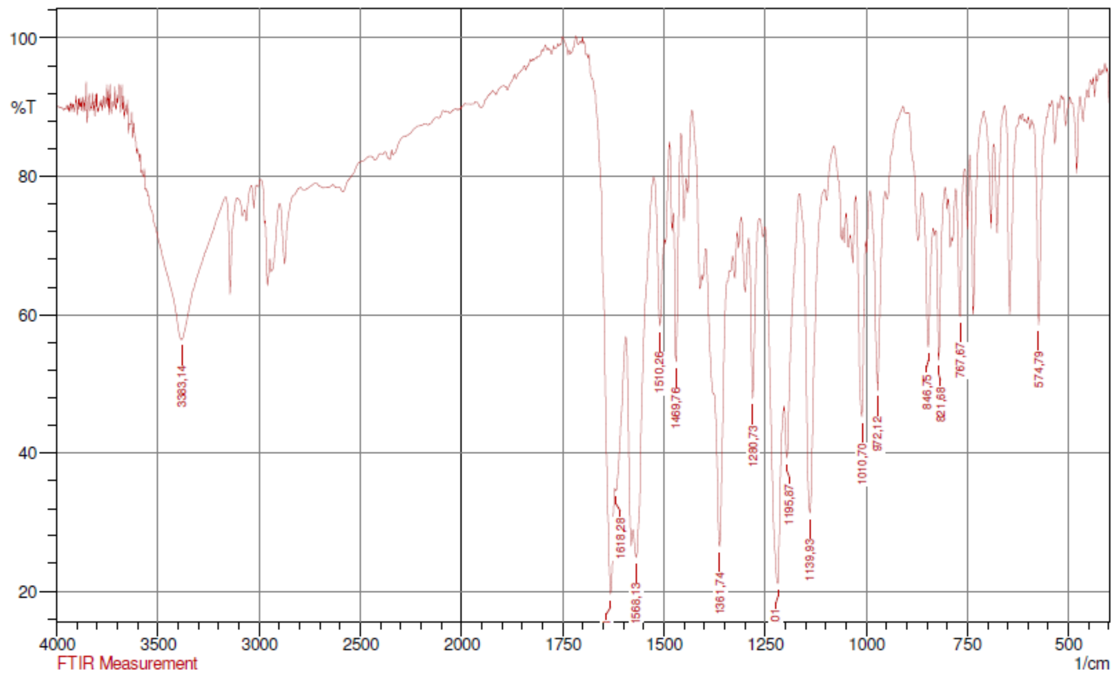

Figure S40. HRMS spectrum of compound **8a**.

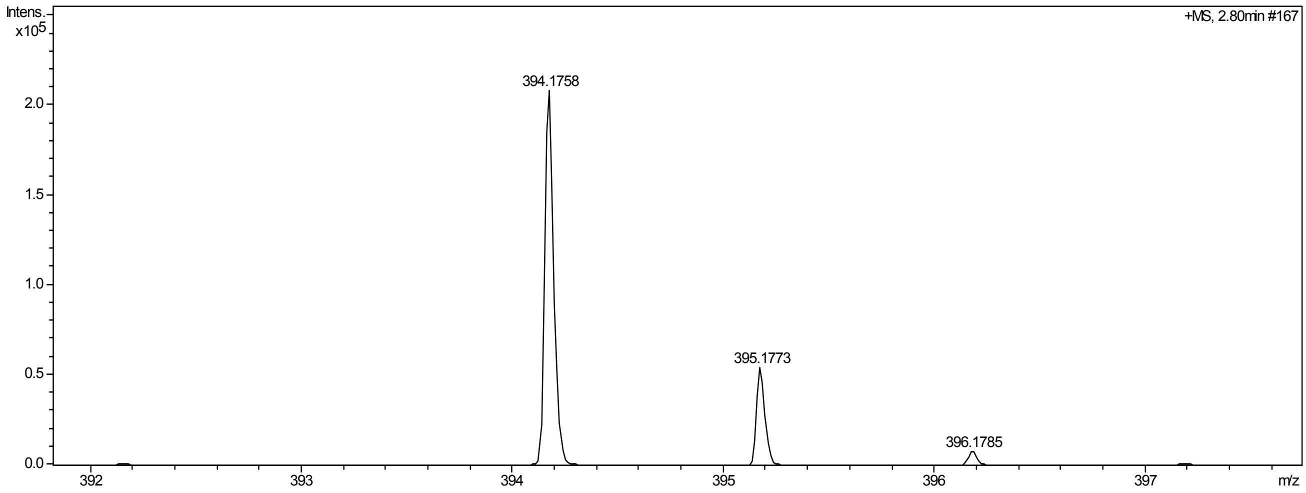

Figure S41. 1H-NMR spectrum of compound **8b** ($CDCl_3$/DMSO-d_6, 400 MHz).

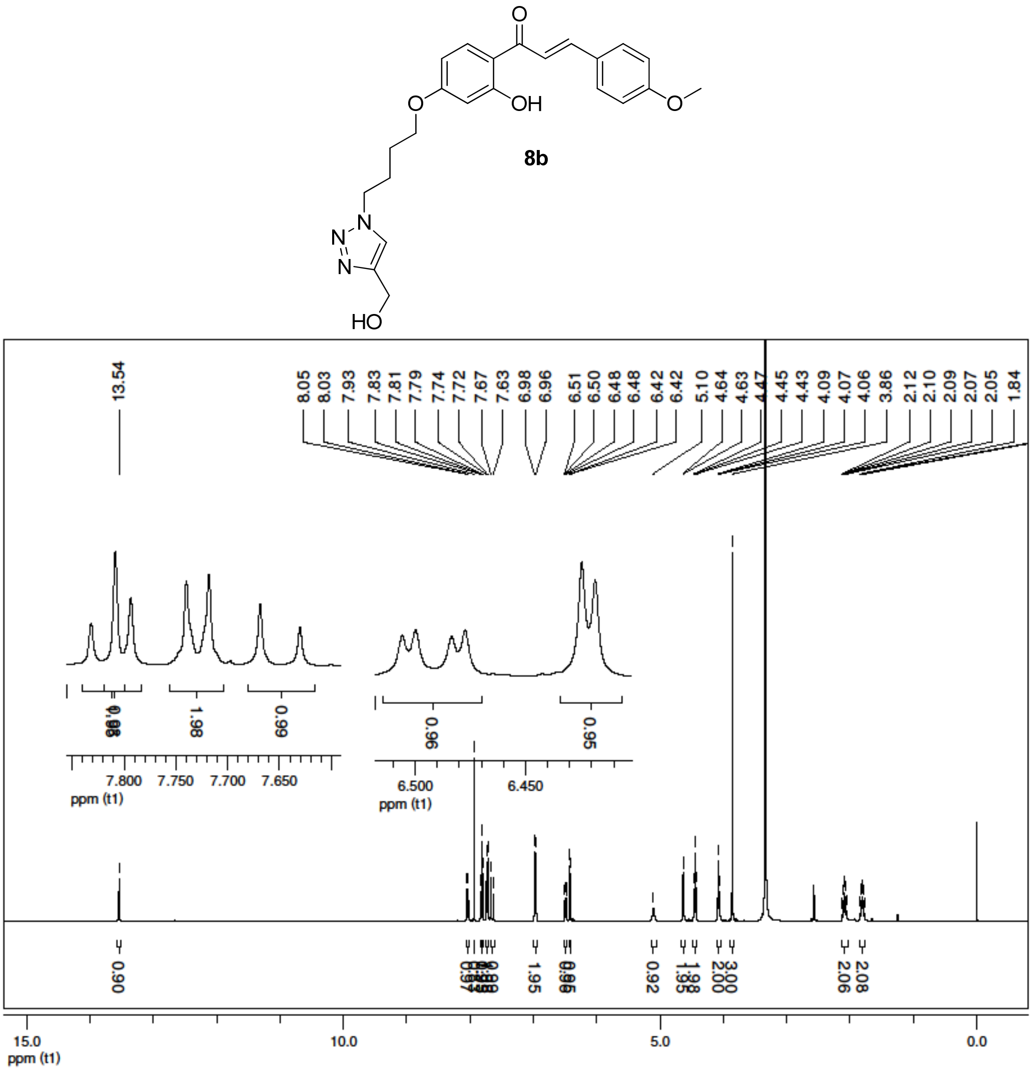

Figure S42. ^{13}C-NMR spectrum of compound **8b** ($CDCl_3$/DMSO-d_6, 100 MHz).

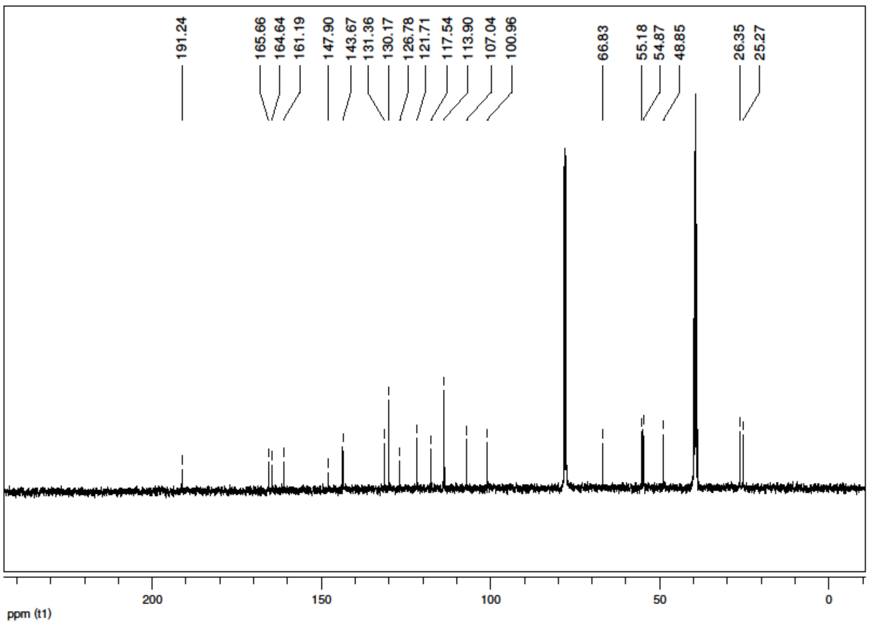

Figure S43. IR spectrum of compound **8b**.

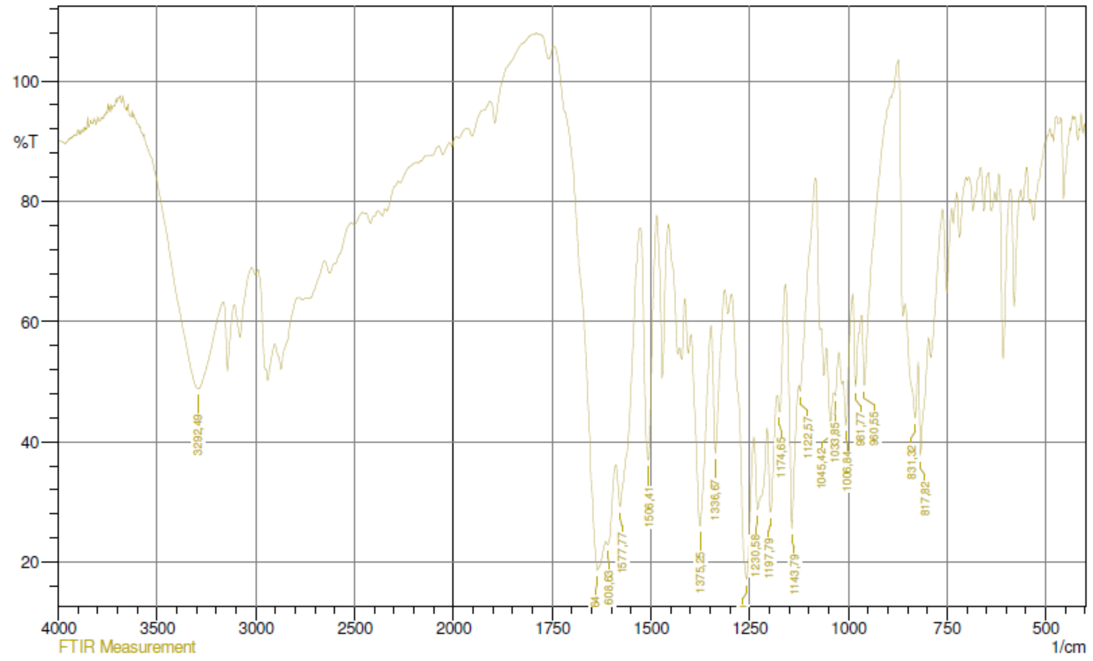

Figure S44. HRMS spectrum of compound **8b**.

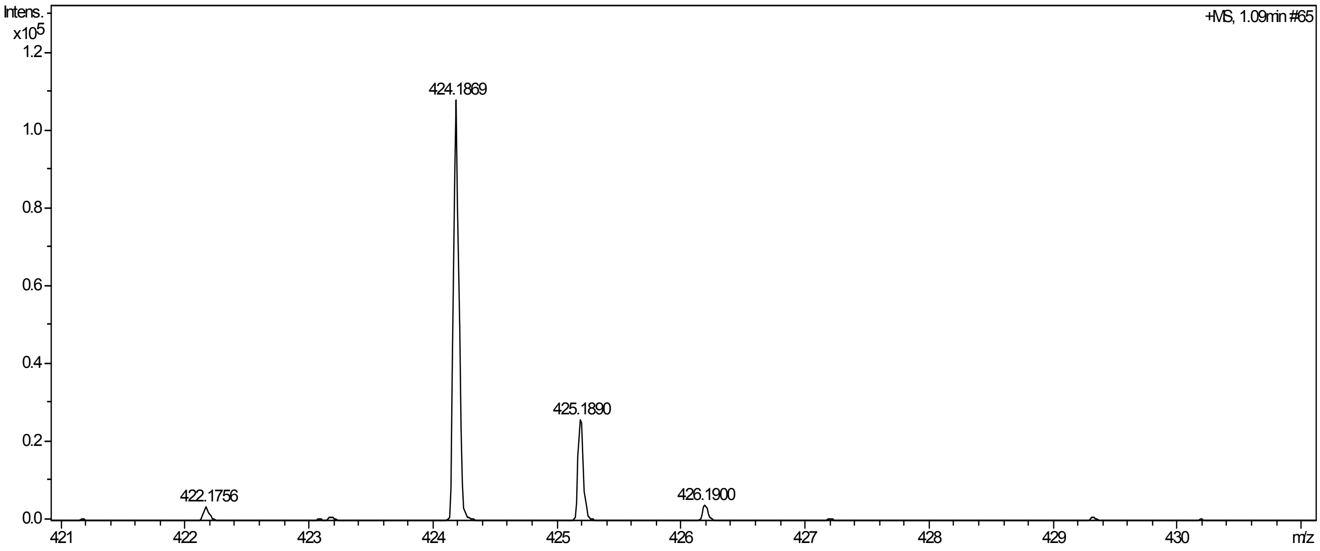

Figure S45. 1H-NMR spectrum of compound **8c** ($CDCl_3$, 400 MHz).

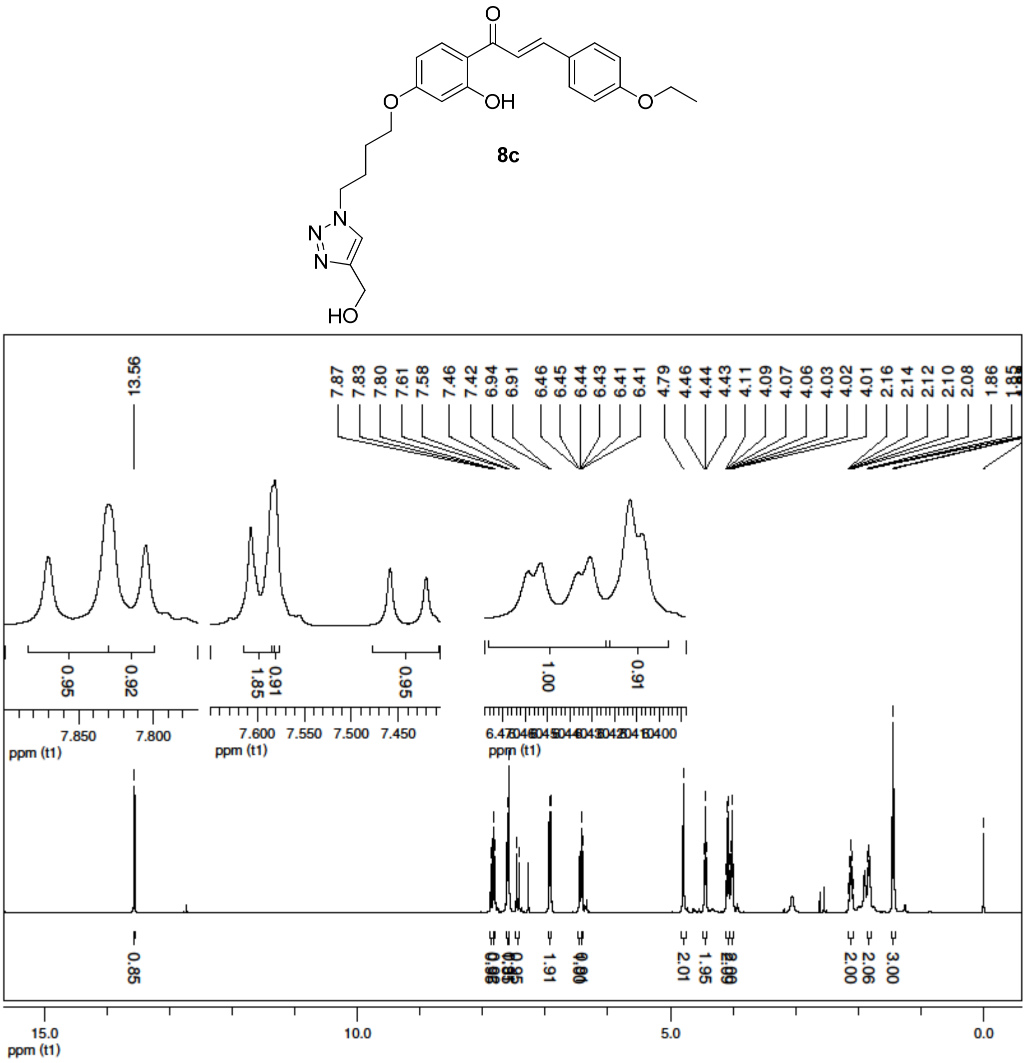

Figure S46. ^{13}C-NMR spectrum of compound **8c** ($CDCl_3$, 100 MHz).

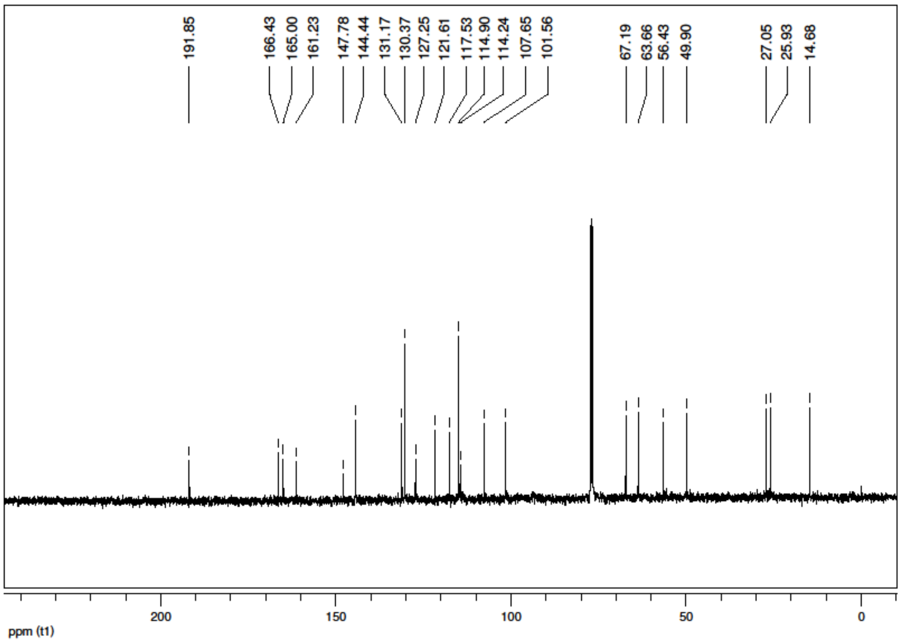

Figure S47. IR spectrum of compound **8c**.

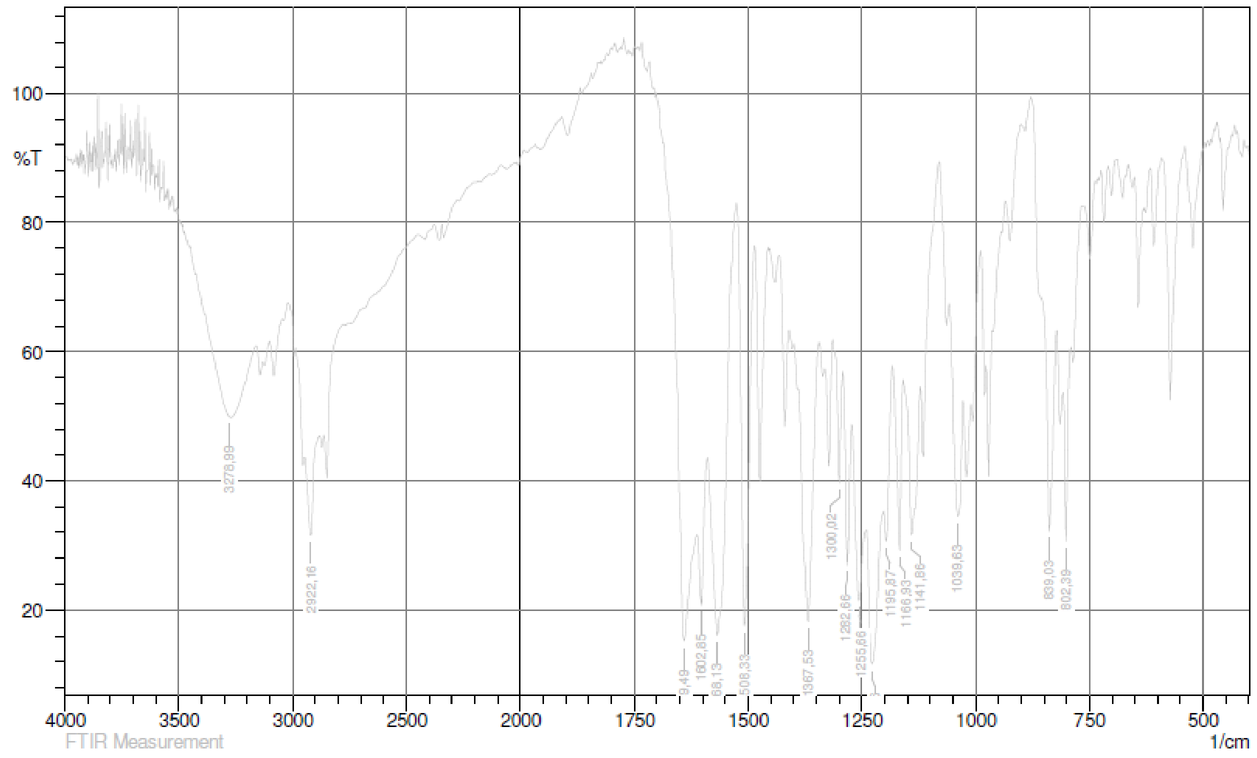

Figure S48. HRMS spectrum of compound **8c**.

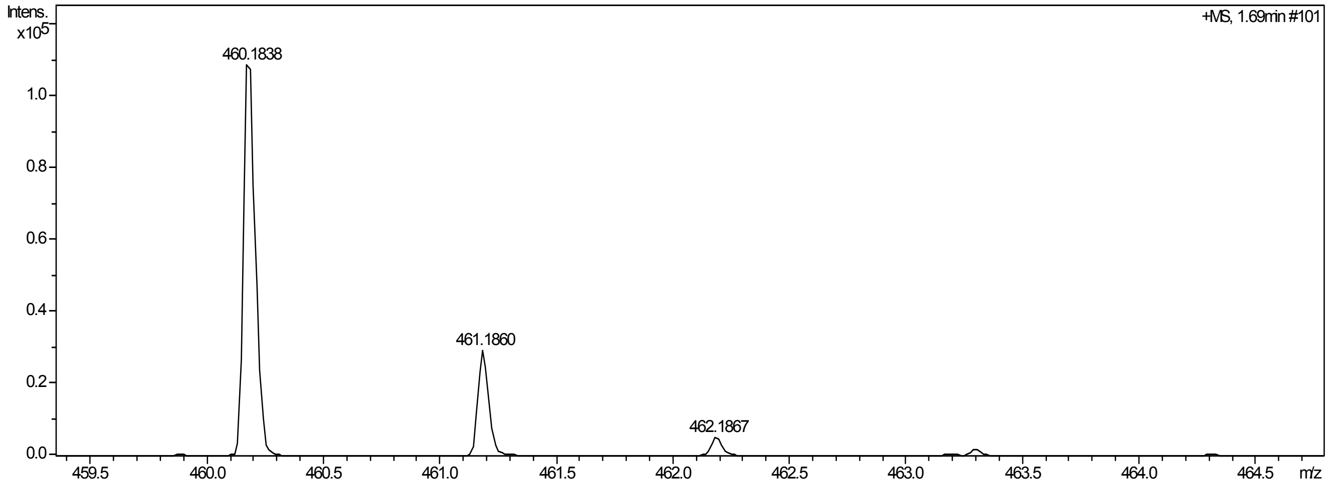

Figure S49. 1H-NMR spectrum of compound **8d** (DMSO-d_6, 400 MHz).

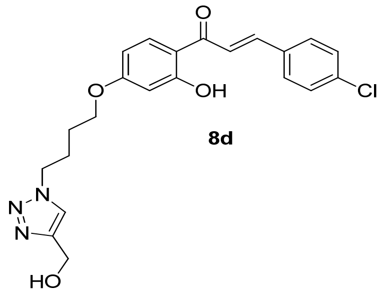

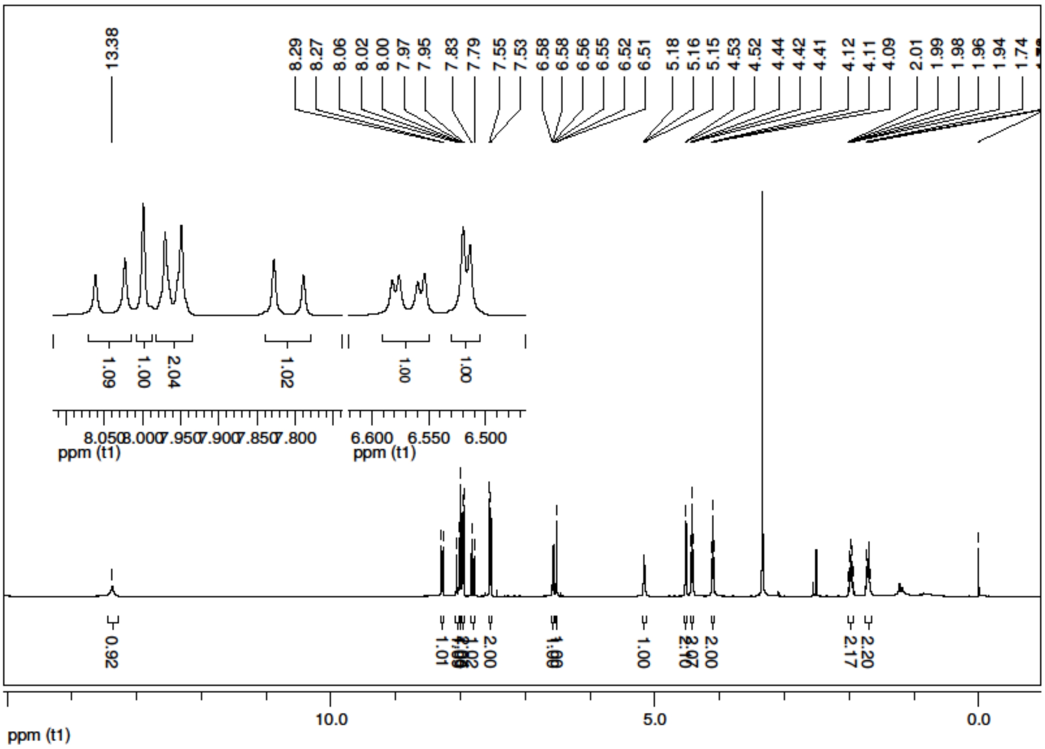

Figure S50. ^{13}C-NMR spectrum of compound **8d** (DMSO-d_6, 100 MHz).

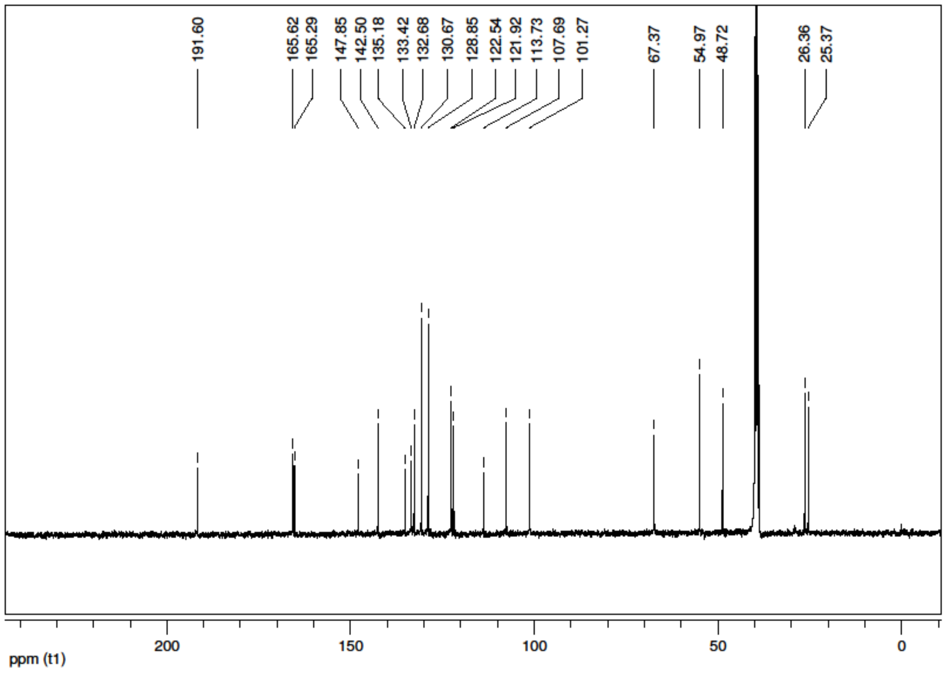

Figure S51. IR spectrum of compound **8d**.

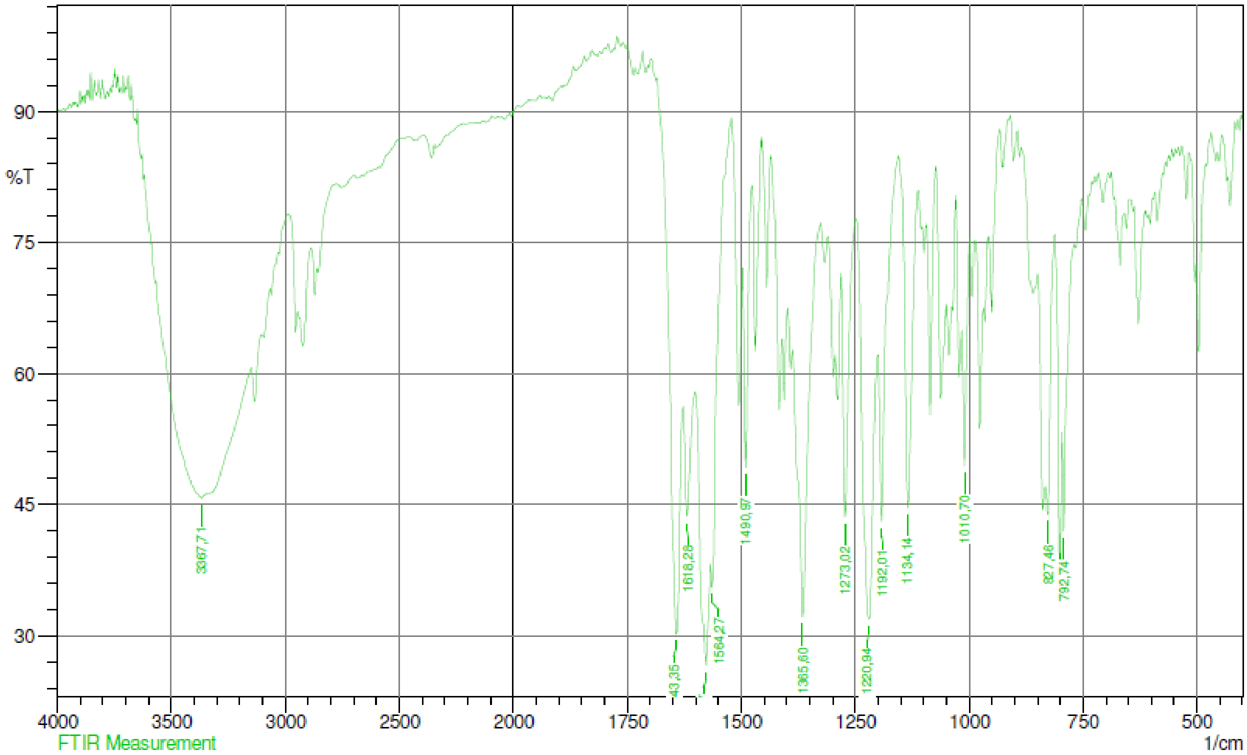

Figure S52. HRMS spectrum of compound **8d**.

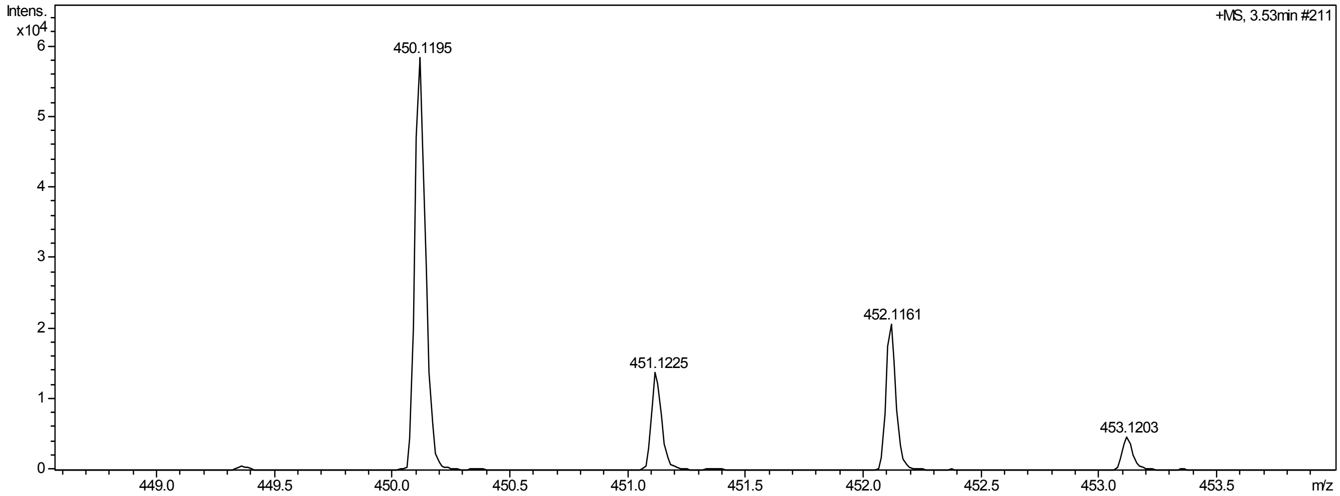

Figure S53. 1H-NMR spectrum of compound **8e (**DMSO-d_6, 400 MHz).

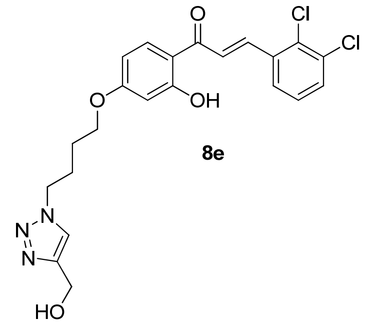

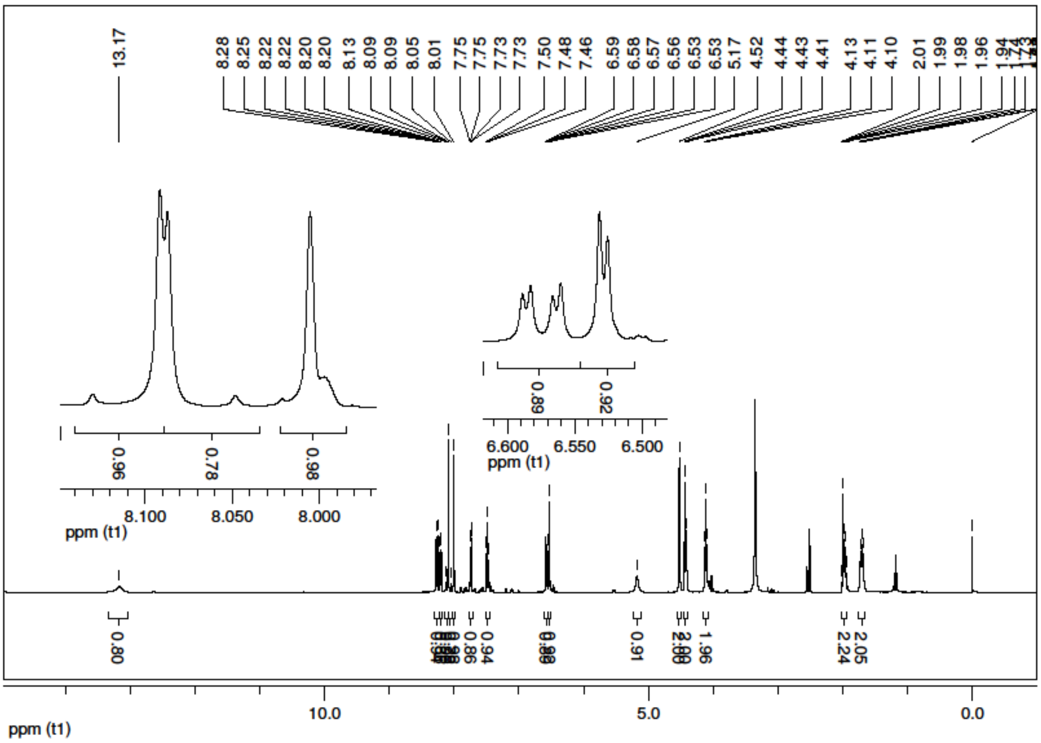

Figure S54. ^{13}C-NMR spectrum of compound **8e** (DMSO-d_6, 100 MHz).

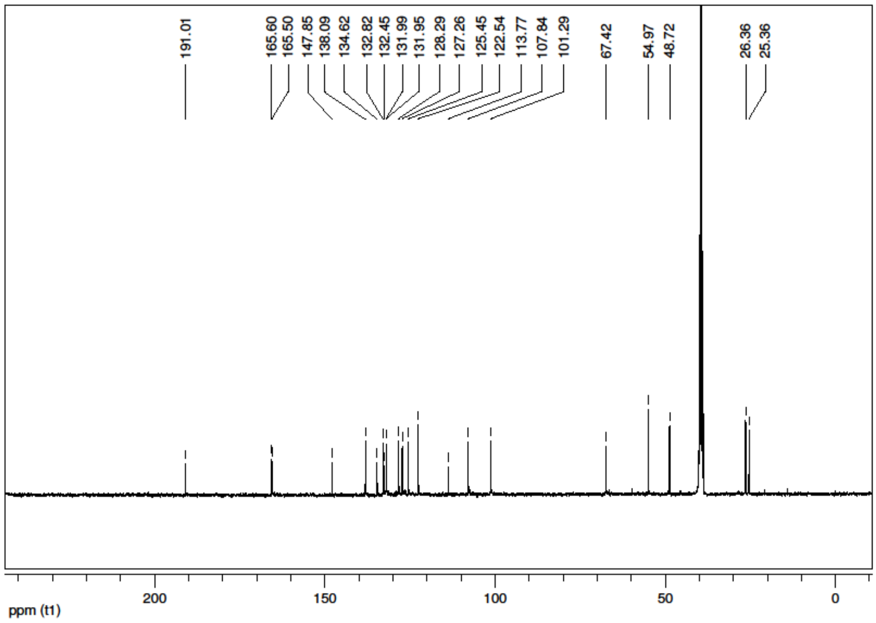

Figure S55. IR spectrum of compound **8e**.

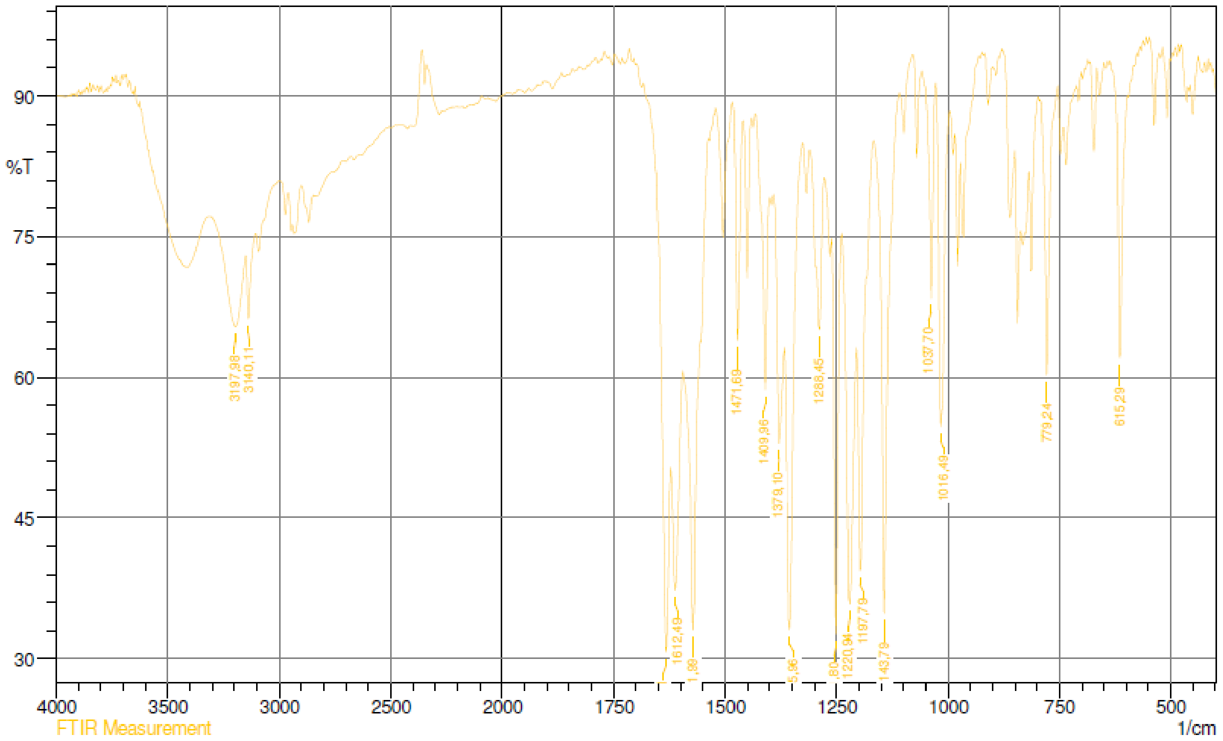

Figure S56. HRMS spectra of compound **8e**.

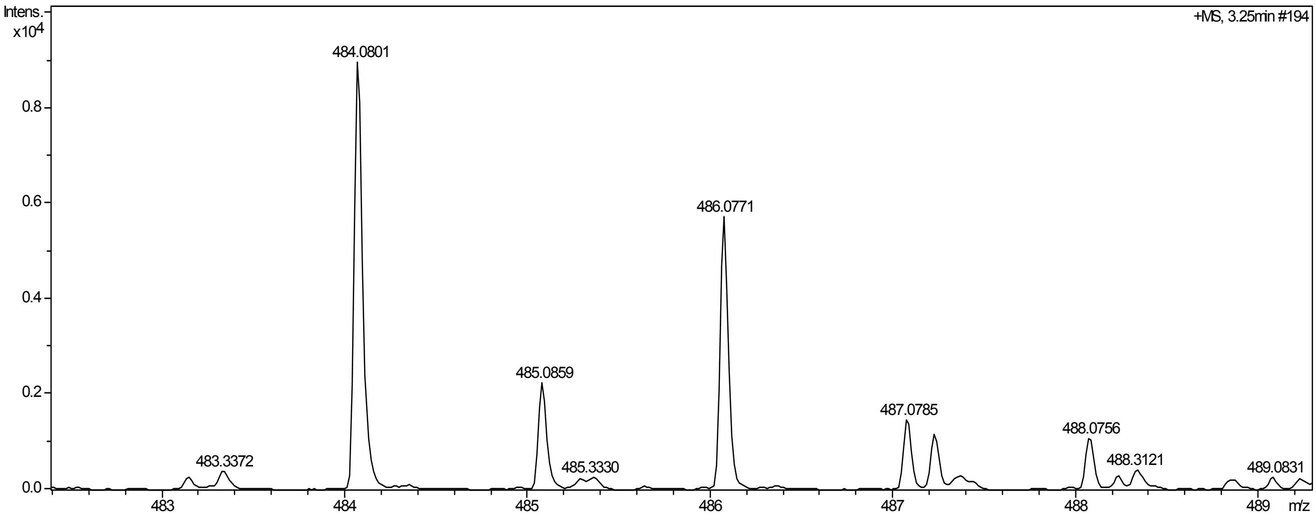

Figure S57. 1H-NMR spectrum of compound **8f** ($CDCl_3$, 400 MHz).

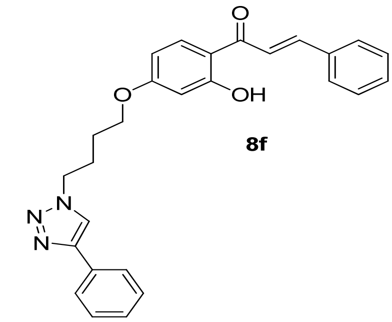

8f

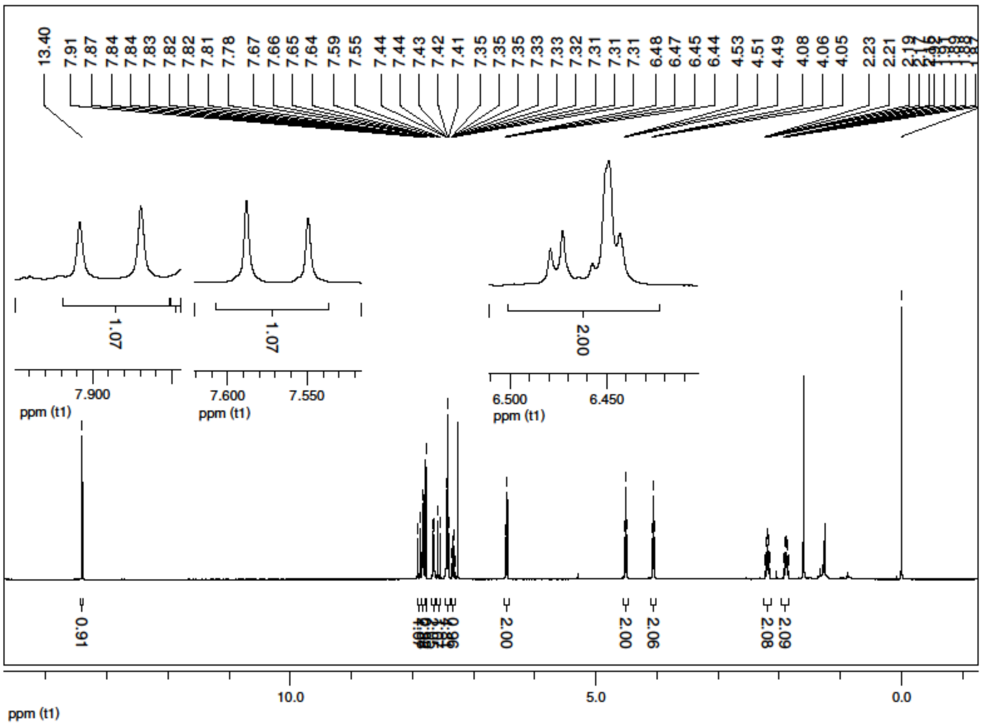

Figure S58. ^{13}C-NMR spectrum of compound **8f** ($CDCl_3$,100 MHz).

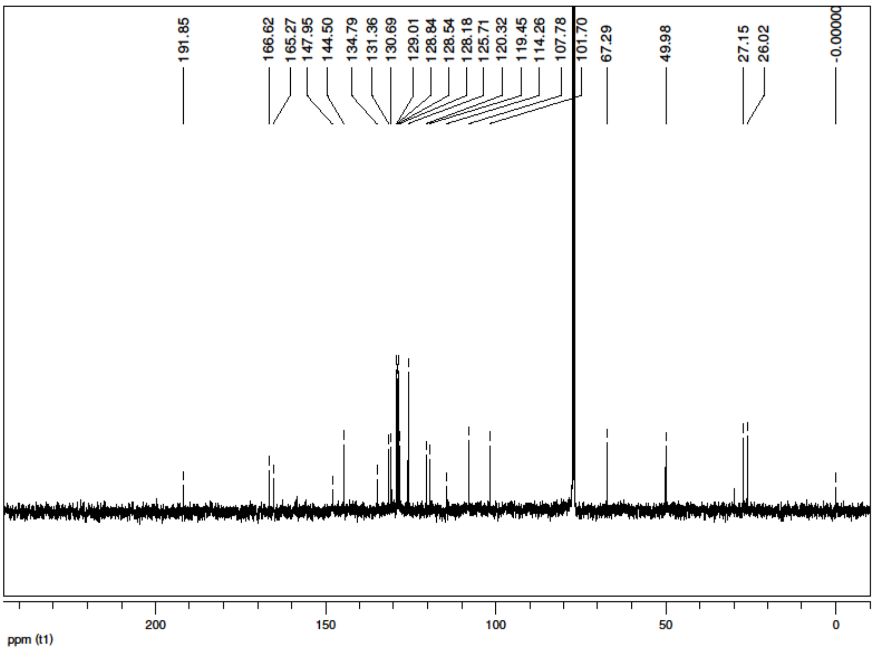

Figure S59. IR spectrum of of compound **8f**.

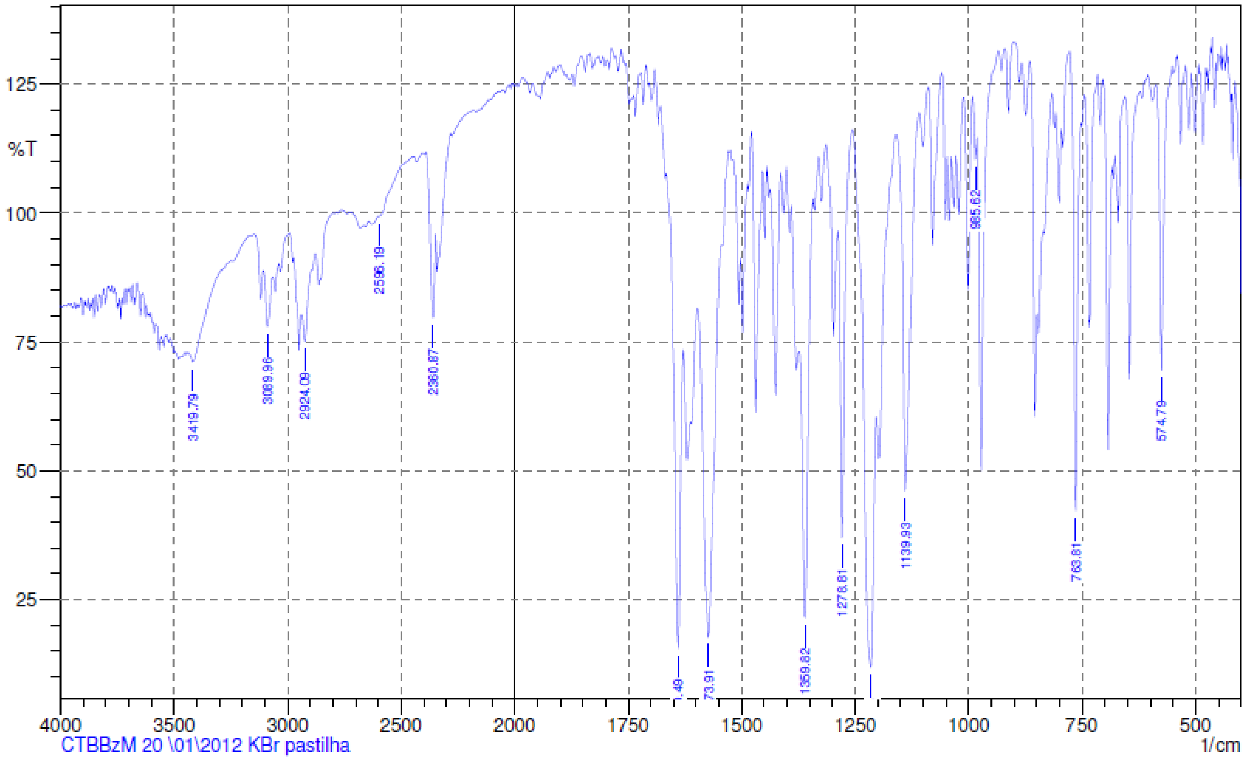

Figure S60. HRMS spectrum of compound **8f**.

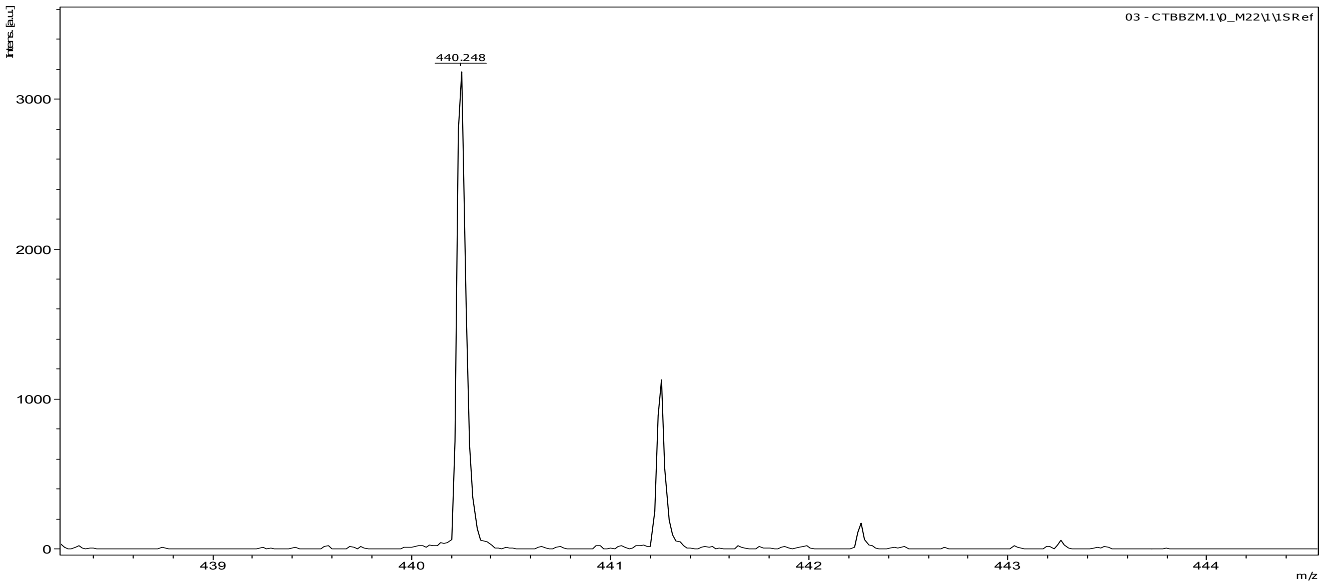

Figure 61. 1H-NMR spectrum of compound **8g** ($CDCl_3$, 400 MHz).

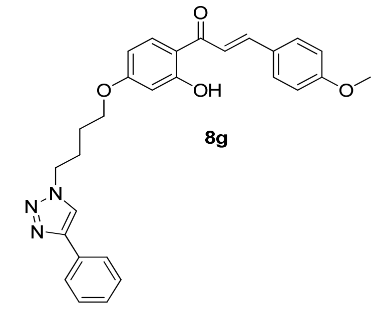

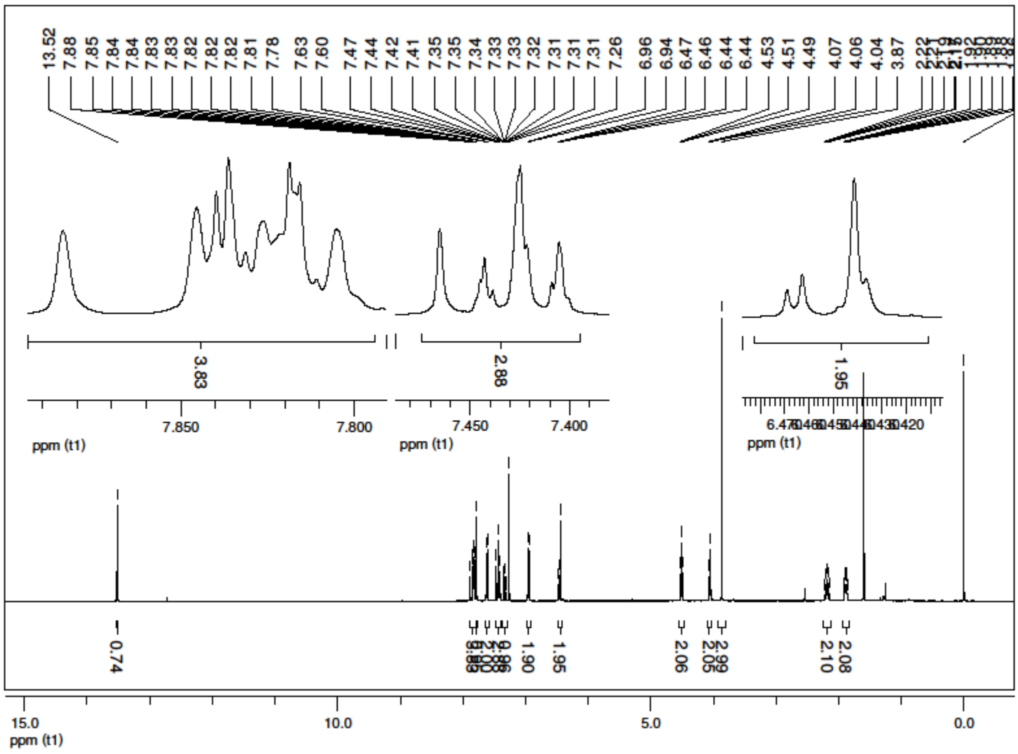

Figure S62. ^{13}C-NMR spectrum of **8g** ($CDCl_3$, 100 MHz).

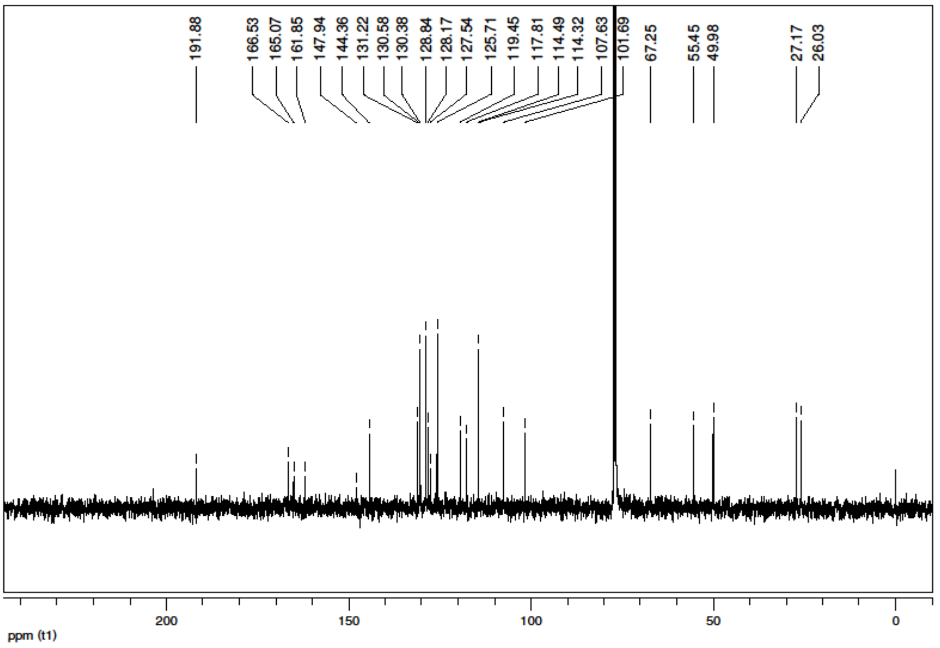

Figure S63. IR spectrum of compound **8g**.

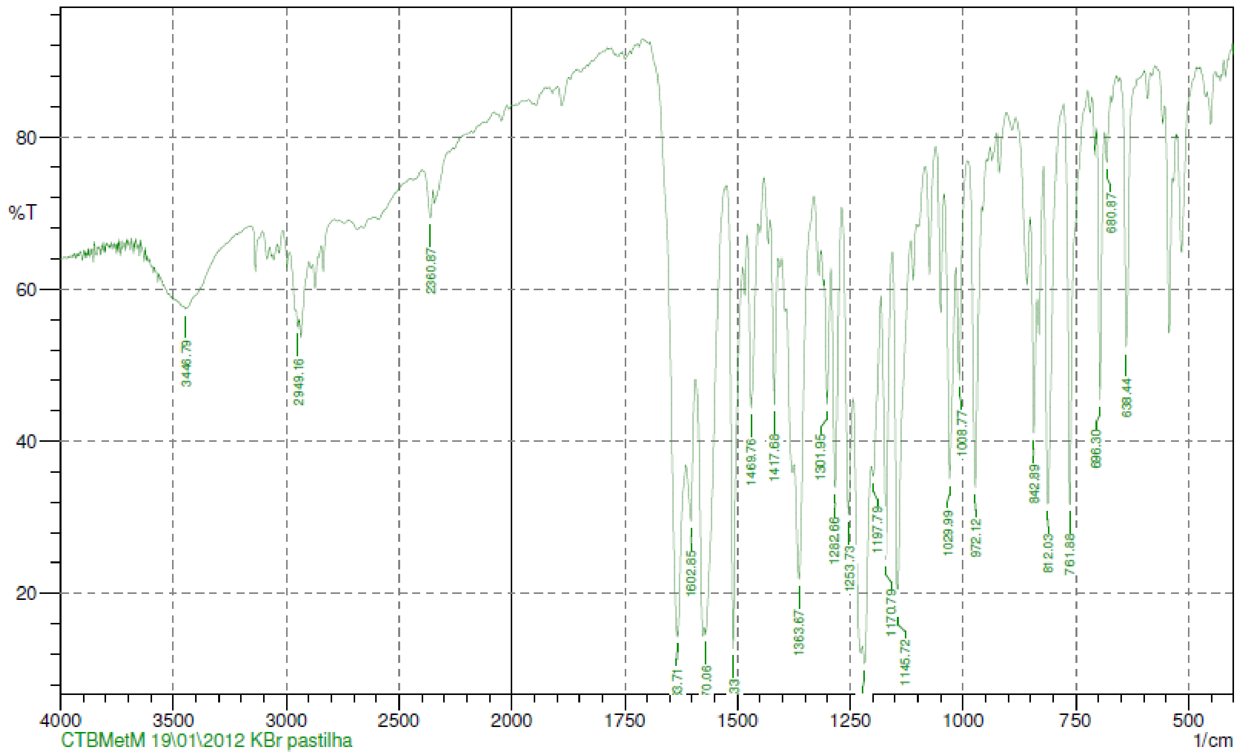

Figure S64. HRMS spectrum of compound **8g**.

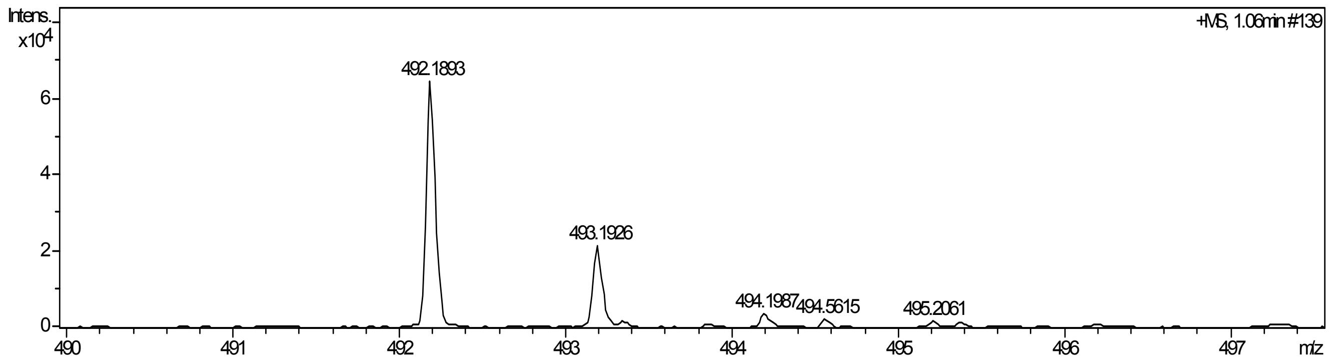

Figure S65. 1H-NMR spectrum of compound **8h** (DMSO-d_6, 400 MHz).

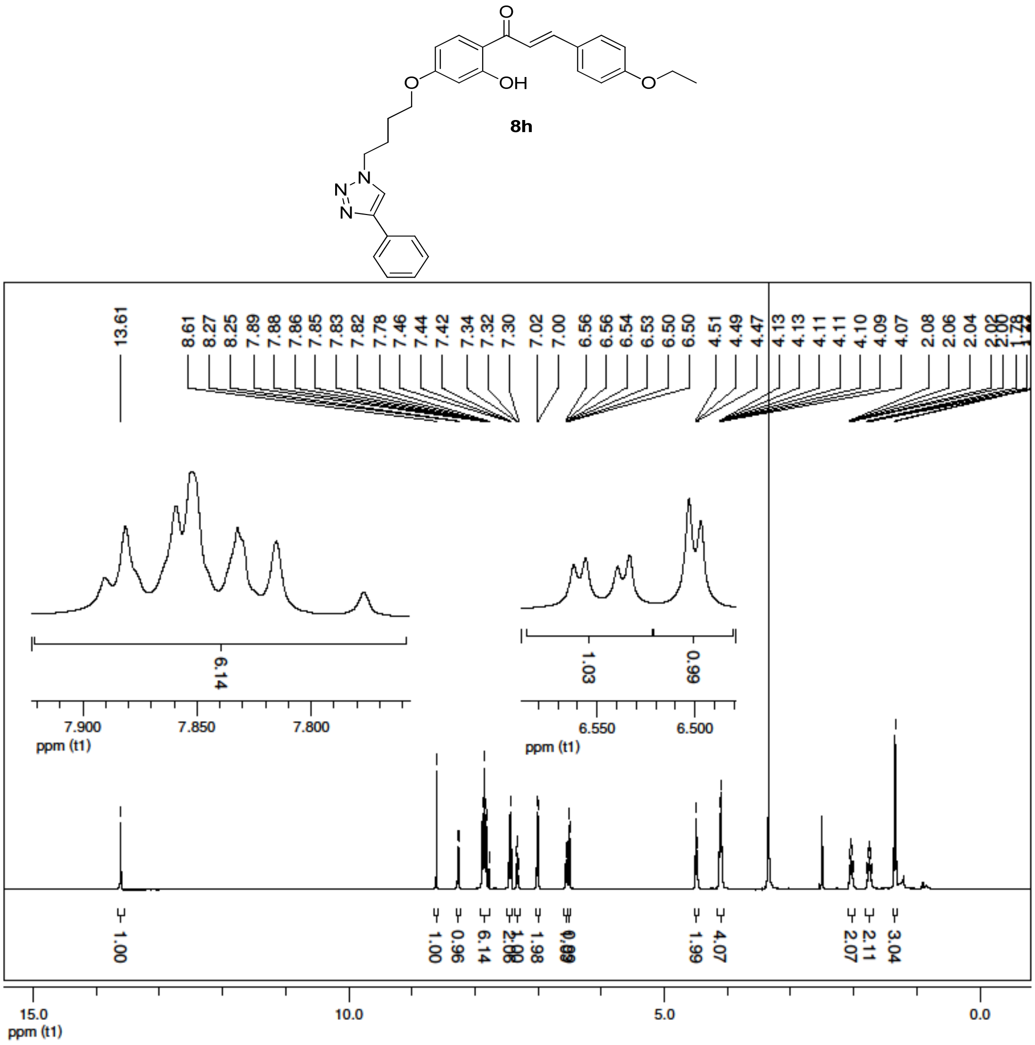

Figure S66. ^{13}C-NMR spectrum of compound **8h** (DMSO-d_6, 100 MHz).

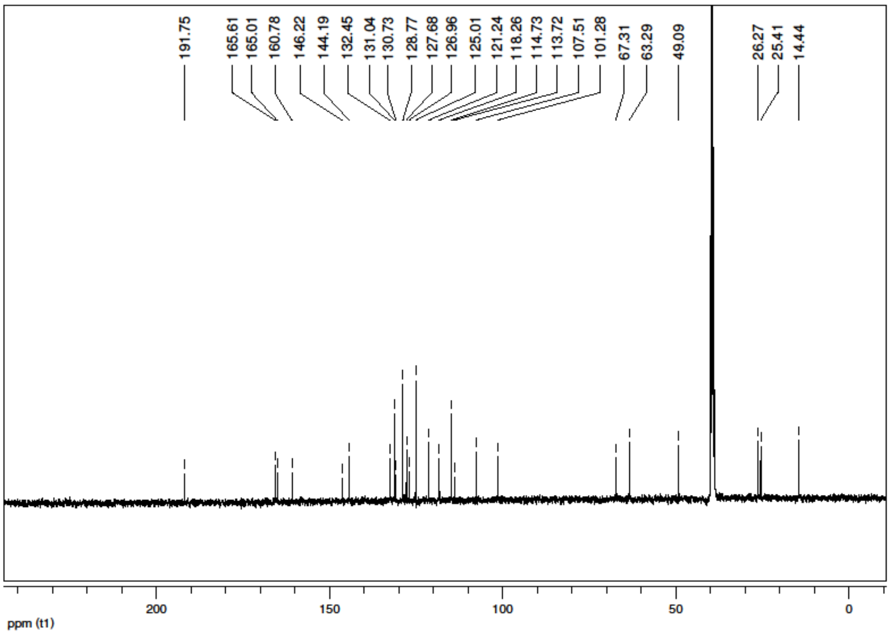

Figure S67. IR spectrum of compound **8h**.

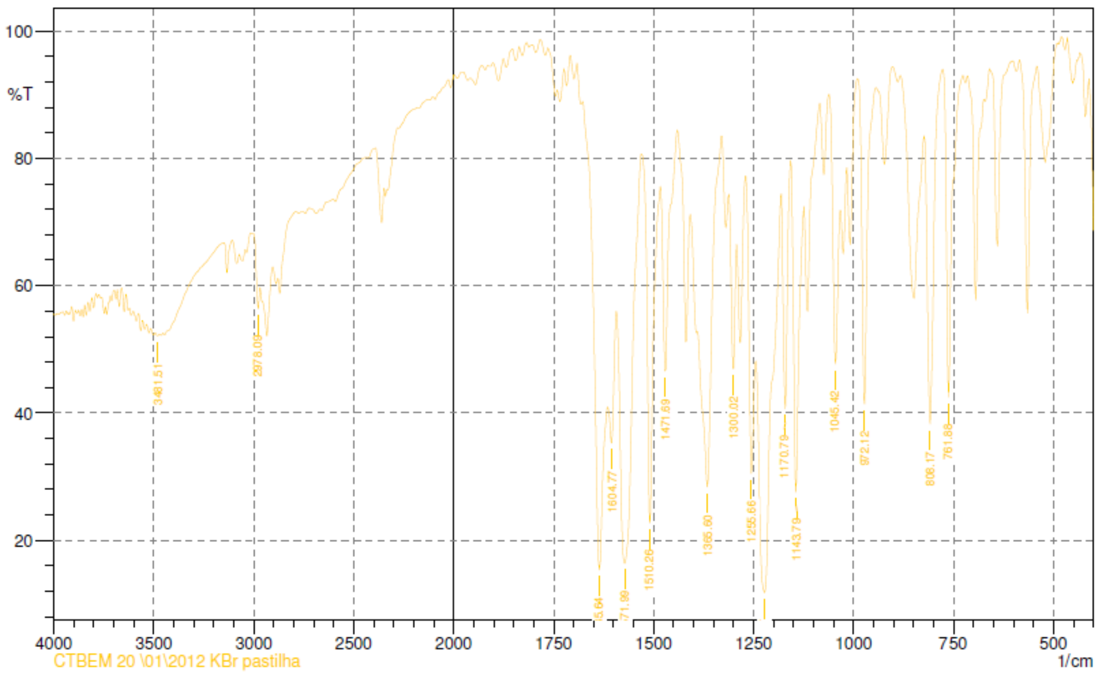

Figure S68. HRMS spectrum of compound **8h**.

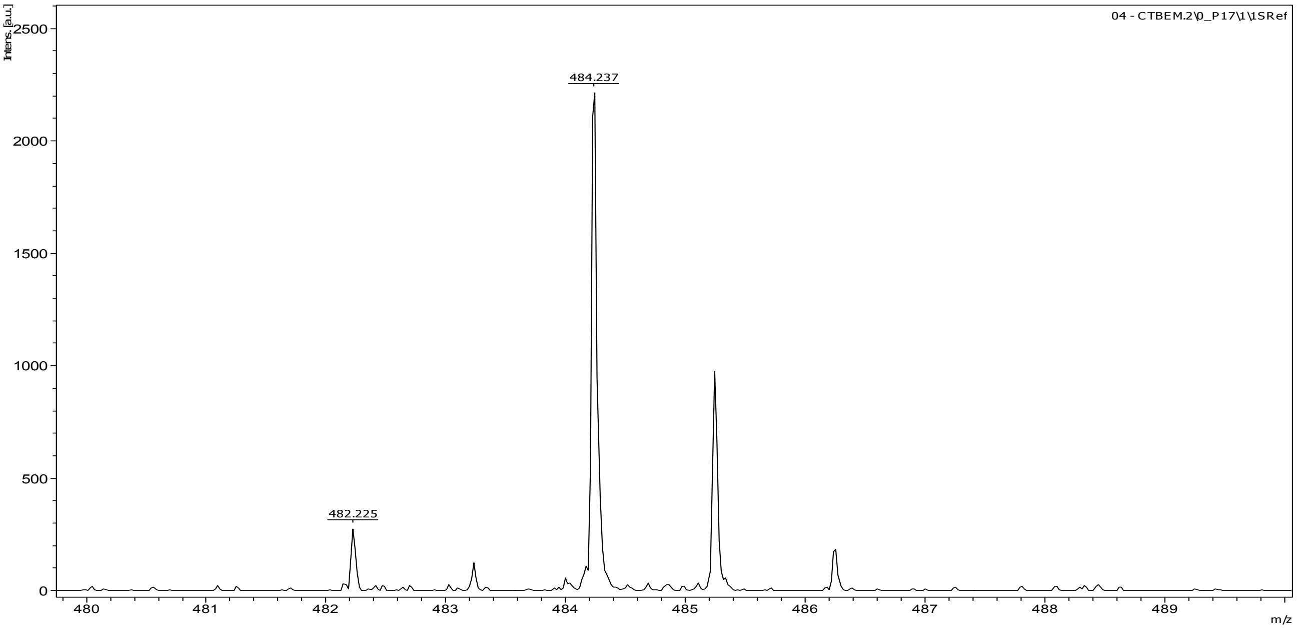

Figure S69. 1H-NMR spectrum of compound **8i** (DMSO-d_6, 400 MHz).

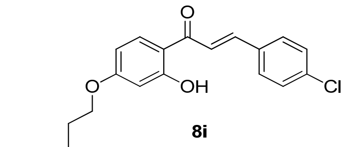

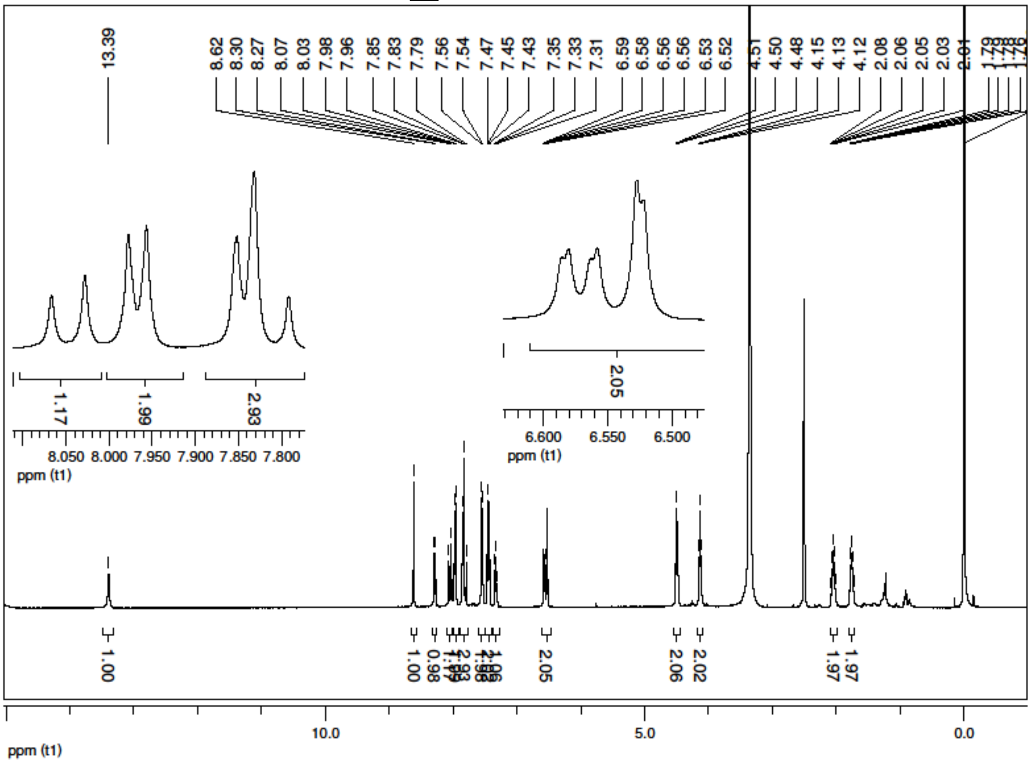

Figure 70. ^{13}C-NMR spectrum of compound **8i** (DMSO-d_6, 100 MHz).

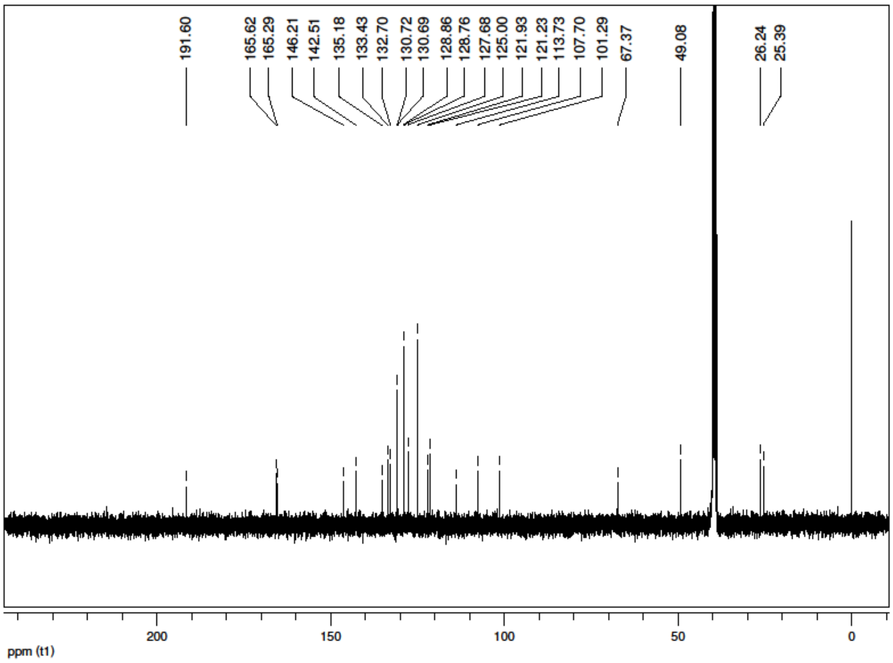

Figure S71. IR spectrum of compound **8i**.

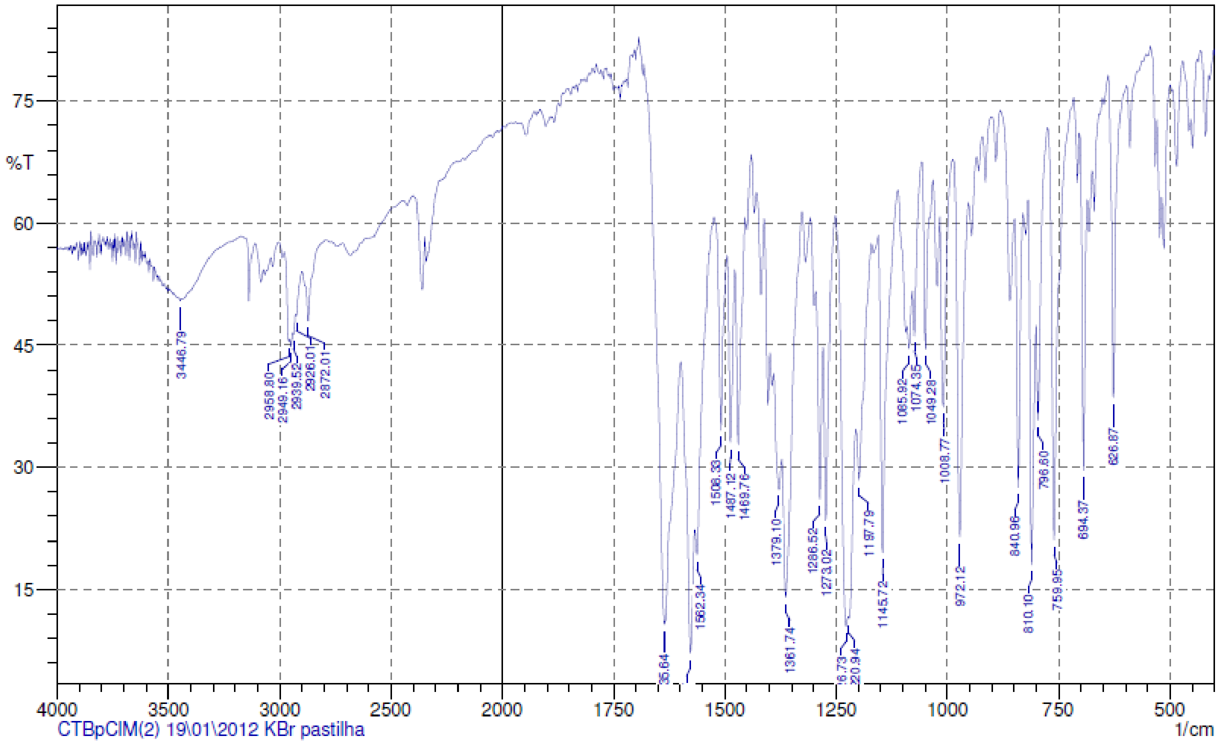

Figure S72. HRMS spectrum of compound **8i**.

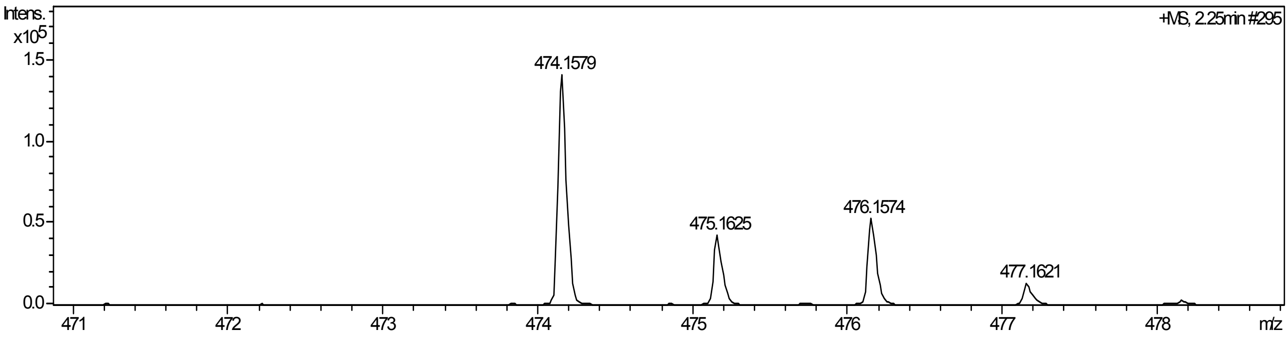

Figure S73. 1H-NMR spectrum of compound **8j** ($CDCl_3$, 400 MHz).

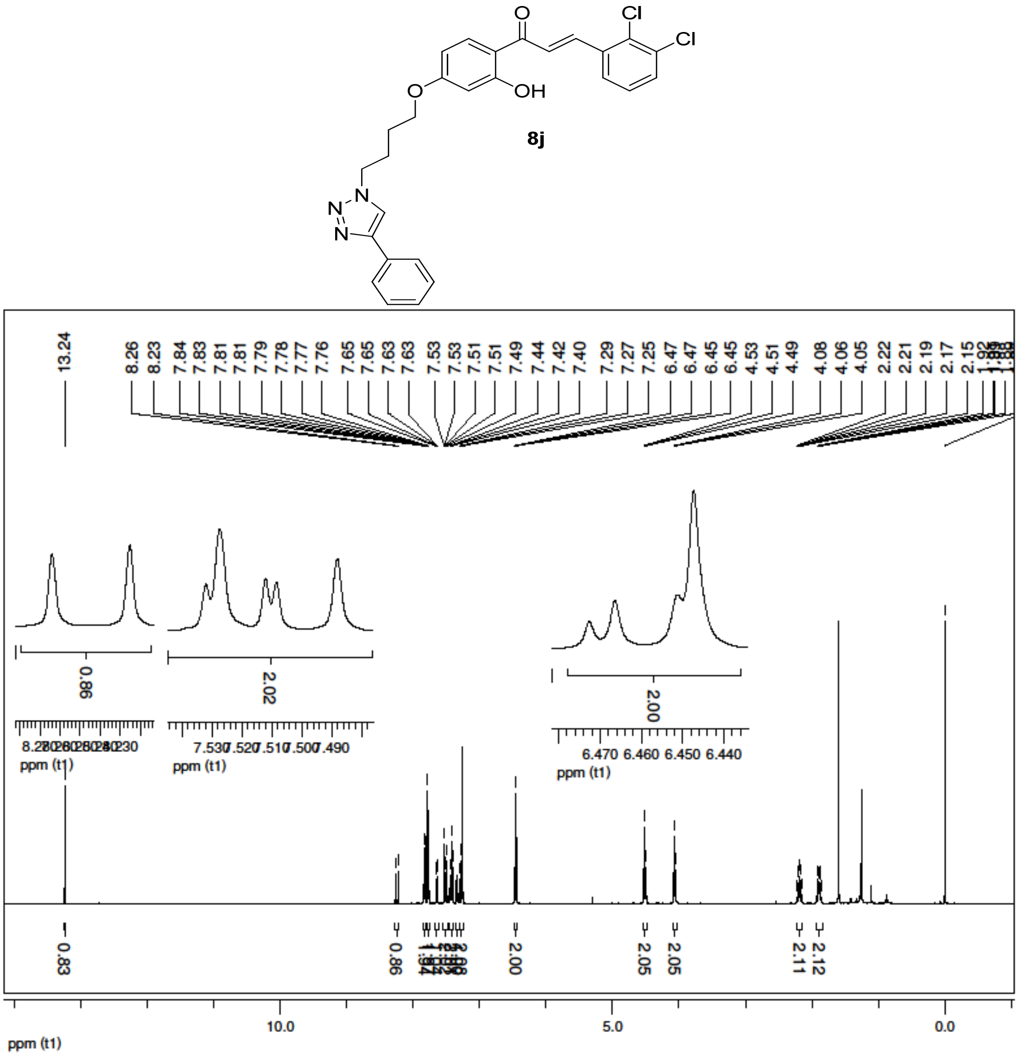

Figure S74. ^{13}C-NMR spectrum of compound **8j** ($CDCl_3$, 100 MHz).

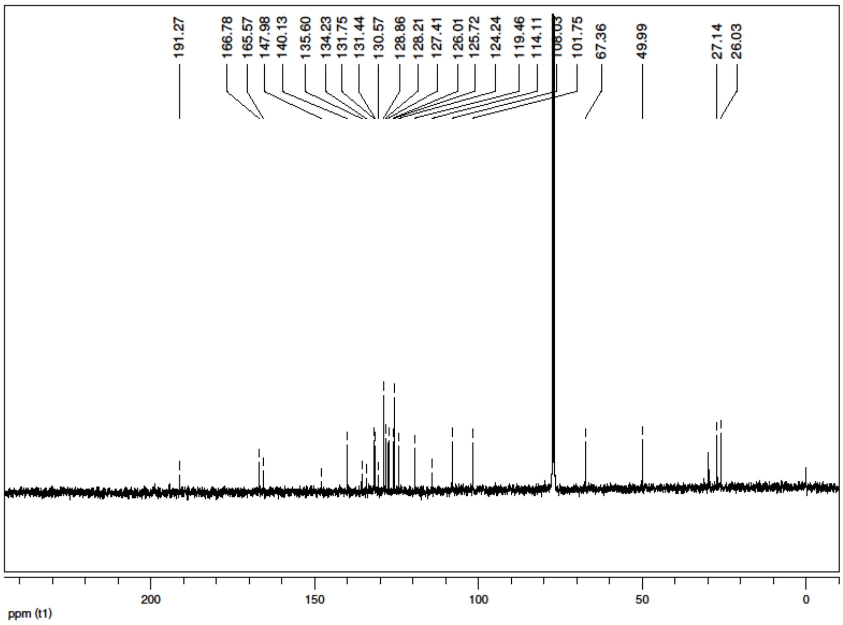

Figure S75. IR spectrum of compound **8j**.

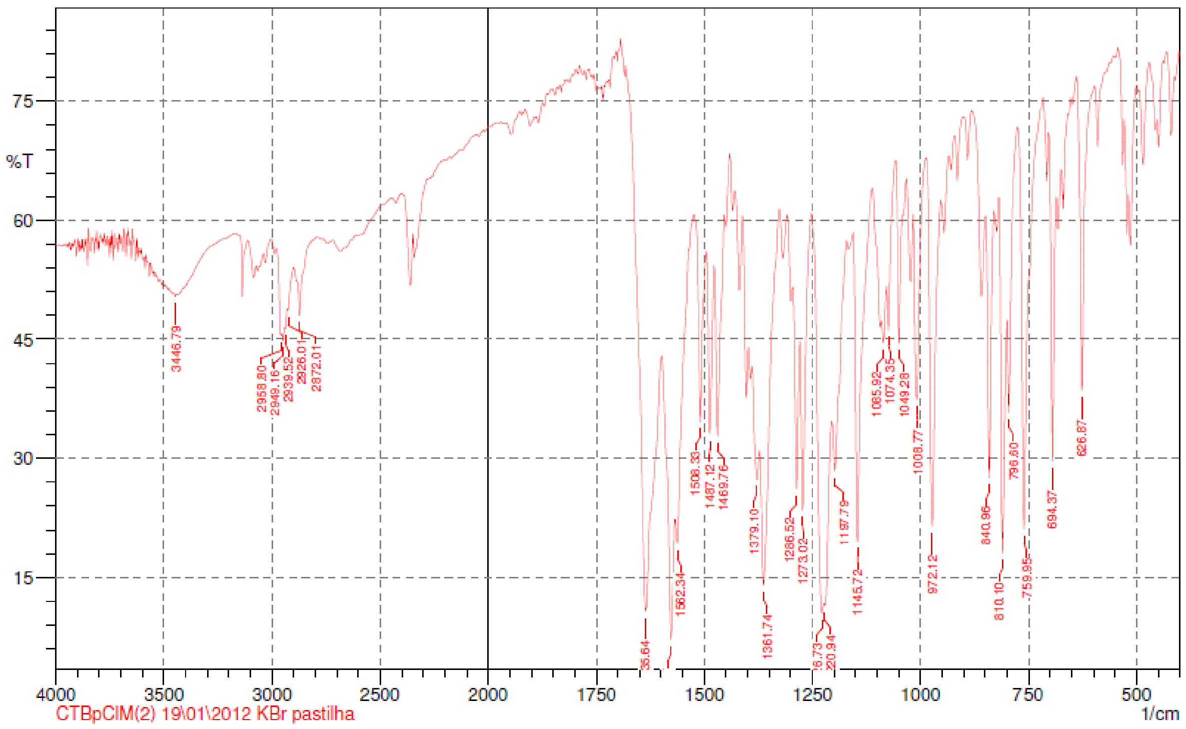

Figure S76. HRMS spectrum of compound **8j**.

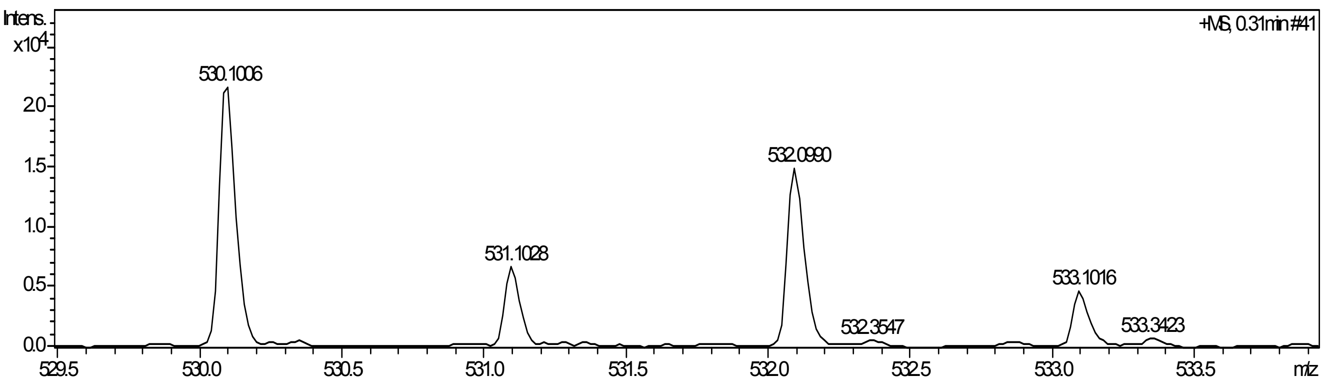

Supplement: Supplementary file 1 [file molecules-17-10331-s001.pdf]
